# An improved Coati Optimization Algorithm with multiple strategies for engineering design optimization problems

**DOI:** 10.1038/s41598-024-70575-4

**Published:** 2024-09-03

**Authors:** Zhang Qi, Dong Yingjie, Ye Shan, Li Xu, He Dongcheng, Xiang Guoqi

**Affiliations:** 1 Chengdu Technological University, Chengdu, 611730 China; 2 Panzhihua Hangyou New Material Technology Co., Ltd., Panzhihua, 617000 China; 3 Panzhihua University, Panzhihua, 617000 China

**Keywords:** COA, Chaotic sequence, Nonlinear inertia weight, Adaptive T-distribution variation strategy, Alert updating strategy, CEC2017, Engineering design optimization problems, Mathematics and computing, Engineering

## Abstract

Aiming at the problems of insufficient ability of artificial COA in the late optimization search period, loss of population diversity, easy to fall into local extreme value, resulting in slow convergence and lack of exploration ability; In this paper, an improved COA algorithm based on chaotic sequence, nonlinear inertia weight, adaptive T-distribution variation strategy and alert updating strategy is proposed to enhance the performance of COA (shorted as TNTWCOA). The algorithm introduces chaotic sequence mechanism to initialize the position. The position distribution of the initial solution is more uniform, the high quality initial solution is generated, the population richness is increased, and the problem of poor quality and uneven initial solution of the Coati Optimization Algorithm is solved. In exploration phase, the nonlinear inertial weight factor is introduced to coordinate the local optimization ability and global search ability of the algorithm. In the exploitation phase, adaptive T-distribution variation is introduced to increase the diversity of individual population under low fitness value and improve the ability of the algorithm to jump out of the local optimal value. At the same time, the alert update mechanism is proposed to improve the alert ability of COA algorithm, so that it can search within the optional range. When Coati is aware of the danger, Coati on the edge of the population will quickly move to the safe area to obtain a better position, while Coati in the middle of the population will randomly move to get closer to other Coatis. IEEE CEC2017 with 29 classic test functions were used to evaluate the convergence speed, convergence accuracy and other indicators of TNTWCOA algorithm. Meanwhile, TNTWCOA was used to verify 4 engineering design optimization problems, such as pressure vessel optimization design and welding beam design. The results of IEEE CEC2017 and engineering design Optimization problems are compared with Improved Coati Optimization Algorithm (ICOA), Coati Optimization Algorithm (COA), Golden Jackal Optimization Algorithm (GJO), Osprey Optimization Algorithm (OOA), Sand Cat Swarm Optimization Algorithm (SCSO), Subtraction-Average-Based Optimizer (SABO). The experimental results show that the improved TNTWCOA algorithm significantly improves the convergence speed and optimization accuracy, and has good robustness. Three‑bar truss design problem, The Gear Train Design Problem, Speed reducer design problem shows a strong solution advantage. The superior optimization ability and engineering practicability of TNTWCOA algorithm are verified.

## Introduction

Coati Optimization Algorithm (COA) is a novel metaheuristic algorithm created by Dehghani in 2023^1^, COA algorithm, which simulates Coati's hunting behavior, has the characteristics of strong searching ability and fast convergence speed. But, due to the random generation of the initial population, it lacks diversity, random strategy was adopted in the foraging stage of dung beetles, and the adaptive ability was lacking, location update of dung beetles' stealing behavior depended on the current optimal value, and the population diversity was lacking, those results in the unbalanced global exploration and local development ability of DBO algorithm, which is easy to fall into local optimal solution, weak global exploration ability, and low convergence accuracy efficiency. So, COA algorithm, as one of metaheuristic algorithms (MAs), has the commonality of all meta-heuristic algorithms. The search process can be divided into two stages: exploration and exploitation. The exploration stage refers to the algorithm's ability to search the global space, which determines whether the algorithm can get the optimal solution. Exploitation phase refers to the ability to search local space, which determines the speed of the algorithm to obtain the optimal solution. The better the balance between exploration and exploitation, the better the performance of the algorithm. But according to the “No Free Lunch” theory^2^, there is no metaheuristic algorithm can solve all optimization problems, the most important factor is that is metaheuristic algorithms existed the problem of falling into local optima, most MAs algorithms suffer from being trapped in local optima^3^.

In order to solve this problem, experts and scholars have begun to propose different types of strategies to solve this problem. Fan et al. proposed an improved African vultures optimization algorithm based on tent chaotic mapping and time-varying mechanism (TAVOA) to further improve the African vultures optimization algorithm (AVOA), and the tent chaos strategy to initialize the positions of^4^. Zhang et al. proposes a chaotic adaptive sailfish optimizer with genetic characteristics (CASFO). The CASFO algorithm first introduces the Tent chaos strategy to initialize the positions of sailfish and sardines to increase the diversity of the population^5^. Ding et al. proposed an improved WOA algorithm based on the concepts of chaos initialization, nonlinear convergence factor, and chaotic inertial weight to enhance its exploration abilities^6^. Li et al. proposed to integrate Tent chaotic mapping, nonlinear inertia weights and Gauss-Cauchy hybrid mutation strategy into the Gull algorithm to improve the computational accuracy and convergence speed of the algorithm^7^. Liu et al. proposed an improved butterfly optimization algorithm based on nonlinear inertial weight, bidirectional differential mutation strategy with decision coefficient and disturbance factor, bidirectional differential mutation strategy with decision coefficient and disturbance factor to improve the convergence speed and optimization accuracy of the butterfly optimization algorithm^8^. Cao et al. proposed an improved moth algorithm, which combines the adaptive crossover operator with the Lévy flight strategy, introduces an adaptive t-distribution variation in flight straight strategy, and uses the greedy strategy to enhance the global search capability and speed^9^. Zhu et al. proposed an improved SMA based on adaptive t-distributed variation strategy and chaotic opposition-based learning strategy to enhance the convergence speed, solution accuracy, and robustness of the SMA^10^, Liu proposed an improved Sparrow search algorithm (SSA) based on the Circle chaos mapping, T-distribution variation to enhanced the global optimization ability and convergence precision of the SSA^11^.

Aiming at the weakness of COA, Scholars have conducted in-depth analysis of this COA algorithm and put forward some improved methods. Yin et al. proposed an improved COA algorithm based on chaotic mapping, opposition learning mechanism and sine and cosine algorithm, which enhanced the flexibility and convergence speed of the algorithm^12^. Kaishi et al. involves a triple approach incorporating chaos mapping, Gaussian walk, and random walk to mitigate the randomness of the initial solution in the conventional Coati Optimization Algorithm (COA) and enhance the search capabilities through a dual population strategy, adaptive factors, and a stochastic differential variation strategy^13^. Fatma added some operators to improve the Coati Optimization Algorithm, such as adaptive s-best mutation operator to enhance the balance between exploration and exploitation, the directional mutation rule is used to open the way to discover the search space thoroughly, and the search direction is controlled toward the global best^14^.

Through the above analysis, we can find that in order to solve the problems of slow convergence speed and easy to fall into local optimization in metaheuristic algorithms, scholars mainly improve population initialization methods by introducing chaotic mapping and integrating Levy flight strategy. t-distribution variation, Gaussian walk, s-best mutation operator and other methods improve the exploration and exploitation ability of the algorithm, and obtain good results.

In order to improve the optimization speed and performance of Coati algorithm, a multi-strategy improved Coati algorithm is proposed, which combines chaotic sequence, nonlinear inertia weight, adaptive T-distribution variation strategy, alert update and other strategies to improve the optimization performance of the algorithm. The algorithm introduces chaotic sequence mechanism to initialize the position. The position distribution of the initial solution is more uniform, the high quality initial solution is generated, the population richness is increased, and the problem of poor quality and uneven initial solution of Coati Optimization Algorithm is solved. In the exploration phase, the nonlinear inertial weight factor is introduced to coordinate the local optimization ability and global search ability of Coati algorithm. In the exploitation phase, adaptive T-distribution variation is introduced to increase the diversity of individual population under low fitness value and improve the ability of the algorithm to jump out of the local optimal value. At the same time, the Coati alert update mechanism is proposed to improve the alert capability of the Coati algorithm, so that it can search within the optional range. When Coati is aware of the danger, the Coati on the edge of the population will quickly move to the safe area to obtain a better position, while the Coati in the middle of the population will move randomly to be close to others Coati. At the same time, we use IEEE CEC2017 benchmark test function to verify the optimization effect of TNTWCOA algorithm, and compare it with ICOA^3^, COA^1^, GJO^15^, OOA^16^, SCSO^17^ and SABO^18^ algorithms respectively in different dimensions (Dim = 30, Dim = 50, Dim = 100). The convergence curves, Friedman ordering test, boxplot and Wilcoxon rank sum test results of 6 algorithms in different dimensions are discussed. Finally, in order to verify the engineering practicability of TNTWCOA algorithm, this paper selects 4 engineering problems to test the optimization performance of TNTWCOA. Through the analysis of engineering problem data, it can be determined that the algorithm has application value in engineering optimization problems. In the experimental part, IEEE CEC2017 benchmark experiment is selected to test the optimization performance of TNTWCOA. In the experiment, TNTWCOA algorithm is compared with ICOA, COA, GJO, OOA, SCSO and SABO and other 7 algorithms, and the results show that TNTWCOA algorithm is better than other algorithms in terms of optimization performance. In addition, the TNTWCOA algorithm is applied to three bar truss design, The Gear Train Design, Speed reducer and other four practical constraint projects to verify the actual optimization effect of ICOA on engineering problems. In future work, we will continue to improve TNTWCOA's exploration capabilities and convergence rate.

The main contributions of this paper are as follows:

1. Chaotic sequence mechanism is introduced to initialize the position to solve the problem of poor quality and uneven initial solution of the Coati Optimization Algorithm
2. The nonlinear inertial weight factor is introduced to coordinate the local optimization ability and global search ability of the algorithm in exploration phase.
3. Adaptive T-distribution variation is introduced to increase the diversity of individual population under low fitness value and improve the ability of the algorithm to jump out of the local optimal value in the exploitation phase
4. A Coati alert update mechanism is proposed to improve the alerting ability of the Coati algorithm.

The rest of the paper is organized as follows. “Coati Optimization Algorithm” section briefly describes the theory and major steps of conventional COA algorithm. “Improved Coati Optimization Algorithm” section describes the proposed coati optimization algorithm in detail. “Experimental studies and results” section discusses the simulation results of the improved algorithm TNTWCOA and evaluates its performance. "TNTWCOA for engineering optimization problems" section discusses the results of the improved algorithm TNTWCOA, when used for solving 4 classical engineering design problems. Finally, “Conclusion” section summarizes the whole paper.

## Coati Optimization Algorithm

Coati Optimization Algorithm (COA) is a novel metaheuristic algorithm created by Dehghani, M that mimicked coati behavior in nature in 2023^1^. The core of the COA is to mimic natural actions of two coatis, including exploration (hunting and attacking iguanas), exploration (fleeing from predators) two actions^19^. The key steps for COA are introduced in the following subsection.

### Algorithm initialization process

In the initialization stage of the COA, the position of the coatis in search space is randomly generated for the COA by using the expression in Eq. (1)

1 $$ X_{i} :x_{i,j} = b_{j}^{{\text{L}}} + rand(0,1) \cdot \left( {b_{j}^{{\text{U}}} - b_{j}^{{\text{L}}} } \right),\;\;i = 1,2, \ldots ,N,\;\;j = 1,2, \ldots ,m $$

In Eq. (1): $X_{i}$ stand for the position in the search space of the $i_{th}$ coati,$x_{i,j}$ represents the value of the $j_{th}$ decision variable. $b_{j}^{{\text{L}}}$ and $b_{j}^{{\text{U}}}$ represent the upper and lower bound of the decision variables, respectively. $N$ is the coatis’ number, $m$ is the number of decision variables.

### Mathematical model of COA

#### Phase 1: Hunting and attacking strategy on iguana (exploration phase)

**Fig. 1:**
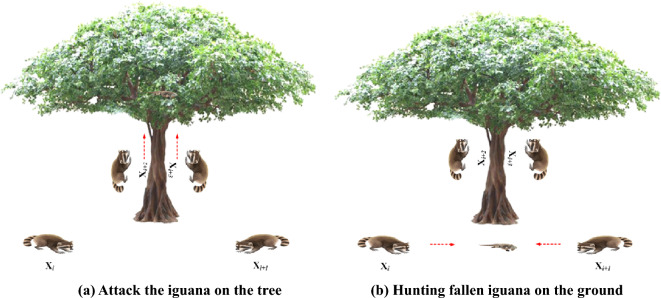
Pattern diagram of the first phase of COA.

The first phase of updating the coatis’ population in the search space is modeled based on simulating their strategy when attacking iguanas. In this strategy, a group of coatis climbs the tree to reach an iguana and scare it. Several other coatis wait under a tree until the iguana falls to the ground. After the iguana falls to the ground, the coatis attack it and hunt it. This strategy leads coatis to move to different positions in the search space, which demonstrates the COA’s exploration ability in global search in the problem-solving space. In exploration phase, Coati's position update strategy mainly simulates Coati's hunting and attacking iguana behavior. The behavior of Coati is divided into two steps to complete the hunting and attacking of the iguana. (1) Fright. A group of Coatis climb a tree to approach an iguana and scare it. (2) Several other Coatis wait under the tree, waiting for the frightened iguana to fall to the ground, and after the iguana lands, complete the attack and hunt it. As shown in Fig. 1.

This strategy causes Coati to move to different locations in the search space, which in turn shows that the COA optimization algorithm has exploration capability in solving the global search of the problem space. In the COA algorithm, it is assumed that the location of the best member of the population is that of the iguana. It is also assumed that the number of Coatis completing steps (1) and (2) is each half of the total number of Coatis. Thus, the mathematical expression of position is:

2 $$ X_{i}^{{{\text{P}}1}} :x_{i,j}^{{{\text{P}}1}} = x_{i,j} + rand(0,1)\left( {G_{j} - Ix_{i,j} } \right),i = 1,2, \cdots ,\left[ \frac{N}{2} \right];j = 1,2, \cdots ,m $$

where,$X_{i,j}^{{{\text{P}}1}}$ is the new position of the ith Coati in the jth dimension; r is the random number between [0,1]; $G_{j}$ is the iguana's position in the jth dimension, which actually refers to the position of the best member; I is a number randomly selected from the set {1,2}; N is the number of Coati; [N/2] is the largest integer not exceeding [N/2]; m is the number of decision variables.

After the iguana falls to the ground, it is placed in a random position in the search space. Based on this random location, the Coati on the ground moves through the search space. This step is simulated by two formulas.

3 $$ G_{{}}^{{\text{g}}} :G_{j}^{{\text{g}}} = b_{j}^{{\text{L}}} + rand(0,1)\left( {b_{j}^{{\text{U}}} - b_{j}^{{\text{L}}} } \right) $$

where:$G_{j}^{{\text{g}}}$ is the position of the iguana on the ground in the j dimension.

4 $$X_i^{{\text{P}}1}:x_{i,j}^{{\text{P}}1} = \left\{ {\begin{array}{ll}   {{x_{i,j}} + rand\left( {0,1} \right) \cdot \left( {G_j^g - I{x_{i,j}}} \right),} & \quad {F_{{\text{G}},j}^{\text{g}} \le {F_{i,j}}}  \\    {{x_{i,j}} + rand\left( {0,1} \right) \cdot \left( {{x_{i,j}} - G_j^g} \right),} & \quad {{\text{other}}}  \\  \end{array}} \right.$$

$$ i = [N/2] + 1,[N/2] + 2, \cdots ,N,and\;j = 1,2,...,m $$

where:$F_{{{\text{G}},j}}^{{\text{g}}}$ is the objective function value of the jth dimension iguana after it falls to the ground; $F_{i,j}$ is the objective function value of the ith Coati in jth dimension. If the updated individual is better, the current individual is updated. Otherwise, leave it as it is.

5 $$ X_{i}  = \left\{ {\begin{array}{*{20}l}    {X_{i}^{{P1}} ,F_{i}^{{P1}}  \le F_{i} } \hfill  \\    {X_{i} ,{\text{ other }}} \hfill  \\   \end{array} } \right. $$

where:$F_{i}^{{{\text{P}}1}}$ is the objective function value of the ith Coati at the new position; $F_{i}$ is the objective function value of the ith Coati at the previous position.

#### Phase 2: The process of escaping from predators (exploitation phase)

**Fig. 2:**
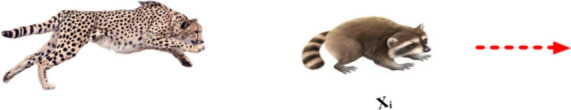
Pattern diagram of coati escaping from a predator in the second phase of COA.

In exploitation phase, Coati's location-updating strategy mainly mimics Coati's natural behavior when encountering predators and when fleeing from predators. As shown in Fig. 2.

In exploitation phase, When a predator attacks Coati, Coati flees its position. Coati's move on this strategy resulted in it being in a safe position close to its current position. This demonstrates the exploitation of COA algorithms in local search. To simulate this behavior, a random location is generated near the location of each Coati based on the following equation.

6 $$ b_{{j, {\text{L}}}}^{{{\text{loc}}}} = \frac{{b_{j}^{{\text{L}}} }}{t},b_{{j,{\text{U}}}}^{{{\text{loc}}}} = \frac{{b_{j}^{{\text{U}}} }}{t},t = 1,2, \cdots ,T $$

where: $b_{{j, {\text{L}}}}^{{{\text{loc}}}}$ is the local lower bound of the jth decision variable, $b_{{j,{\text{U}}}}^{{{\text{loc}}}}$ is the local upper bound of the jth decision variable, and t is the number of iterations; T is the maximum number of iterations.

7 $$ \left\{ {\begin{array}{*{20}l} {X_{i,j}^{{{\text{P}}2}} = x_{i,j} + (1 - 2r)\left( {b_{{j, {\text{L}}}}^{{{\text{loc}}}} + r\left( {b_{{j,{\text{U}}}}^{{{\text{loc}}}} - b_{{j, {\text{L}}}}^{{{\text{loc}}}} } \right)} \right)} \hfill \\ {i = 1,2, \cdots ,N} \hfill \\ \end{array} } \right. $$

where:$X_{i,j}^{{{\text{P}}2}}$ is the new position of the ith Coati in the jth dimension. If the updated individual is better, update the current individual, otherwise leave it as it is.8

## Improved Coati Optimization Algorithm

### Chaotic mapping strategy for algorithm initialization process

**Fig. 3:**
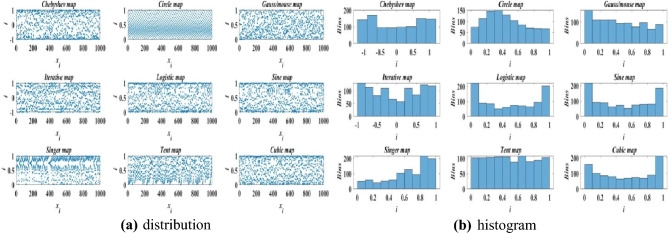
Results of population samples under different chaotic maps.

Because the individual positions of the original Coati Optimization Algorithm are generated randomly, the diversity of the population is likely to be lost, and its uniform distribution in the solution space cannot be guaranteed, this makes the algorithm easily fall into local optimization. Uniform population can speed up convergence^20,21^. Therefore, it is necessary to improve the population initialization method of the algorithm. Chaotic mapping is ergodic and stochastic. If chaotic mapping function is used to generate chaotic sequence as the initial position of population individuals to make the population distribution more uniform and avoid population uniformity, thus improving the search efficiency. Commonly used chaotic mappings are as follows: Chebyshev map, Circle map, Gauss map, Iterative map, Logistic map, Sine map, Singer map, Tent map, Cubic map. The population distribution results generated by the commonly used chaotic mappings methods is shown in Fig. 3a, and the histogram is shown in Fig. 3b.

As shown in Fig. 3, the population distribution generated by tent chaos has the best uniformity among the above major chaotic maps. Therefore, this paper chooses TENT Chaos to improve the distribution quality of the initial population in search space, and the global search ability can be strengthened, so as to improve the solution efficiency of the algorithm, and Eq. (1) can be rewritten as:

9 $$ X_{i} :x_{i,j} = b_{j}^{{\text{L}}} + \left( {b_{j}^{{\text{U}}} - b_{j}^{{\text{L}}} } \right) \cdot z_{i} $$

The expression of the tent mapping was shown in Eq. (9), $\alpha = 0.5$_._

10 $$ z_{{i + 1}}  = \left\{ {\begin{array}{*{20}c}    {\frac{{z_{i} }}{\alpha },|x_{{i,j}}  \in \left[ {0,\alpha } \right)}  \\    {\frac{{1 - z_{i} }}{{1 - \alpha }},|x_{i}  \in \left[ {\alpha ,1} \right)}  \\   \end{array} } \right. $$

### Nonlinear inertia weight factor for hunting and attacking strategy on iguana

The local optimization ability and global search ability of the coordinated meta-heuristic algorithm are the key factors that affect the optimization accuracy and optimization speed of the algorithm. Since the update of Coati individual position is closely related to the current Coati position, the nonlinear inertia weight factor is used to adjust the correlation between the update of Coati position and the current Coati position information. The calculation method of the nonlinear inertia weight factor is as follows.

11 $$ \omega = {{e^{\frac{t}{T}} - 1} \mathord{\left/ {\vphantom {{e^{\frac{t}{T}} - 1} {e - 1}}} \right. \kern-0pt} {e - 1}} $$

where, $t$ is the current iteration number and $T$ is the maximum iteration number. The maximum inertia weight $\omega_{\max } = 1$, with the progress of iteration, the inertia weight factor will increase nonlinear and eventually reach a large value.

The improved Coati calculation formula is as follows^22^:

12 $$ X_{i}^{{{\text{P}}1}} :x_{i,j}^{{{\text{P}}1}} = \omega \cdot x_{i,j} + rand(0,1)\left( {G_{j} - Ix_{i,j} } \right),i = 1,2, \cdots ,\left[ \frac{N}{2} \right];j = 1,2, \cdots ,m $$

$$ X_{i}^{{{\text{Pl}}}} :x_{i,j}^{{{\text{Pl}}}} = \left\{ {\begin{array}{ll} {\omega \cdot x_{i,j} + rand(0,1) \cdot \left( {G_{j}^{{\text{g}}} - Ix_{i,j} } \right),F_{{{\text{G}},j}}^{{\text{g}}} \le F_{i,j} } \\ {\omega \cdot x_{i,j} + rand(0,1) \cdot \left( {x_{i,j} - G_{j}^{{\text{g}}} } \right),{\text{else}}\;\;\;\;\;\;\;} \\ \end{array} } \right.,i = \left[ \frac{N}{2} \right],2, \cdots ,N;j = 1,2, \cdots ,m $$

**Fig. 4:**
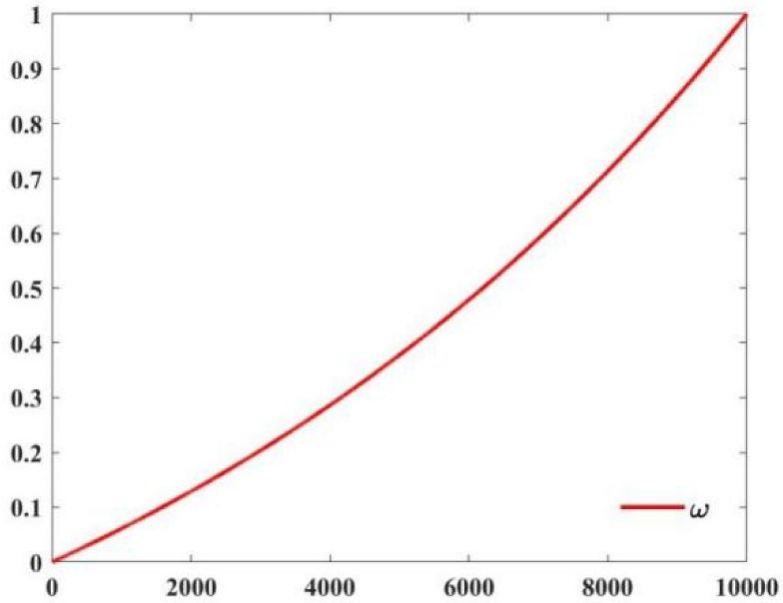
Change of nonlinear inertia weight factor.

During iteration, the variation of Nonlinear Inertia Weight Factor is shown in Fig. 4. Nonlinear Inertia Weight Factor starts from 0 and increases to 1. According to Fig. 4 and Eq. (3). At the beginning of the iterative solution, the Nonlinear Inertia Weight Factor is small, and the update of the position of the seeking individual is less affected by the current Coati position, which is conducive to the search of the algorithm in a larger scope and improve the global development ability of the algorithm. With the advancement of the optimization process, the value of $\omega$ gradually increases to 1, and the update of the position of the optimization individual becomes more influenced by the current Coati position. Narrowing the optimization range of the algorithm helps the algorithm search for the optimal solution, which not only improves the local exploration ability of the algorithm, but also improves the convergence speed of the algorithm.

### Adaptive T-distribution variation strategy for process of escaping from predators

In the exploitation phase of Coati algorithm, an adaptive T-distribution variation strategy is introduced. In each iteration, the relationship between the current Coati fitness value and the average fitness value of the population was compared. When the Coati fitness value is higher than the average fitness value of the population, it indicates that the current Coati is in an aggregation state. In this case, adaptive T-distribution variation strategy is adopted to increase Coati diversity. When the Coati fitness value is lower than the average fitness value of the population, the original Coati location updating method is used. The improved formula is as follows:

13 $$ \begin{aligned} x_{i,j}^{P2} & = G_{j} + \, G_{j} { *}f(t), \cdots F_{i}^{P2} > F_{AVG} \hfill \\ x_{i,j}^{P2} & = x_{i,j} + (1 - 2r) \cdot \left( {lb_{j}^{{\text{local }}} + r \cdot \left( {ub_{j}^{{\text{local }}} - lb_{j}^{{\text{local }}} } \right)} \right), \cdots else \hfill \\ \end{aligned} $$

The T-distribution contains a degree of freedom parameter, and its probability density is shown as follows^23^:

14 $$ f(t,n) = \frac{{\Gamma \left( {\frac{n + 1}{2}} \right)}}{{(\pi n)^{\frac{1}{2}} \Gamma \left( \frac{n}{2} \right)}} \times \left( {1 + \frac{{t^{2} }}{n}} \right)^{{ - \frac{n + 1}{2}}} , - \infty < t < \infty $$

When t(n → 1), t-distribution is Cauchy(0,1):

15 $$ f(t,1) = \frac{1}{{\pi (1 + t^{2} )}} $$

When t(n → ∞), t-distribution is Norm(0,1):

16 $$ f(t,n) \to \frac{1}{{\sqrt {2\pi } }}e^{{ - \frac{{t^{2} }}{2}}} , - \infty < t < \infty $$

where, t is the degree of freedom parameter, n is the degree of freedom, Γ() is the gamma function. When t(n → ∞) → N(0,1), t(n → 1) = C(0,1), N(0,1)as the Gaussian distribution, C(0,1) for the Cauchy distribution.

In TNTWCOA, the position of each COATI is perturbed using t-distribution mutation with adaptive parameters. t-distribution mutation operator is mathematically formulated as:

17 $$ t(x) = x\left( {1 \oplus f(t,n)} \right) $$

18 $$ t,n = e^{{4\left( {\frac{t}{T\max }} \right)^{2} }} $$

At the beginning of iteration, the T-distribution mutation is similar to Cauchy mutation, and the algorithm has a good global exploration ability, which increases the diversity of the population, and the ability to jump out of the local optimal is also enhanced. With the increase of the number of iterations, the T-distribution mutation is similar to the Gaussian mutation, which improves the local development ability of the algorithm, and the disturbance strength of the whole population changes from strong to weak. By introducing adaptive T-distribution mutation as an improved search strategy, the optimization performance of the algorithm can be effectively enhanced, and the ability of the algorithm to escape local optimal can be improved.

### Alert mechanism for process of escaping from predators

The first half of the Coati algorithm is updated using the formula of improvement point 3, and the second half is updated by introducing sparrow alert mechanism. Introducing the Coati alert update mechanism in the second stage of Coati can improve the alert capability of Coati algorithm and enable it to search within the optional range. When Coati is aware of danger, Coati on the edge of the group will quickly move to the safe area to obtain a better position. Coati in the middle of the group will randomly move around to get closer to other Coatis. The mathematical expression is as follows:

19 $$x_{i,j}^{P2} = \left\{ {\begin{array}{ll}    {{G_j} + \beta  \cdot \left| {x_{i,j}^{P2} - {G_j}} \right|,} & \quad {{\text{if}}\;{f_i} > {f_g}}  \\    {x_{i,j}^{P2} + K\left( {\frac{{\left| {x_{i,j}^t - {G_j}} \right|}}{{\left( {{f_i} - {f_w}} \right) + \varepsilon }}} \right)}, & \quad {{\text{if}}\;{f_i} = {f_g}}  \\  \end{array}} \right.$$

where: G is the current global optimal location. β, as a step control parameter, is a random number with a normal distribution of mean 0 and variance 1. K ∈ [− 1,1] indicates the direction of Coati's movement and the step size. The control parameter is a random number, and F_*i*_ is the fitness value of the current Coati individual. F_*g*_ and F_*w*_ are the best and worst global fitness values respectively. ε is a constant to avoid zeros in the denominator.

In short, F_*i*_ > F_*g*_ indicates that the Coati is at the edge of the group and is vulnerable to predators, and F_*i*_ = F_*g*_ indicates that the Coati in the middle of the group is aware of the danger and needs to stay close to other Coatis to avoid predation.

### Pseudocode and flowchart

**Fig. 5:**
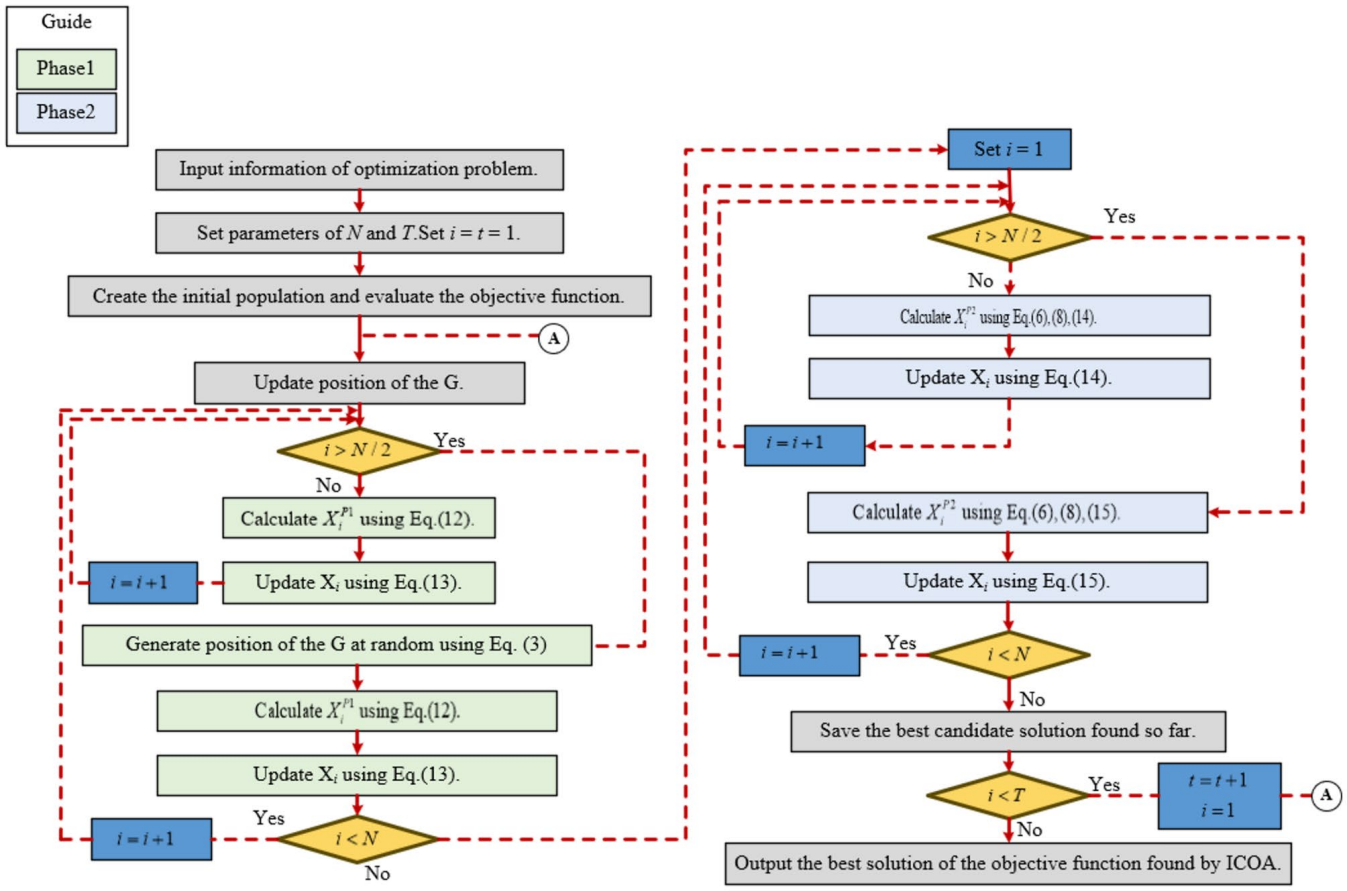
Optimization flowchart of the TNTWCOA algorithm.

The flowchart of the proposed TNTWCOA technique is shown in Fig. 5. Different improvement strategies are proposed in the initialization process, exploration phase and exploitation phase. In initialization process. The algorithm introduces chaotic sequence mechanism to initialize the position. The position distribution of the initial solution is more uniform, the high quality initial solution is generated, the population richness is increased, and the problem of poor quality and uneven initial solution of the Coati Optimization Algorithm is solved. In exploration phase, the nonlinear inertial weight factor is introduced to coordinate the local optimization ability and global search ability of the algorithm. In the exploitation phase, adaptive T-distribution variation is introduced to increase the diversity of individual population under low fitness value and improve the ability of the algorithm to jump out of the local optimal value. At the same time, the alert update mechanism is proposed to improve the alert ability of COA algorithm, so that it can search within the optional range. When Coati is aware of the danger, Coati on the edge of the population will quickly move to the safe area to obtain a better position, while Coati in the middle of the population will randomly move to get closer to other Coatis. Furthermore, Algorithm 1 defines the TNTWCOA technique’s pseudocode.

**Algorithm 1:**
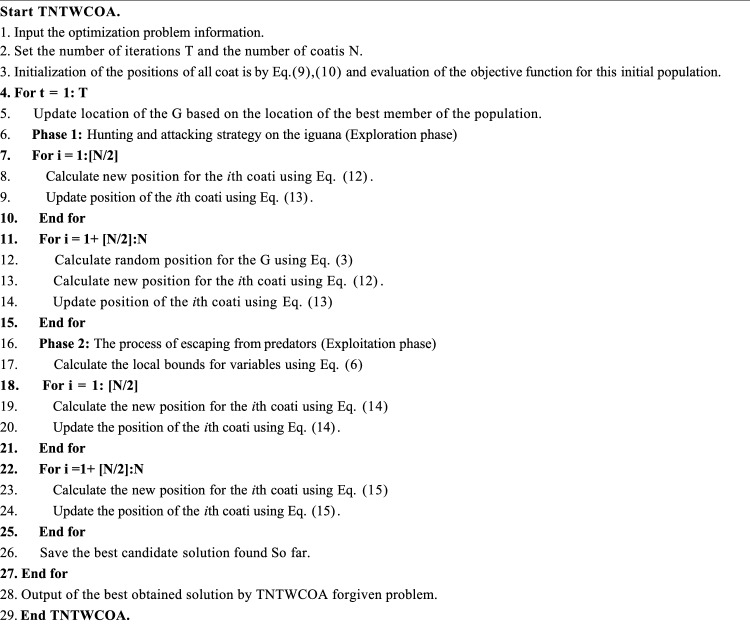
Pseudocode of the TNTWCOA algorithm.

### Computational complexity

Assume that the number of iterations is T, the number of populations is N, and the dimension of the problem is M. According to the algorithm flow, the computational complexity of the ICOA algorithm to initialize the population and compute the initial value of the objective function is $O(N \times M)$; In the Exploration phase, the computational complexity of updating the population is $O(N/2 \times M \times T)$, respectively. $O(N/2 \times M \times T)$, the computational complexity of Calculate random position for the G is $O(N/2 \times M \times T)$. In the Exploitation phase, the computational complexity required to update the raccoon's position is $O(N/2 \times M \times T)$ and $O(N/2 \times M \times T)$, respectively. Therefore, the comprehensive complexity of the ICOA algorithm proposed in this paper is $O(N \times M) + 5 \times O(N/2 \times M \times T) = O(N \times M(1 + 5 \times N/2))$. According to the references, the complexity of the original COA algorithm is $O(N \times M(1 + 5 \times N/2))$, so the improved algorithm does not increase the computational complexity of the algorithm.

## Experimental studies and results

To verify the effectiveness of the Improved Coati Optimization Algorithm with Multiple strategies proposed in this paper. In this section, the well-known IEEE CEC2017 benchmark functions are used to validity of the Improved Coati Optimization Algorithm with Multiple strategies proposed in this paper in 30, 50 and 100 dimensions and compared with the Improved Coati Optimization Algorithm (ICOA), Coati Optimization Algorithm (COA), Golden Jackal Optimization Algorithm (GJO), Osprey Optimization Algorithm (OOA), Sand Cat Swarm Optimization Algorithm (SCSO), Subtraction-Average-Based Optimizer (SABO). The evaluation involves using statistical measurements, such as best values, mean values, worst values, and standard deviation (STD). All statistical measurements were obtained after 30 runs of each algorithm. The number of iterations was 10,000 and the individual was 50. The analyzes and discusses the optimization of IEEE CEC2017 benchmark functions between the algorithm proposed in this paper and other algorithms in the case of 30, 50 and 100 dimensions respectively as followings. The details of CEC2017 was shown in Table 1.

**Table 1:** Test functions details of CEC2017.

Name	No. functions	F_min_
Unimodal functions	CEC 1 Rotated high conditioned elliptic function	100
	CEC 2 Rotated bent cigar function	200
Simple multimodal functions	CEC 3 Rotated discus function	300
	CEC 4 Shifted and rotated Rosenbrock’s function	400
Hybrid Functions	CEC 5 Shifted and rotated Ackley’s function	500
	CEC 6 Shifted and rotated Weierstrass function	600
	CEC 7 Shifted and rotated Griewank’s function	700
	CEC 8 Shifted Rastrigin’s function	800
	CEC 9 Shifted and rotated Rastrigin’s function	900
	CEC 10 Shifted Schwefel’s function	1000
	CEC 11 Shifted and rotated Schwefel’s function	1100
	CEC 12 Shifted and rotated Katsuura function	1200
	CEC 13 Shifted and rotated HappyCat function	1300
	CEC 14 Shifted and rotated HGBat function	1400
	CEC 15 Shifted and rotated expanded Griewank’s plus Rosenbrock’s function	1500
	CEC 16 Shifted and rotated expanded Scaffer’s F6 function	1600
Composition functions	CEC 17 Hybrid function 1 (N = 3)	
	CEC 18 Hybrid function 2 (N = 3)	1700
	CEC 19 Hybrid function 3 (N = 4)	1800
	CEC 20 Hybrid function 4 (N = 4)	1900
	CEC 21 Hybrid function 5 (N = 5)	2000
	CEC 22 Hybrid function 6 (N = 5)	2100
	CEC 23 Composition function 1 (N = 5)	2200
	CEC 24 Composition function 2 (N = 3)	2300
	CEC 25 Composition function 3 (N = 3)	2400
	CEC 26 Composition function 4 (N = 5)	2500
	CEC 27 Composition Function 5 (N = 5)	2600
	CEC 28 Composition function 6 (N = 5)	2700
	CEC 29 Composition function 7 (N = 3)	2800
	CEC 30 Composition function 8 (N = 3)	2900
	Search range: [− 100 100]^dim^	3000

### Experiments on IEEE CEC2017 benchmark functions (Dim = 30)

#### Statistics analysis

Table 2 shows the statistical results of IEEE CEC2017 benchmark functions in 30 dimensions optimized by TNTWCOA ICOA, COA, GJO, OOA, SCSO and SABO algorithms. Also the Friedman values based on the average value of the IEEE CEC2017 benchmark functions optimized by the TNTWCOA, ICOA, COA, GJO, OOA, SCSO and SABO algorithms were statistically analyzed. As can be seen from Table 2, When optimizing the other 28 test functions in the 30-dimensional case, More or less evaluation index of the TNTWCOA proposed in this paper is superior to that of the ICOA, GJO, COA, SCSO, OOA, and SABO algorithms; Among them, when F1, F3, F4, F11, F12, F13, F14, F15, F18, F19, F22, F25, F28 and F30 functions are optimized, all the evaluation indexes of TNTWCOA algorithm are optimal, showing excellent performance; the performance of std value is inferior to that of SABO algorithm for F5, that of GJO algorithm for F6, that of ICOA algorithm for F9 and F20, the performance of std and worse value are inferior to that of SABO algorithm for F7, the performance of min value is inferior to that of SCSO algorithm and std value is inferior to COA algorithm for F8, the performance of min value is inferior to that of SBSO algorithm, avg, median and worse value are inferior to COA algorithm for F10, the performance of std and worse value are inferior to that of ICOA algorithm for F16, F17, the performance of std and worse value are inferior to that of COA algorithm for F21. All values are inferior to that of ICOA algorithm for F23, Std, avg, median and worse inferior to that of COA algorithm for F24, The std and worse values when optimizing F26 are not as good as GJO algorithm, The std and worse values when optimizing F27 and F29 are not as good as ICOA algorithm.

**Table 2:** The statistical results of benchmark functions using the proposed technique and other five algorithms (Dim = 30).

Function	Item	TNTWCOA	ICOA	COA	GJO	OOA	SCSO	SABO
F1	Min	**1.16595E+02**	3.84951E+08	2.77953E+10	2.52369E+09	3.06896E+10	1.15679E+08	1.42340E+09
	Std	**7.86979E+03**	1.18564E+09	9.32668E+09	2.84646E+09	9.08098E+09	1.52179E+09	1.58340E+09
	Avg	**9.69399E+03**	1.36209E+09	5.15538E+10	8.98899E+09	5.34793E+10	2.08479E+09	3.66115E+09
	Median	**5.57403E+03**	9.29104E+08	5.32515E+10	8.47447E+09	5.46289E+10	1.77217E+09	3.23600E+09
	Worse	**2.01314E+04**	5.23321E+09	6.86305E+10	1.51705E+10	6.57945E+10	6.14432E+09	6.95404E+09
F3	Min	**3.00000E+02**	2.56189E+04	6.46788E+04	2.58088E+04	7.33478E+04	1.64870E+04	1.82102E+04
	Std	**4.26947E−02**	9.66597E+03	6.65800E+03	9.53719E+03	5.24376E+03	1.06829E+04	6.95020E+03
	Avg	**3.00011E+02**	4.86105E+04	7.90179E+04	4.34125E+04	8.77482E+04	3.31692E+04	3.09576E+04
	Median	**3.00000E+02**	4.93966E+04	7.93855E+04	4.35344E+04	8.92561E+04	3.02468E+04	3.10121E+04
	Worse	**3.00192E+02**	6.85805E+04	9.03436E+04	6.30538E+04	9.42289E+04	5.40793E+04	4.36327E+04
F4	Min	**4.00004E+02**	5.88916E+02	6.41532E+03	5.92872E+02	9.37096E+03	4.85034E+02	6.38397E+02
	Std	**4.45687E+01**	4.12813E+02	3.28541E+03	6.11040E+02	2.53751E+03	9.67028E+01	2.61340E+02
	Avg	**4.54418E+02**	9.96337E+02	1.41032E+04	1.03278E+03	1.32283E+04	6.08220E+02	1.02017E+03
	Median	**4.64117E+02**	8.14062E+02	1.37277E+04	8.66552E+02	1.22771E+04	5.76071E+02	9.82368E+02
	Worse	**5.21465E+02**	2.15035E+03	1.97393E+04	3.85828E+03	1.83911E+04	9.13004E+02	1.61598E+03
F5	Min	**5.77607E+02**	6.83020E+02	7.42628E+02	6.31153E+02	7.96335E+02	6.47610E+02	6.80849E+02
	Std	3.82982E+01	2.83847E+01	4.30637E+01	3.68021E+01	3.48423E+01	4.51804E+01	**2.42772E+01**
	Avg	**6.51056E+02**	7.57038E+02	9.06924E+02	6.85926E+02	9.01740E+02	7.22865E+02	7.30388E+02
	Median	**6.50238E+02**	7.57686E+02	9.11346E+02	6.81920E+02	9.08719E+02	7.22565E+02	7.31477E+02
	Worse	**7.18890E+02**	8.05995E+02	9.68563E+02	7.59204E+02	9.68172E+02	8.22157E+02	7.75396E+02
F6	Min	**6.07981E+02**	6.13622E+02	6.70453E+02	6.13478E+02	6.68221E+02	6.38504E+02	6.24211E+02
	Std	9.04564E+00	1.25456E+01	7.45750E+00	**7.34322E+00**	7.88464E+00	9.58931E+00	1.14141E+01
	Avg	**6.25201E+02**	6.57284E+02	6.85569E+02	6.28367E+02	6.83757E+02	6.58268E+02	6.42350E+02
	Median	**6.26257E+02**	6.59521E+02	6.86955E+02	6.28390E+02	6.83135E+02	6.61196E+02	6.41244E+02
	Worse	**6.41021E+02**	6.70824E+02	6.97298E+02	6.44517E+02	6.97355E+02	6.71713E+02	6.75987E+02
F7	Min	**8.42379E+02**	1.01784E+03	1.25789E+03	9.00544E+02	1.25008E+03	9.05108E+02	9.66776E+02
	Std	6.28840E+01	8.18340E+01	4.47864E+01	4.00658E+01	5.88657E+01	8.87798E+01	**3.09438E+01**
	Avg	**9.41228E+02**	1.18175E+03	1.40333E+03	9.99996E+02	1.38172E+03	1.05276E+03	1.01808E+03
	Median	**9.34367E+02**	1.19814E+03	1.40463E+03	9.96615E+02	1.40123E+03	1.05074E+03	1.01760E+03
	Worse	1.13125E+03	1.31798E+03	1.49925E+03	1.08813E+03	1.46573E+03	1.25067E+03	**1.08021E+03**
F8	Min	9.06460E+02	9.48313E+02	1.07728E+03	8.98938E+02	1.03480E+03	**8.94082E+02**	9.73446E+02
	Std	2.27363E+01	1.85745E+01	**2.20926E+01**	2.71735E+01	2.86541E+01	3.12598E+01	3.08385E+01
	Avg	**9.44232E+02**	9.90754E+02	1.12769E+03	9.53357E+02	1.11190E+03	9.89001E+02	1.02954E+03
	Median	**9.47229E+02**	9.91483E+02	1.12664E+03	9.53886E+02	1.11328E+03	9.88728E+02	1.03199E+03
	Worse	**9.93021E+02**	1.03094E+03	1.16892E+03	1.01369E+03	1.16684E+03	1.05697E+03	1.13656E+03
F9	Min	**1.25734E+03**	4.28716E+03	7.20392E+03	2.30182E+03	5.73481E+03	3.41688E+03	1.89177E+03
	Std	1.13895E+03	**5.90625E+02**	1.16924E+03	1.14061E+03	1.64813E+03	7.23803E+02	8.70722E+02
	Avg	**3.12629E+03**	5.96983E+03	9.80777E+03	3.69981E+03	9.62407E+03	5.17692E+03	3.22955E+03
	Median	**2.97176E+03**	6.03142E+03	9.69866E+03	3.40863E+03	9.83952E+03	5.17552E+03	3.02244E+03
	Worse	**5.24115E+03**	6.98038E+03	1.14661E+04	7.17812E+03	1.29960E+04	6.75841E+03	5.00180E+03
F10	Min	**3.53086E+03**	4.80969E+03	7.73614E+03	4.23637E+03	7.13293E+03	4.47879E+03	7.55832E+03
	Std	1.27284E+03	5.28466E+02	4.23474E+02	1.01090E+03	4.39904E+02	6.12583E+02	**3.17327E+02**
	Avg	6.27592E+03	**5.83810E+03**	8.69659E+03	5.86178E+03	8.41366E+03	**5.81833E+03**	8.29190E+03
	Median	6.42202E+03	**5.88153E+03**	8.83093E+03	5.59720E+03	8.47669E+03	**5.83906E+03**	8.28757E+03
	Worse	8.17189E+03	**6.85535E+03**	**9.27894E+03**	8.29281E+03	9.14455E+03	**6.93121E+03**	8.87012E+03
F11	Min	**1.17944E+03**	1.27558E+03	3.86317E+03	1.44332E+03	4.52802E+03	1.26782E+03	1.51061E+03
	Std	**9.95684E+01**	1.90355E+02	1.94773E+03	1.13082E+03	2.11433E+03	2.33287E+02	6.48463E+02
	Avg	**1.31258E+03**	1.55652E+03	8.18875E+03	2.77987E+03	8.05355E+03	1.50894E+03	2.41302E+03
	Median	**1.29126E+03**	1.54314E+03	8.15388E+03	2.64303E+03	8.35191E+03	1.42225E+03	2.24487E+03
	Worse	**1.65604E+03**	2.18909E+03	1.27098E+04	4.98204E+03	1.17794E+04	2.29456E+03	4.69500E+03
F12	Min	**5.56400E+04**	2.11851E+07	6.61620E+09	1.30845E+07	7.16070E+09	1.59250E+06	3.73155E+07
	Std	**2.19906E+07**	5.59258E+08	2.68979E+09	6.86231E+08	2.91518E+09	8.40084E+07	1.18679E+08
	Avg	**4.30655E+06**	3.59938E+08	1.26796E+10	6.69974E+08	1.18897E+10	5.92169E+07	1.46451E+08
	Median	**1.75886E+05**	1.23863E+08	1.32113E+10	3.69674E+08	1.17802E+10	2.87030E+07	9.96360E+07
	Worse	**1.20728E+08**	2.30034E+09	1.62070E+10	2.32688E+09	1.67710E+10	3.39514E+08	5.51839E+08
F13	Min	**4.08276E+03**	1.02128E+07	1.27855E+09	2.38342E+04	1.71178E+09	2.01058E+04	1.41643E+05
	Std	**2.03677E+04**	4.58410E+08	3.93105E+09	1.04584E+09	3.21388E+09	3.31469E+07	2.43653E+05
	Avg	**1.82722E+04**	3.81834E+08	8.22762E+09	3.57559E+08	6.11148E+09	1.05901E+07	3.87404E+05
	Median	**1.03512E+04**	1.83269E+08	7.49706E+09	8.67159E+06	5.58093E+09	8.88199E+04	2.98966E+05
	Worse	**6.83398E+04**	1.76385E+09	1.56035E+10	4.21567E+09	1.26602E+10	1.39443E+08	1.26744E+06
F14	Min	**1.91955E+03**	2.37330E+03	2.56772E+05	5.63826E+03	2.83785E+05	2.59784E+03	2.02976E+04
	Std	**7.42221E+03**	7.00590E+04	1.81795E+06	2.34371E+05	2.01314E+06	3.25627E+04	3.45356E+05
	Avg	**9.17738E+03**	3.96371E+04	2.38062E+06	1.73401E+05	2.62742E+06	4.41539E+04	2.51770E+05
	Median	**7.09814E+03**	1.09527E+04	1.79527E+06	7.29224E+04	1.79783E+06	4.51426E+04	9.35016E+04
	Worse	**3.46775E+04**	3.17109E+05	7.69851E+06	9.14840E+05	7.75712E+06	1.04973E+05	1.51283E+06
F15	Min	**1.88276E+03**	1.50508E+04	9.87681E+07	1.05626E+04	1.02686E+07	9.44674E+03	1.53320E+04
	Std	**5.34746E+03**	1.69903E+08	3.02412E+08	1.21758E+06	3.03351E+08	6.27466E+06	1.20982E+05
	Avg	**5.57033E+03**	7.70892E+07	4.02010E+08	9.14633E+05	3.82374E+08	1.30255E+06	1.10234E+05
	Median	**4.18136E+03**	1.06513E+07	3.49648E+08	6.76644E+04	3.05996E+08	6.47777E+04	4.71382E+04
	Worse	**2.72066E+04**	7.30672E+08	1.26297E+09	3.81272E+06	1.24164E+09	3.44614E+07	4.83986E+05
F16	Min	**2.04546E+03**	2.47029E+03	4.39427E+03	2.17092E+03	3.79167E+03	2.36283E+03	2.78449E+03
	Std	2.86431E+02	**1.33806E+02**	6.75504E+02	3.82484E+02	7.35864E+02	2.80576E+02	3.22025E+02
	Avg	**2.68110E+03**	2.76933E+03	5.80541E+03	2.73740E+03	4.91930E+03	2.83489E+03	3.80189E+03
	Median	**2.70573E+03**	2.76917E+03	5.72542E+03	2.63418E+03	4.72393E+03	2.84720E+03	3.88238E+03
	Worse	3.15456E+03	**3.10250E+03**	7.04534E+03	4.12174E+03	6.85330E+03	3.52948E+03	4.27515E+03
F17	Min	**1.76840E+03**	1.88013E+03	2.83179E+03	1.82312E+03	2.55291E+03	1.94131E+03	2.26477E+03
	Std	2.26740E+02	**1.14839E+02**	1.46219E+03	2.05817E+02	1.45683E+03	1.76053E+02	2.68118E+02
	Avg	**2.10403E+03**	2.10859E+03	4.01471E+03	2.13086E+03	3.77639E+03	2.25559E+03	2.85689E+03
	Median	**2.10036E+03**	2.10178E+03	3.58459E+03	2.15049E+03	3.54030E+03	2.27264E+03	2.91086E+03
	Worse	2.48753E+03	**2.42884E+03**	9.03670E+03	2.54481E+03	1.04252E+04	2.59274E+03	3.25516E+03
F18	Min	**7.22362E+03**	7.14153E+04	2.46598E+06	3.30334E+04	4.71047E+06	4.04501E+04	6.02145E+04
	Std	**7.45602E+04**	2.65204E+05	2.86946E+07	1.87155E+06	2.95348E+07	7.88673E+05	9.34765E+05
	Avg	**1.03750E+05**	3.92911E+05	3.28097E+07	1.28056E+06	3.32930E+07	6.65316E+05	9.48450E+05
	Median	**8.13198E+04**	3.32249E+05	2.33954E+07	6.48138E+05	2.59296E+07	4.84750E+05	7.21938E+05
	Worse	**2.88785E+05**	1.05658E+06	1.09416E+08	7.71790E+06	1.40829E+08	3.70886E+06	3.38905E+06
F19	Min	**2.00222E+03**	1.43117E+05	2.14247E+07	2.85006E+03	1.77974E+07	1.19726E+04	2.50219E+04
	Std	**3.61857E+03**	9.59692E+07	2.78453E+08	2.49765E+07	4.10652E+08	7.36891E+05	7.44349E+05
	Avg	**6.72828E+03**	6.95528E+07	4.16128E+08	7.57904E+06	4.18547E+08	6.23572E+05	7.15717E+05
	Median	**6.08135E+03**	2.13172E+07	4.22915E+08	1.23974E+06	2.69752E+08	3.54746E+05	5.05876E+05
	Worse	**1.56382E+04**	3.12139E+08	9.08275E+08	1.36016E+08	1.69584E+09	3.24579E+06	3.57089E+06
F20	Min	**2.21279E+03**	2.31883E+03	2.59542E+03	2.22702E+03	2.45555E+03	2.28128E+03	2.52645E+03
	Std	9.87647E+01	**8.05317E+01**	1.74670E+02	1.60469E+02	2.04380E+02	1.49733E+02	1.41752E+02
	Avg	**2.36932E+03**	2.48578E+03	2.93842E+03	2.45566E+03	2.88857E+03	2.59372E+03	2.95999E+03
	Median	**2.36743E+03**	2.47684E+03	2.91690E+03	2.43361E+03	2.92790E+03	2.58475E+03	2.96317E+03
	Worse	**2.60575E+03**	2.66227E+03	3.22312E+03	2.80278E+03	3.22531E+03	2.91240E+03	3.15799E+03
F21	Min	2.38464E+03	2.27354E+03	**2.65476E+03**	2.39533E+03	2.61877E+03	2.41026E+03	2.47902E+03
	Std	2.67606E+01	4.96313E+01	**4.20216E+01**	2.42021E+01	3.95049E+01	4.27296E+01	2.94574E+01
	Avg	**2.42970E+03**	2.43022E+03	2.73869E+03	2.44728E+03	2.70504E+03	2.48794E+03	2.54419E+03
	Median	**2.42252E+03**	2.44188E+03	2.73640E+03	2.44714E+03	2.70188E+03	2.48589E+03	2.53845E+03
	Worse	**2.48289E+03**	2.48506E+03	2.82056E+03	2.50108E+03	2.77842E+03	2.59618E+03	2.59386E+03
F22	Min	**2.30000E+03**	2.42617E+03	6.93128E+03	2.73127E+03	6.56693E+03	2.36227E+03	2.56280E+03
	Std	**7.10151E+01**	1.43620E+03	8.66216E+02	1.60448E+03	8.57168E+02	1.62275E+03	7.64337E+02
	Avg	**2.31425E+03**	3.35245E+03	9.12448E+03	4.18682E+03	9.11697E+03	3.37130E+03	3.19442E+03
	Median	**2.30000E+03**	2.72855E+03	9.27400E+03	3.60124E+03	9.30584E+03	2.64949E+03	2.94715E+03
	Worse	**2.69001E+03**	7.42284E+03	1.05585E+04	1.00989E+04	1.04557E+04	7.44345E+03	6.33265E+03
F23	Min	2.76189E+03	**2.75564E+03**	3.33606E+03	2.77133E+03	3.23951E+03	2.78429E+03	2.95754E+03
	Std	3.64945E+01	**2.17696E+01**	1.11349E+02	4.81464E+01	1.42649E+02	5.46662E+01	6.94977E+01
	Avg	2.83708E+03	**2.78940E+03**	3.55991E+03	2.85373E+03	3.60785E+03	2.87760E+03	3.04787E+03
	Median	2.83552E+03	**2.79046E+03**	3.56302E+03	2.84026E+03	3.62067E+03	2.87549E+03	3.02972E+03
	Worse	2.89171E+03	**2.82479E+03**	3.77968E+03	2.99505E+03	3.81537E+03	2.99059E+03	3.24176E+03
F24	Min	**2.90650E+03**	2.92406E+03	3.44905E+03	2.92226E+03	3.47330E+03	2.95246E+03	3.05803E+03
	Std	4.56328E+01	**1.68263E+01**	1.41243E+02	5.58798E+01	2.62848E+02	5.88942E+01	7.32878E+01
	Avg	2.99594E+03	**2.95242E+03**	3.74454E+03	3.04049E+03	4.01939E+03	3.06133E+03	3.15227E+03
	Median	2.99157E+03	**2.94742E+03**	3.73075E+03	3.04209E+03	4.07071E+03	3.04831E+03	3.12756E+03
	Worse	3.08516E+03	**2.99832E+03**	4.02836E+03	3.13070E+03	4.55651E+03	3.21726E+03	3.31470E+03
F25	Min	**2.88352E+03**	3.01320E+03	3.86638E+03	2.97031E+03	4.14648E+03	2.89071E+03	3.02978E+03
	Std	**1.29506E+01**	3.29755E+01	5.06323E+02	1.03027E+02	4.06548E+02	5.17784E+01	7.51641E+01
	Avg	**2.89299E+03**	3.07249E+03	4.97486E+03	3.11406E+03	4.91325E+03	3.01770E+03	3.12655E+03
	Median	**2.88851E+03**	3.06984E+03	4.99704E+03	3.10798E+03	5.00656E+03	3.00786E+03	3.11423E+03
	Worse	**2.94368E+03**	3.13951E+03	5.80214E+03	3.36721E+03	6.09149E+03	3.16236E+03	3.35426E+03
F26	Min	**2.80000E+03**	3.41332E+03	8.90986E+03	4.89326E+03	8.91475E+03	3.22984E+03	4.38459E+03
	Std	1.24320E+03	1.54969E+03	8.62175E+02	**5.64219E+02**	1.02753E+03	1.21286E+03	1.38814E+03
	Avg	**5.00187E+03**	5.17051E+03	1.10131E+04	5.55267E+03	1.05831E+04	5.92864E+03	7.41319E+03
	Median	**5.30662E+03**	4.54951E+03	1.11634E+04	5.43896E+03	1.04224E+04	6.06286E+03	7.74431E+03
	Worse	7.36060E+03	7.91050E+03	1.24423E+04	**7.34009E+03**	1.31339E+04	8.81963E+03	9.13146E+03
F27	Min	**3.21930E+03**	3.24540E+03	3.78150E+03	3.24638E+03	4.00329E+03	3.23089E+03	3.23768E+03
	Std	3.34974E+01	**1.90923E+01**	3.37578E+02	3.62076E+01	4.15978E+02	8.83139E+01	6.92942E+01
	Avg	**3.26594E+03**	3.27717E+03	4.35445E+03	3.30284E+03	4.68805E+03	3.36603E+03	3.32915E+03
	Median	**3.26091E+03**	3.27045E+03	4.29461E+03	3.29807E+03	4.62897E+03	3.36032E+03	3.31870E+03
	Worse	3.37544E+03	**3.33062E+03**	5.53006E+03	3.38827E+03	5.48189E+03	3.61308E+03	3.58799E+03
F28	Min	**3.10000E+03**	3.39591E+03	5.80458E+03	3.49242E+03	5.65725E+03	3.26392E+03	3.47871E+03
	Std	**7.50152E+01**	1.25855E+02	5.71490E+02	2.52912E+02	7.75535E+02	1.12022E+02	2.33276E+02
	Avg	**3.17855E+03**	3.53832E+03	7.22545E+03	3.72880E+03	6.93878E+03	3.39955E+03	3.79642E+03
	Median	**3.20350E+03**	3.48970E+03	7.18733E+03	3.65095E+03	6.79393E+03	3.36738E+03	3.71845E+03
	Worse	**3.34904E+03**	3.91086E+03	8.29759E+03	4.68120E+03	8.71404E+03	3.83756E+03	4.41036E+03
F29	Min	**3.52054E+03**	3.69029E+03	5.61990E+03	3.64003E+03	5.26305E+03	3.80784E+03	4.56620E+03
	Std	2.37749E+02	**1.66652E+0**2	9.32457E+02	2.19654E+02	8.98675E+02	3.11245E+02	3.97585E+02
	Avg	**3.86277E+03**	4.08934E+03	7.31521E+03	4.02507E+03	6.80400E+03	4.39521E+03	5.15953E+03
	Median	**3.86695E+03**	4.09679E+03	7.28810E+03	4.00306E+03	6.85981E+03	4.39556E+03	5.21391E+03
	Worse	4.44162E+03	**4.37577E+03**	9.20253E+03	4.49470E+03	9.39636E+03	4.97102E+03	6.27878E+03
F30	Min	**5.71972E+03**	1.62959E+05	3.53215E+07	1.89870E+06	1.90258E+08	2.99658E+05	1.48478E+06
	Std	**1.20870E+05**	1.70521E+07	8.81721E+08	9.83723E+06	7.69670E+08	3.54864E+06	7.01364E+06
	Avg	**4.13449E+04**	1.51538E+07	1.26345E+09	1.36271E+07	1.24594E+09	4.56867E+06	1.06890E+07
	Median	**1.19741E+04**	6.85055E+06	1.01625E+09	1.02673E+07	1.25659E+09	3.52318E+06	9.02838E+06
	Worse	**6.61812E+05**	5.44215E+07	3.59733E+09	3.64864E+07	2.99994E+09	1.33857E+07	3.09151E+07
Friedman	Value	1.17241E+00	3.24138E+00	6.68966E+00	3.41379E+00	6.24138E+00	3.24138E+00	4.00000E+00
	Rank	**1**	**2**	**6**	**3**	**5**	**2**	**4**

Significant values are in bold.

In general, the improved algorithm shows stronger optimization ability in the case of 30 dimensions, especially for F3, F4, F6, F11, F17 functions, whose optimization value is close to the theoretical value of the function. Friedman's overall order is TNTWCOA > ICOA > GJO > SABO > OOA > SCSO > COA. Therefore, from the statistical results of evaluation index, when Dim = 30, the algorithm proposed in this paper shows excellent performance compared with other six algorithms. Compared with the original COA algorithm, the statistical results of evaluation index of the improved algorithm have been significantly improved.

#### Convergence analysis

**Fig. 6:**
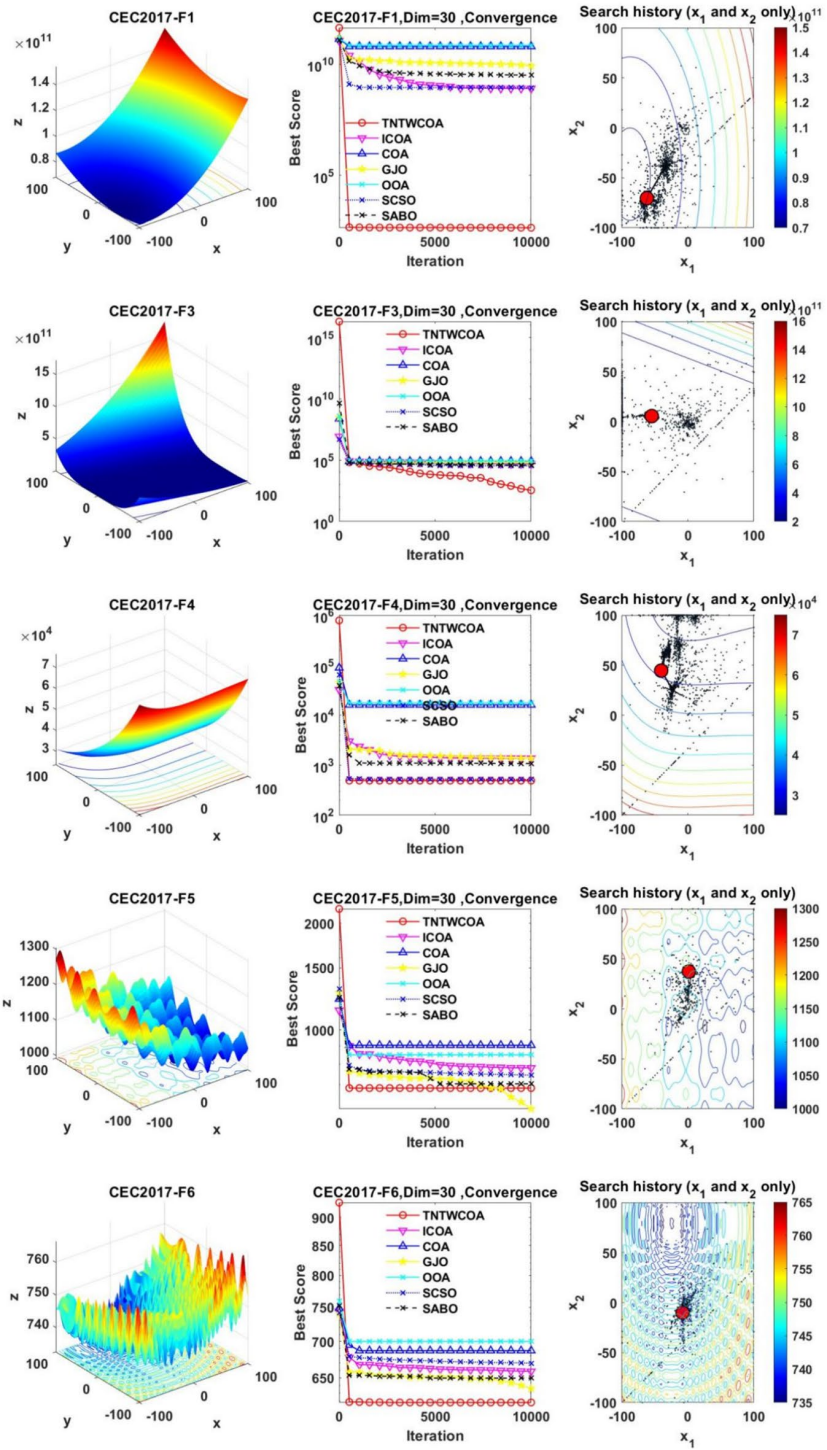

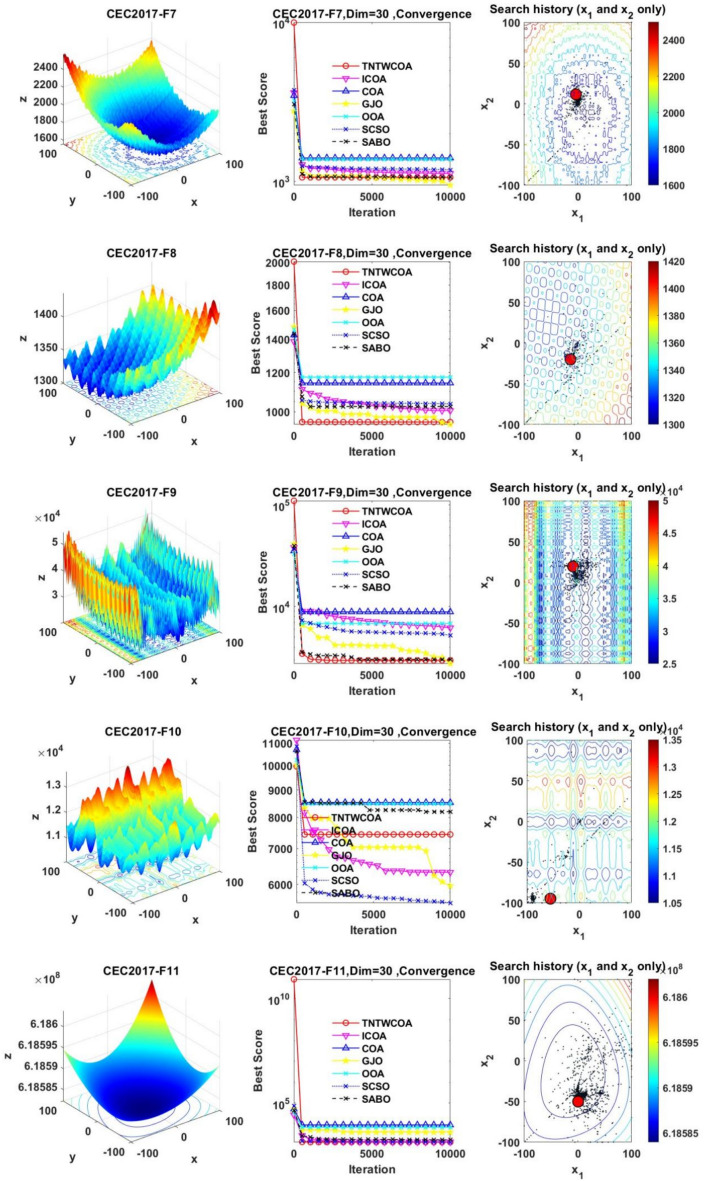

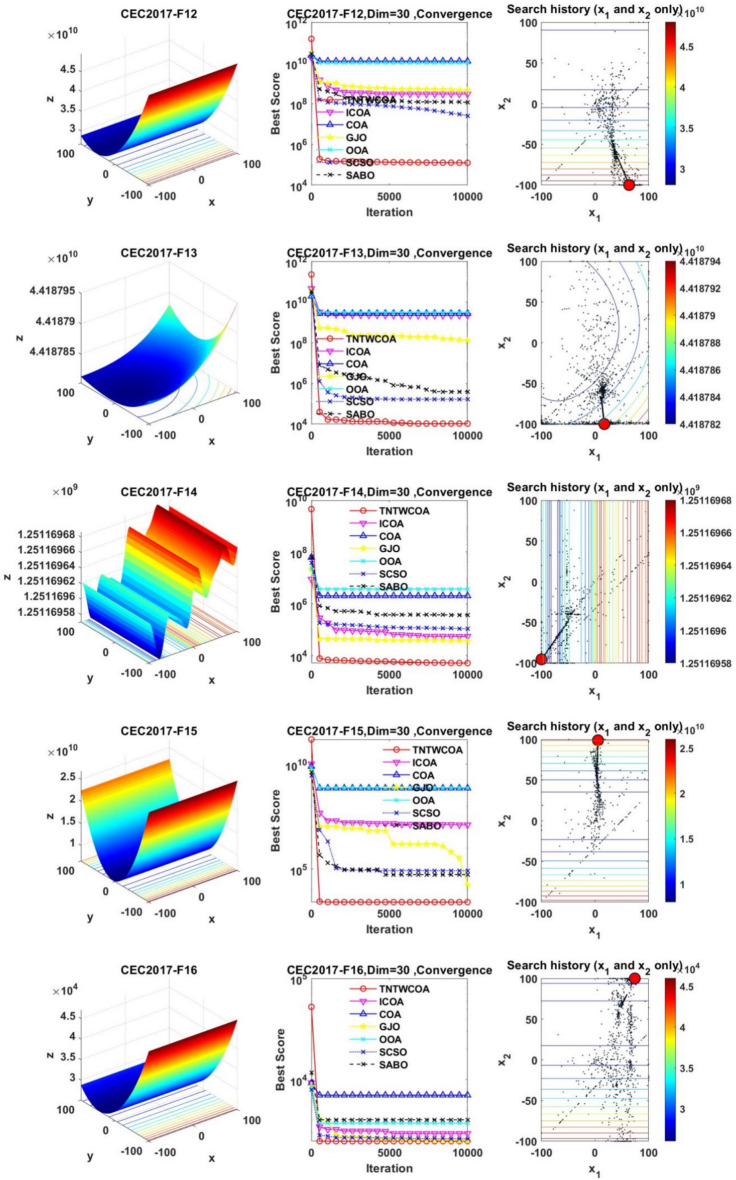

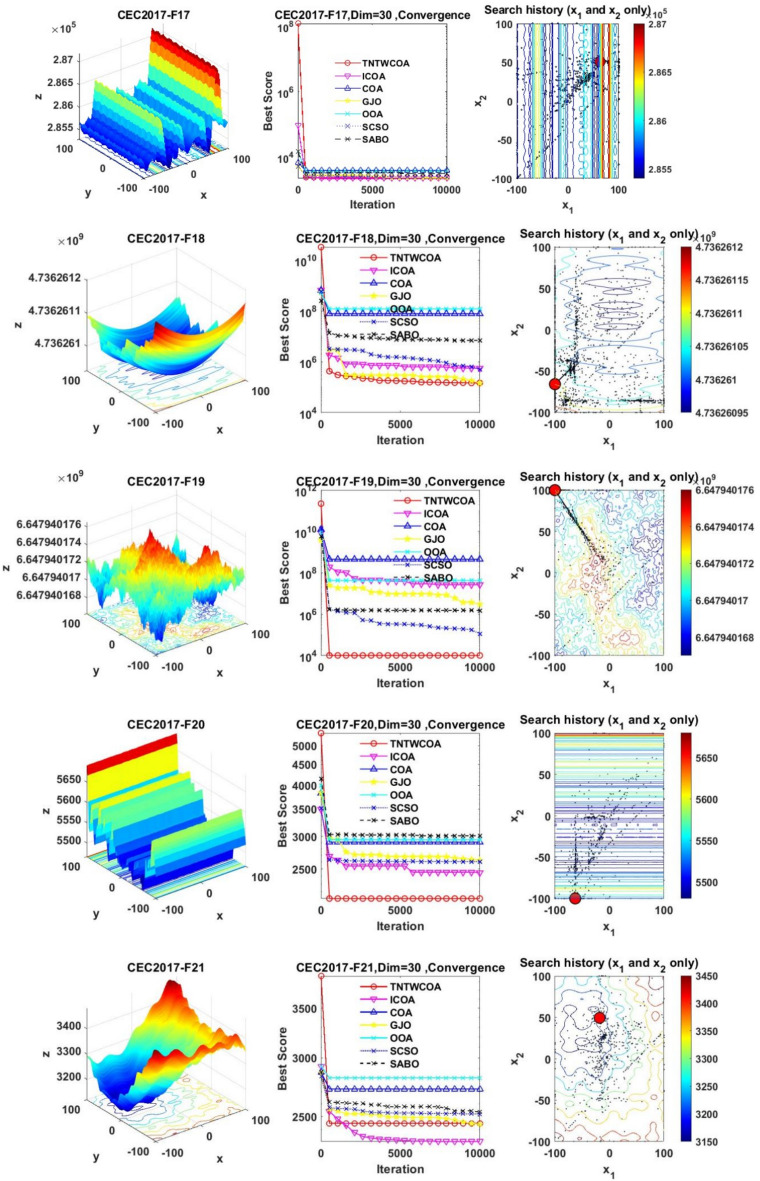

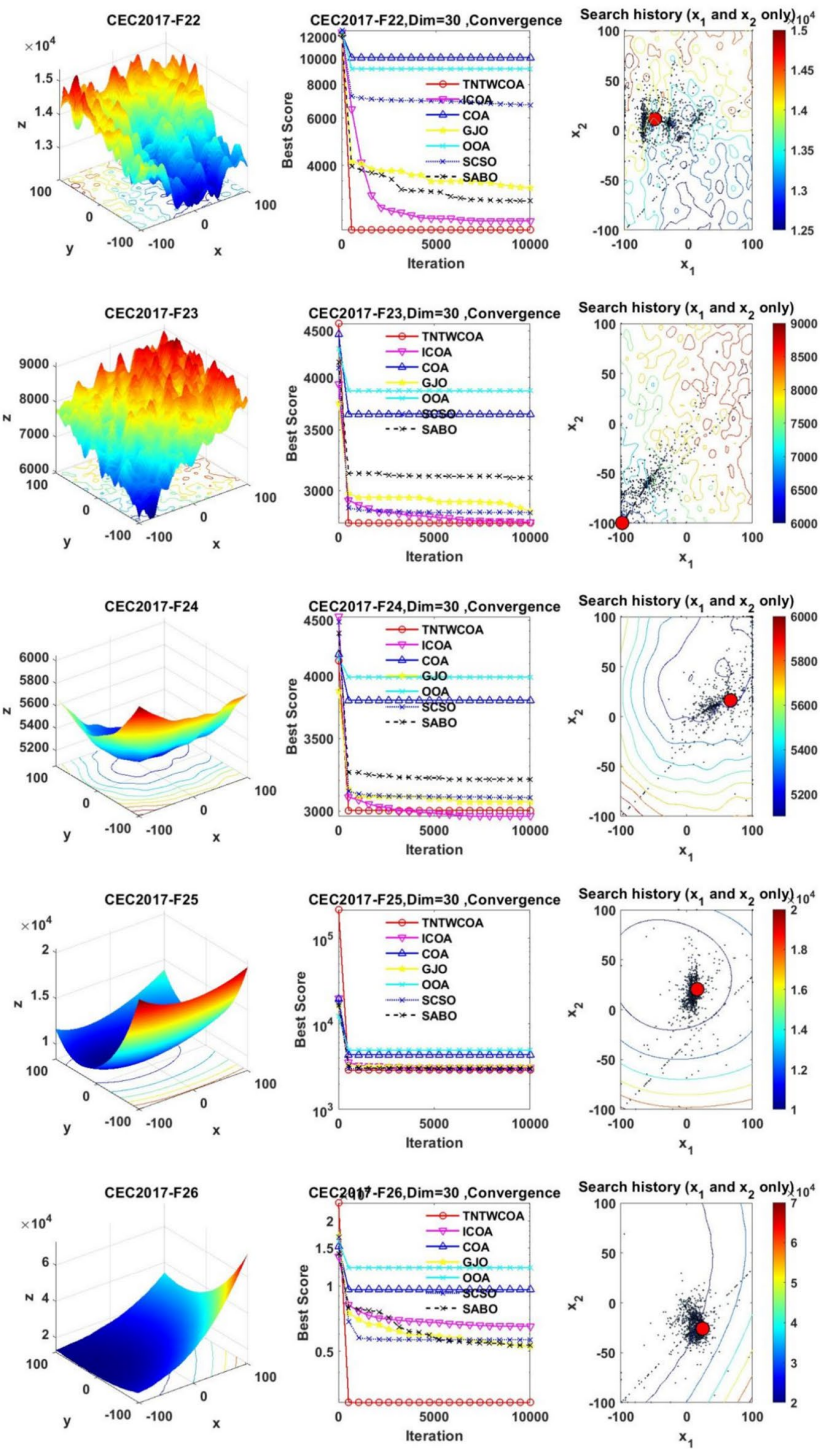

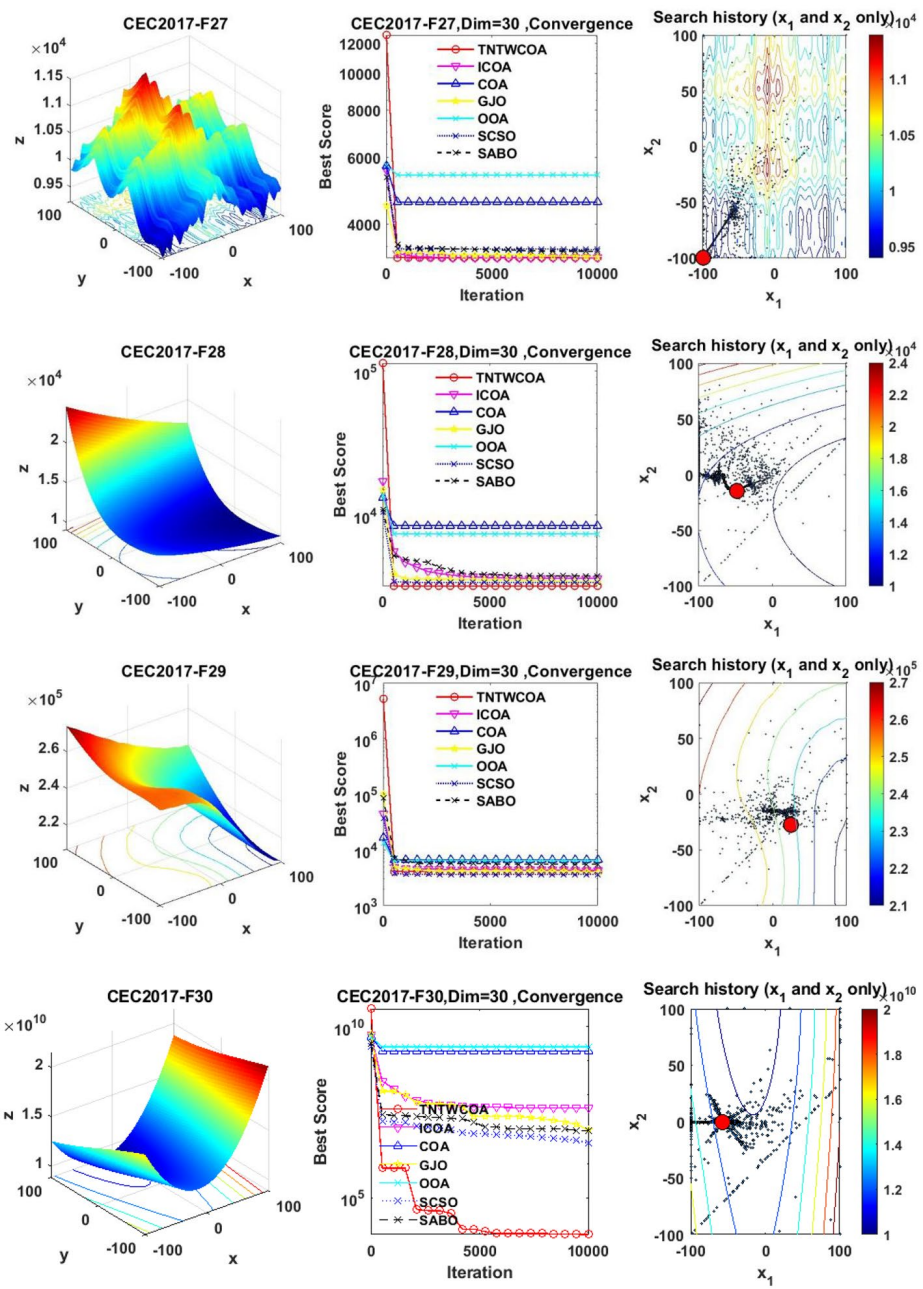
The convergence curves and search history of the proposed technique and other five algorithms for IEEE CEC2017 benchmark functions (Dim = 30).

Figure 6 illustrates the convergence curves of GJO, SCSO, OOA, SABO, original COA, ICOA and TNTWCOA on 29 benchmark functions of the IEEE CEC2017 throughout the iterations with 10000times.

As can be seen from Fig. 6, except for functions F10 and F29, the convergence speed of the improved TNTWCOA algorithm is optimal. Among them, when the function F1, F3, F4, F6, F11, F12, F13, F14, F15, F16, F17, F18, F19, F20, F22, F25, F26, F27, F28 and F30 are optimized, it can quickly converge to the best value and be stable. When optimizing functions F5, F7, F8, F9, F21, they can rapidly converge to the best value and remain stable, but the best value will be surpassed by other algorithms in the later period, for example, when optimizing F5, F7, F8, F9, it will be surpassed by GJO algorithm in the later period, when optimizing F21, it will be exceeded by ICOA, GJO algorithms in the later period, when optimizing F23 and F24, it will be exceeded by ICOA algorithms in the later period.

#### Analysis of box plot results

**Fig. 7:**
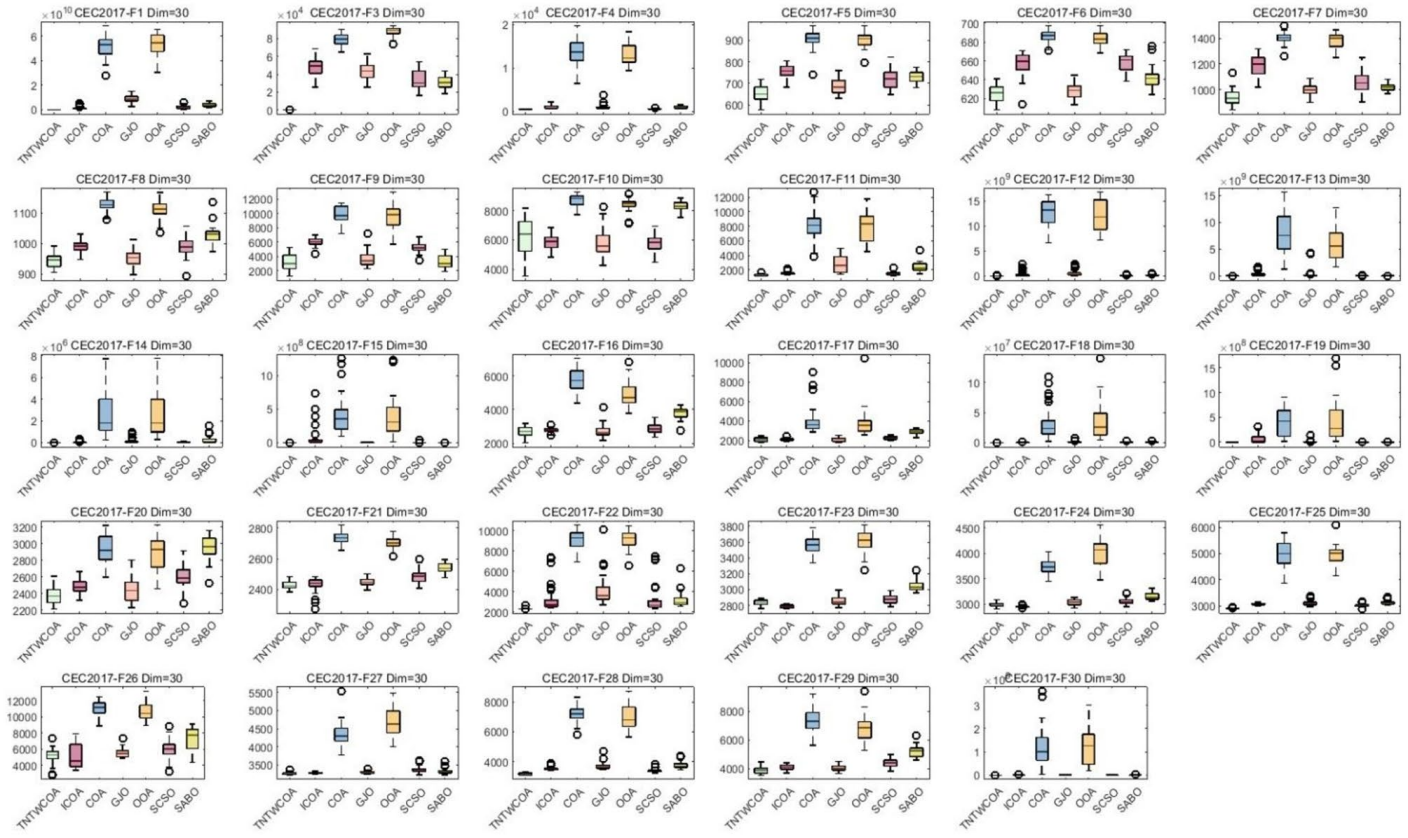
Boxplots for the proposed technique and other five algorithms for IEEE CEC2017 benchmark functions (Dim = 30).

A box chart is a statistical chart consisting of the smallest number (minimum value), the first quartile (25% locus value); The middle number (median value); The third quartile (75% locus value); The largest number (maximum value) constitutes. Figure 7 is the box graph obtained after 30 runs of the algorithm. The largest number (maximum value) and the smallest number (minimum value) in Fig. 5 constitute the variation range of the optimal values of 29 functions in CEC 2017 optimized by GJO, SCSO, OOA, SABO, COA, ICOA and TNTWCOA algorithms after running 30 times. That is, the narrower the box graph, the smaller the fluctuation range of the optimal value of the function running 30 times, and the more stable the optimization; The lower the position of the box diagram, the smaller the function optimization value and the closer it is to the theoretical value. The "o" in the diagram indicates the existence of singularity. As can be seen from Fig. 5, Except for the optimized the F10, F23, F24, F26, functions, all of the other has the lower the position of the box diagram, when optimized F6, F7, F9, F16, F17, F20, F27, the box diagram which is not the most narrower, when optimized F1, F2, F4, F11, F12, F13, F14, F15, F18, F19, F21, F22, F25, F28, F30, they have the smallest number (minimum value), the first quartile (25% point value) of the function box diagram; Middle digit (median value); The third quartile (75% locus value); The largest numbers (maximum) almost overlap.

#### Analysis of Wilcoxon rank sum test results

Wilcoxon rank sum test is a non-parametric statistical test. The difference between different algorithms is found by comparison. Table 3 shows the statistical results of TNTWCOA and five other algorithms over 30 runs. When the Wilcoxon comparison result is less than 0.05, it indicates that there is a significant deviation in the function optimization results between the two comparison algorithms; if the comparison result is greater than 0.05, it indicates that there is no significant deviation in the function optimization results between the two comparison algorithms. From the comparison results of TNTWCOA algorithm with ICOA, GJO, COA, SCSO, OOA and SABO algorithms, there is a significant deviation between TNTWCOA algorithm and most algorithms in function optimization results. However, when optimizing functions F6, F8, F9, F19, F16, F17, F23 and F26, the Wilcoxon rank sum test of TNTWCOA algorithm and GJO algorithm is greater than 0.05, indicating that there is no significant difference in the optimization values of the functions obtained. At the same time, when optimizing function F10, F16, F17, F21, F26 the Wilcoxon rank sum test of TNTWCOA algorithm and **ICOA** algorithm is greater than 0.05, indicating that there is no significant difference between the obtained function values, when optimizing function F10, F16, the Wilcoxon rank sum test of TNTWCOA algorithm and **SCSO** algorithm is greater than 0.05, indicating that there is no significant difference between the obtained function values, when optimizing function F9, the Wilcoxon rank sum test of TNTWCOA algorithm and SASBO algorithm is greater than 0.05, indicating that there is no significant difference between the obtained function values.

**Table 3:** Wilcoxon rank sum test results.

	ICOA	COA	GJO	OOA	SCSO	SABO
F1	3.01986E−11	3.01986E−11	3.01986E−11	3.01986E−11	3.01986E−11	3.01986E−11
F3	3.01986E−11	3.01986E−11	3.01986E−11	3.01986E−11	3.01986E−11	3.01986E−11
F4	3.01986E−11	3.01986E−11	3.01986E−11	3.01986E−11	1.46431E−10	3.01986E−11
F5	8.15274E−11	3.01986E−11	1.85748E−03	3.01986E−11	4.11271E−07	8.10136E−10
F6	8.89099E−10	3.01986E−11	**1.66866E−01**	3.01986E−11	5.49405E−11	8.35200E−08
F7	8.15274E−11	3.01986E−11	4.63897E−05	3.01986E−11	5.09117E−06	9.53321E−07
F8	4.57257E−09	3.01986E−11	**1.71450E−01**	3.01986E−11	2.37682E−07	6.06576E−11
F9	8.15274E−11	3.01986E−11	**8.23572E−02**	3.01986E−11	5.96731E−09	**6.41424E−01**
F10	**1.02326E−01**	8.99341E−11	**1.80900E−01**	5.07231E−10	**1.08690E−01**	3.47420E−10
F11	3.35195E−08	3.01986E−11	6.69552E−11	3.01986E−11	1.24932E−05	3.68973E−11
F12	1.32885E−10	3.01986E−11	4.97517E−11	3.01986E−11	3.82016E−10	1.61323E−10
F13	3.01986E−11	3.01986E−11	4.19968E−10	3.01986E−11	4.57257E−09	3.01986E−11
F14	9.46827E−03	3.01986E−11	1.28704E−09	3.01986E−11	1.99628E−05	6.69552E−11
F15	3.68973E−11	3.01986E−11	7.38908E−11	3.01986E−11	6.06576E−11	6.69552E−11
F16	**3.63222E−01**	3.01986E−11	**8.76635E−01**	3.01986E−11	**7.24456E−02**	1.09367E−10
F17	**8.76635E−01**	3.01986E−11	**5.99689E−01**	3.01986E−11	1.17107E−02	2.37147E−10
F18	3.64589E−08	3.01986E−11	9.26029E−09	3.01986E−11	1.72941E−07	9.26029E−09
F19	3.01986E−11	3.01986E−11	8.89099E−10	3.01986E−11	4.07716E−11	3.01986E−11
F20	2.43271E−05	3.33839E−11	4.20668E−02	6.06576E−11	1.87310E−07	3.33839E−11
F21	**1.95791E−01**	3.01986E−11	1.27321E−02	3.01986E−11	1.87310E−07	3.68973E−11
F22	1.20354E−10	3.01418E−11	3.01418E−11	3.01418E−11	1.46174E−10	4.96602E−11
F23	1.60621E−06	3.01986E−11	**1.41278E−01**	3.01986E−11	5.56994E−03	3.01986E−11
F24	1.42984E−05	3.01986E−11	2.62428E−03	3.01986E−11	2.27802E−05	1.77691E−10
F25	3.01986E−11	3.01986E−11	3.01986E−11	3.01986E−11	6.69552E−11	3.01986E−11
F26	**9.70516E−01**	3.01797E−11	**2.64320E−01**	3.01797E−11	3.67042E−03	6.52381E−07
F27	3.03174E−02	3.01986E−11	1.10577E−04	3.01986E−11	5.18568E−07	2.87897E−06
F28	3.01986E−11	3.01986E−11	3.01986E−11	3.01986E−11	9.91863E−11	3.01986E−11
F29	1.10577E−04	3.01986E−11	1.03147E−02	3.01986E−11	5.53286E−08	3.01986E−11
F30	3.68973E−11	3.01986E−11	3.01986E−11	3.01986E−11	3.33839E−11	3.01986E−11

Significant values are in bold.

### Experiments on CEC2017 benchmark functions (Dim = 500)

#### Statistics analysis

Table 4 shows the statistical results of IEEE CEC2017 benchmark functions in 50 dimensions optimized by TNTWCOA, ICOA, GJO, COA, SCSO, OOA and SABO algorithms. Also the Friedman values based on the average value of the IEEE CEC2017 benchmark functions optimized by the TNTWCOA, ICOA, GJO, COA, SCSO, OOA and SABO algorithms were statistically analyzed. As can be seen from Table 2. More or less evaluation indexes of TNTWCOA proposed in this paper is superior to that of the ICOA, GJO, COA, SCSO, OOA, and SABO algorithms; Among them, when F1, F4, F11, F12, F13, F14, F15, F18, F19, F20, F25, F27, F28 and F30 functions are optimized, all the evaluation indexes of TNTWCOA algorithm are optimal, showing excellent performance. When optimizing F3, F9 the performance of std value is inferior to that of ICOA algorithm, for F5, which is inferior to that of COA algorithm, for F8, F22, F26, which is inferior to that of OOA algorithm, When optimizing F10 and F27, only worse values performed worse than ICOA algorithm; When optimizing F6, std value performance is inferior to COA algorithm, avg, median, worse value performance is inferior to GJO algorithm, for F7 std value performance is inferior to OOA algorithm, worse value performance is inferior to GJO algorithm, for F16 std, median, worse value performance is inferior to GJO algorithm, for F17 std value performance is inferior to SCSO algorithm, avg value performance is inferior to GJO algorithm, for F21 std and worse value performance are inferior to ICOA algorithm, for F23 std value performance is inferior to GJO algorithm, avg, median, worse value performance are inferior to ICOA algorithm, for F24 std, avg, median, worse value performance are inferior to ICOA algorithm, For f29 std, avg, median, value performance are inferior to ICOA algorithm. In general, the improved algorithm shows stronger optimization ability in the case of 50 dimensions. Friedman's overall order is ICOA > SCSO > ICOA > GJO > SABO > COA > OOA. Therefore, from the statistical results of evaluation index, when Dim = 50, the algorithm proposed in this paper shows excellent performance compared with other six algorithms. Compared with the original COA algorithm and ICOA, the statistical results of evaluation index of the improved algorithm have been significantly improved.

**Table 4:** The statistical Results of benchmark functions using the proposed technique and other five algorithms (Dim = 50).

Function	Item	TNTWCOA	ICOA	COA	GJO	OOA	SCSO	SABO
F1	Min	**1.03078E+02**	3.14174E+09	9.18904E+10	2.10152E+10	9.38699E+10	2.17836E+09	7.68053E+09
	Std	**7.18120E+03**	3.82030E+09	8.08270E+09	6.49093E+09	7.47457E+09	6.38028E+09	3.66232E+09
	Avg	**7.97902E+03**	8.40231E+09	1.11832E+11	3.39585E+10	1.13983E+11	1.41959E+10	1.58685E+10
	Median	**9.92422E+03**	7.23931E+09	1.13298E+11	3.40948E+10	1.13875E+11	1.33278E+10	1.50015E+10
	Worse	**3.18377E+04**	1.72854E+10	1.23045E+11	4.47081E+10	1.25197E+11	2.83196E+10	2.30080E+10
F3	Min	**2.65194E+04**	9.07989E+04	1.59642E+05	7.68222E+04	1.72961E+05	5.46018E+04	1.15053E+05
	Std	1.90880E+04	**1.47103E+04**	1.67609E+04	1.79644E+04	1.79884E+04	1.70070E+04	2.37406E+04
	Avg	**5.63077E+04**	1.16422E+05	1.86950E+05	1.10508E+05	2.14285E+05	8.73036E+04	1.54657E+05
	Median	**5.72644E+04**	1.14042E+05	1.89201E+05	1.11413E+05	2.15445E+05	8.97551E+04	1.55651E+05
	Worse	**1.06743E+05**	1.62012E+05	2.18215E+05	1.44440E+05	2.48025E+05	1.22488E+05	2.07019E+05
F4	Min	**4.18323E+02**	8.17833E+02	2.92938E+04	2.81567E+03	2.77103E+04	1.04624E+03	1.56433E+03
	Std	**4.94839E+01**	5.67939E+02	5.73968E+03	1.28632E+03	5.65435E+03	8.78584E+02	1.46263E+03
	Avg	**4.84009E+02**	1.58805E+03	3.90597E+04	4.97129E+03	3.83638E+04	2.05739E+03	3.27983E+03
	Median	**4.84474E+02**	1.64194E+03	3.94128E+04	4.86786E+03	3.77968E+04	1.76131E+03	2.87987E+03
	Worse	**5.57652E+02**	3.68898E+03	4.75176E+04	7.74612E+03	4.89001E+04	4.37215E+03	8.04042E+03
F5	Min	**7.19982E+02**	9.23786E+02	1.10106E+03	7.90575E+02	1.08975E+03	8.19109E+02	9.02816E+02
	Std	4.14917E+01	3.42492E+01	**3.31881E+01**	4.64338E+01	3.67168E+01	3.80548E+01	5.73979E+01
	Avg	**8.42174E+02**	9.77129E+02	1.18913E+03	8.78452E+02	1.19325E+03	9.02193E+02	1.02089E+03
	Median	**8.50223E+02**	9.75969E+02	1.18856E+03	8.72370E+02	1.19505E+03	8.92673E+02	1.02522E+03
	Worse	**9.18874E+02**	1.03933E+03	1.23592E+03	9.85147E+02	1.25762E+03	9.67986E+02	1.14495E+03
F6	Min	**6.29641E+02**	6.63773E+02	6.90786E+02	6.36020E+02	6.88593E+02	6.59556E+02	6.56299E+02
	Std	9.73512E+00	4.88066E+00	**3.97052E+00**	4.86639E+00	4.65900E+00	5.86331E+00	1.11980E+01
	Avg	6.50034E+02	6.75897E+02	7.00846E+02	**6.48271E+02**	7.01559E+02	6.71799E+02	6.74928E+02
	Median	6.53414E+02	6.75513E+02	7.01305E+02	**6.47894E+02**	7.02308E+02	6.72647E+02	6.72294E+02
	Worse	6.64464E+02	6.86199E+02	7.06577E+02	**6.58311E+02**	7.07532E+02	6.81744E+02	7.01315E+02
F7	Min	**1.08352E+03**	1.60761E+03	1.91303E+03	1.19226E+03	1.94049E+03	1.21083E+03	1.38389E+03
	Std	1.42872E+02	1.04460E+02	6.11165E+01	7.94572E+01	**5.93591E+01**	1.33791E+02	8.94709E+01
	Avg	**1.29881E+03**	1.91409E+03	2.05851E+03	1.40948E+03	2.05126E+03	1.60476E+03	1.55735E+03
	Median	**1.28188E+03**	1.93909E+03	2.08163E+03	1.40638E+03	2.06622E+03	1.64020E+03	1.55799E+03
	Worse	1.60358E+03	2.05889E+03	2.14301E+03	**1.52145E+03**	2.14916E+03	1.83114E+03	1.74258E+03
F8	Min	**1.01590E+03**	1.19339E+03	1.40890E+03	1.12221E+03	1.42593E+03	1.12607E+03	1.25430E+03
	Std	4.77444E+01	3.76316E+01	3.53863E+01	6.64804E+01	**2.97032E+01**	4.20961E+01	4.19404E+01
	Avg	**1.11976E+03**	1.25973E+03	1.50287E+03	1.21514E+03	1.49872E+03	1.21639E+03	1.34213E+03
	Median	**1.13032E+03**	1.25931E+03	1.50863E+03	1.20592E+03	1.49514E+03	1.22117E+03	1.34187E+03
	Worse	**1.21390E+03**	1.35773E+03	1.56640E+03	1.36754E+03	1.55660E+03	1.29559E+03	1.43606E+03
F9	Min	**3.34108E+03**	1.85660E+04	2.84748E+04	9.30371E+03	3.00113E+04	1.29721E+04	1.34388E+04
	Std	2.95853E+03	**1.53260E+03**	3.25707E+03	6.28109E+03	3.11020E+03	2.86511E+03	5.40160E+03
	Avg	**8.49914E+03**	2.11830E+04	3.57122E+04	1.81564E+04	3.53018E+04	1.69420E+04	2.11783E+04
	Median	**7.94172E+03**	2.11572E+04	3.58860E+04	1.69802E+04	3.46387E+04	1.63008E+04	2.02404E+04
	Worse	**1.66216E+04**	2.59237E+04	4.15408E+04	2.99127E+04	4.06095E+04	2.51770E+04	3.26187E+04
F10	Min	**5.23396E+03**	7.99449E+03	1.40149E+04	7.43882E+03	1.35370E+04	7.38015E+03	1.26463E+04
	Std	**1.80796E+03**	4.77805E+02	5.05073E+02	1.75246E+03	6.44440E+02	9.91042E+02	5.70754E+02
	Avg	**7.73718E+03**	9.01232E+03	1.54288E+04	9.63559E+03	1.48631E+04	9.08172E+03	1.45761E+04
	Median	**7.35713E+03**	8.96092E+03	1.55271E+04	9.23739E+03	1.48263E+04	8.98988E+03	1.47243E+04
	Worse	1.33135E+04	**9.92810E+03**	1.63786E+04	1.46629E+04	1.62380E+04	1.10229E+04	1.54082E+04
F11	Min	**1.33987E+03**	3.32252E+03	2.30322E+04	3.06823E+03	1.44354E+04	1.49254E+03	2.03677E+03
	Std	**8.12215E+01**	1.54965E+03	1.90145E+03	2.66629E+03	3.71710E+03	2.18620E+03	2.01476E+03
	Avg	**1.48728E+03**	6.85094E+03	2.63193E+04	8.47119E+03	2.67447E+04	4.57246E+03	4.57914E+03
	Median	**1.49206E+03**	6.68497E+03	2.66916E+04	8.57826E+03	2.78683E+04	4.14673E+03	4.46764E+03
	Worse	**1.63813E+03**	9.55716E+03	3.02844E+04	1.23641E+04	3.16310E+04	1.00537E+04	1.01616E+04
F12	Min	**9.17010E+05**	4.13204E+08	5.16629E+10	2.17968E+09	5.60990E+10	3.84838E+07	7.88907E+08
	Std	**2.42670E+07**	2.57574E+09	1.91418E+10	4.20722E+09	1.51524E+10	2.25866E+09	2.83365E+09
	Avg	**2.19845E+07**	2.78370E+09	8.89335E+10	7.78423E+09	8.39365E+10	1.78642E+09	4.52417E+09
	Median	**1.20553E+07**	1.77241E+09	9.21145E+10	6.73266E+09	8.35753E+10	1.17218E+09	3.97129E+09
	Worse	**8.71600E+07**	9.56952E+09	1.23273E+11	2.16494E+10	1.20610E+11	1.17088E+10	9.49459E+09
F13	Min	**4.59699E+03**	2.28390E+08	1.96614E+10	2.23634E+08	2.58041E+10	1.74813E+05	1.00870E+08
	Std	**1.42842E+04**	2.68931E+09	1.27545E+10	2.41076E+09	1.52918E+10	1.55286E+08	8.31861E+08
	Avg	**2.92092E+04**	2.91832E+09	4.94992E+10	2.12441E+09	5.04556E+10	1.36596E+08	6.36896E+08
	Median	**3.16954E+04**	2.23650E+09	4.98536E+10	1.26885E+09	4.80272E+10	5.15481E+07	3.24770E+08
	Worse	**5.18332E+04**	1.18639E+10	6.97531E+10	9.24152E+09	8.53274E+10	6.95366E+08	3.88126E+09
F14	Min	**9.45251E+03**	7.92096E+04	1.24270E+07	5.60153E+04	1.05955E+07	2.47503E+04	2.70620E+05
	Std	**7.34462E+04**	2.38342E+06	8.65615E+07	3.04350E+06	1.10597E+08	1.13621E+06	2.20742E+06
	Avg	**9.96998E+04**	1.47016E+06	1.11255E+08	2.00812E+06	1.58931E+08	9.65869E+05	3.19450E+06
	Median	**1.01889E+05**	6.69283E+05	9.46313E+07	1.00484E+06	1.19284E+08	5.96728E+05	3.03930E+06
	Worse	**2.86653E+05**	1.01383E+07	3.34507E+08	1.47373E+07	3.52115E+08	4.73791E+06	7.71051E+06
F15	Min	**2.65317E+03**	5.96916E+07	1.50619E+09	2.60203E+04	2.02565E+09	3.25833E+04	8.61450E+05
	Std	**7.01066E+03**	2.21984E+09	3.79334E+09	3.64484E+08	3.67368E+09	1.12484E+08	1.79362E+08
	Avg	**1.57439E+04**	2.10852E+09	1.02141E+10	2.58192E+08	9.28890E+09	2.94620E+07	5.80016E+07
	Median	**2.07985E+04**	1.20066E+09	1.00803E+10	7.12990E+07	8.39049E+09	6.33283E+04	9.76328E+06
	Worse	**2.27995E+04**	7.97063E+09	1.80434E+10	1.04491E+09	1.77204E+10	6.14525E+08	9.76078E+08
F16	Min	**3.05813E+03**	3.50819E+03	7.62708E+03	2.99640E+03	6.80627E+03	3.23894E+03	4.00633E+03
	Std	4.96238E+02	5.13843E+02	1.48222E+03	**3.97631E+02**	2.14090E+03	5.78487E+02	6.34067E+02
	Avg	**3.77877E+03**	4.36937E+03	1.04250E+04	3.80880E+03	9.92593E+03	4.22684E+03	5.18922E+03
	Median	3.84530E+03	4.28808E+03	1.03359E+04	**3.77728E+03**	9.36249E+03	4.23728E+03	5.34092E+03
	Worse	4.79665E+03	5.90139E+03	1.45447E+04	**4.73748E+03**	1.53977E+04	5.48742E+03	6.27629E+03
F17	Min	**2.50960E+03**	3.41229E+03	4.66562E+03	2.70337E+03	4.81980E+03	2.93035E+03	3.24250E+03
	Std	3.79451E+02	5.67566E+02	1.35939E+04	4.80681E+02	1.02713E+04	**3.42101E+02**	4.35897E+02
	Avg	3.27755E+03	3.92491E+03	1.40951E+04	**3.41694E+03**	1.32346E+04	3.71736E+03	4.39127E+03
	Median	**3.18997E+03**	3.76749E+03	8.63494E+03	3.36018E+03	8.98660E+03	3.69312E+03	4.43076E+03
	Worse	**3.97346E+03**	5.95867E+03	5.40202E+04	4.96586E+03	4.93026E+04	4.49485E+03	5.29406E+03
F18	Min	**8.46577E+04**	1.73557E+06	5.65729E+07	2.09898E+05	3.66241E+07	1.45364E+05	1.41686E+06
	Std	**2.66617E+05**	8.80330E+06	1.26555E+08	2.11290E+07	1.27065E+08	1.04463E+07	1.11128E+07
	Avg	**2.83633E+05**	1.07193E+07	2.01664E+08	1.22793E+07	2.38402E+08	5.49538E+06	9.52607E+06
	Median	**1.99813E+05**	7.22919E+06	1.66132E+08	3.72833E+06	2.13372E+08	1.92625E+06	4.54980E+06
	Worse	**1.26662E+06**	2.92400E+07	6.23305E+08	1.00810E+08	5.78432E+08	5.47831E+07	4.99252E+07
F19	Min	**7.28433E+03**	6.38275E+06	1.25804E+09	9.01916E+04	5.00087E+08	4.76153E+04	6.74577E+05
	Std	**1.26916E+04**	4.81276E+08	1.71529E+09	2.10465E+08	1.76662E+09	8.46140E+06	9.99686E+06
	Avg	**2.65520E+04**	5.28233E+08	4.06259E+09	1.34809E+08	3.96595E+09	3.47382E+06	8.46779E+06
	Median	**2.26731E+04**	3.91428E+08	4.07151E+09	6.52689E+07	3.42602E+09	1.16476E+06	4.89243E+06
	Worse	**4.73551E+04**	1.68359E+09	7.17954E+09	1.04077E+09	7.66906E+09	3.49019E+07	4.04726E+07
F20	Min	**2.76637E+03**	3.02706E+03	3.57553E+03	2.66786E+03	3.58457E+03	2.82551E+03	3.27147E+03
	Std	**1.85323E+02**	2.87427E+02	2.55503E+02	3.88834E+02	3.00177E+02	2.73340E+02	2.57695E+02
	Avg	**3.14008E+03**	3.53983E+03	4.15576E+03	3.26889E+03	4.17085E+03	3.44451E+03	3.94471E+03
	Median	**3.15891E+03**	3.55061E+03	4.16459E+03	3.22813E+03	4.18004E+03	3.44842E+03	3.99790E+03
	Worse	**3.57490E+03**	4.06720E+03	4.52635E+03	4.09112E+03	4.58725E+03	3.84354E+03	4.32866E+03
F21	Min	**2.48130E+03**	2.57291E+03	3.04251E+03	2.59556E+03	3.05098E+03	2.66273E+03	2.75769E+03
	Std	6.60259E+01	**3.14731E+01**	9.48901E+01	6.03134E+01	9.51919E+01	5.89877E+01	6.28697E+01
	Avg	**2.59130E+03**	2.62927E+03	3.27808E+03	2.68713E+03	3.21081E+03	2.77172E+03	2.87982E+03
	Median	**2.57956E+03**	2.63196E+03	3.27318E+03	2.67304E+03	3.22391E+03	2.77651E+03	2.87406E+03
	Worse	2.76219E+03	**2.68424E+03**	3.45342E+03	2.87144E+03	3.38372E+03	2.90487E+03	3.00472E+03
F22	Min	**2.30000E+03**	9.75080E+03	1.60149E+04	8.59188E+03	1.60242E+04	6.94851E+03	7.63287E+03
	Std	3.82012E+03	6.61560E+02	4.59425E+02	2.10566E+03	**4.58405E+02**	1.34311E+03	2.29588E+03
	Avg	**5.73184E+03**	1.12135E+04	1.70473E+04	1.21329E+04	1.68378E+04	1.13434E+04	1.54351E+04
	Median	**2.32729E+03**	1.12629E+04	1.69915E+04	1.17512E+04	1.68240E+04	1.15405E+04	1.61683E+04
	Worse	**1.11467E+04**	1.24181E+04	1.81776E+04	1.65582E+04	1.79020E+04	1.33947E+04	1.70473E+04
F23	Min	**2.96397E+03**	3.00042E+03	4.02894E+03	3.05339E+03	4.22403E+03	3.09090E+03	3.43784E+03
	Std	9.74805E+01	4.44858E+01	2.30169E+02	**7.60341E+01**	1.45640E+02	1.00586E+02	1.41774E+02
	Avg	3.17966E+03	**3.12884E+03**	4.57099E+03	3.25458E+03	4.58122E+03	3.30903E+03	3.74409E+03
	Median	3.17832E+03	**3.12477E+03**	4.58512E+03	3.24784E+03	4.59830E+03	3.30679E+03	3.73981E+03
	Worse	3.42406E+03	**3.19794E+03**	4.91497E+03	3.37812E+03	4.86465E+03	3.50418E+03	4.10030E+03
F24	Min	**3.11911E+03**	3.16023E+03	4.50909E+03	3.27349E+03	5.01908E+03	3.27937E+03	3.57062E+03
	Std	8.98337E+01	**4.81669E+01**	3.23377E+02	1.02717E+02	3.75675E+02	1.13490E+02	1.65928E+02
	Avg	3.30959E+03	**3.25541E+03**	4.97469E+03	3.41111E+03	5.69474E+03	3.45717E+03	3.87407E+03
	Median	3.30462E+03	**3.25301E+03**	4.86019E+03	3.38993E+03	5.67623E+03	3.43464E+03	3.85495E+03
	Worse	3.49001E+03	**3.35248E+03**	6.06730E+03	3.65645E+03	6.29589E+03	3.76378E+03	4.33316E+03
F25	Min	**2.97051E+03**	3.56298E+03	1.19760E+04	4.63503E+03	1.25996E+04	3.45829E+03	3.87019E+03
	Std	**4.45822E+01**	3.40076E+02	1.30738E+03	6.81716E+02	1.50031E+03	5.14074E+02	7.67228E+02
	Avg	**3.04906E+03**	4.11784E+03	1.58249E+04	5.55678E+03	1.61275E+04	4.12944E+03	4.97509E+03
	Median	**3.06211E+03**	4.02744E+03	1.58314E+04	5.39576E+03	1.63742E+04	4.02735E+03	4.95022E+03
	Worse	**3.14170E+03**	5.08199E+03	1.78416E+04	7.29241E+03	1.86071E+04	5.23277E+03	6.37878E+03
F26	Min	**2.90000E+03**	5.54125E+03	1.49759E+04	7.94029E+03	1.51706E+04	7.07205E+03	7.97888E+03
	Std	3.14014E+03	1.83156E+03	9.11992E+02	9.09928E+02	**8.69461E+02**	1.84060E+03	1.55069E+03
	Avg	**4.66026E+03**	1.13268E+04	1.73815E+04	9.55374E+03	1.74354E+04	1.09870E+04	1.26090E+04
	Median	**2.90000E+03**	1.18992E+04	1.74105E+04	9.62440E+03	1.75589E+04	1.18264E+04	1.31576E+04
	Worse	1.15389E+04	1.37569E+04	1.88173E+04	**1.13849E+04**	1.88137E+04	1.33554E+04	1.46637E+04
F27	Min	**3.42601E+03**	3.59820E+03	5.27659E+03	3.67298E+03	6.18651E+03	3.63348E+03	3.84979E+03
	Std	**1.74556E+02**	9.09040E+01	7.80947E+02	1.68440E+02	6.51714E+02	2.44033E+02	2.61758E+02
	Avg	**3.68017E+03**	3.75484E+03	7.11308E+03	3.98044E+03	7.67549E+03	4.11461E+03	4.37230E+03
	Median	**3.66403E+03**	3.74701E+03	7.02782E+03	3.95328E+03	7.68578E+03	4.11518E+03	4.38025E+03
	Worse	4.20894E+03	**3.99494E+03**	8.41461E+03	4.35034E+03	8.94494E+03	4.72746E+03	4.84284E+03
F28	Min	**3.25810E+03**	3.92545E+03	9.88068E+03	4.75202E+03	1.14464E+04	3.91960E+03	4.52830E+03
	Std	**3.27757E+01**	3.11825E+02	1.40980E+03	6.11193E+02	1.49666E+03	4.55094E+02	6.43432E+02
	Avg	**3.30456E+03**	4.38339E+03	1.35798E+04	5.95332E+03	1.41904E+04	4.71098E+03	5.90777E+03
	Median	**3.30269E+03**	4.34063E+03	1.33559E+04	5.83664E+03	1.41286E+04	4.58138E+03	6.02567E+03
	Worse	**3.41163E+03**	5.17803E+03	1.60434E+04	7.19020E+03	1.70619E+04	5.66278E+03	6.90582E+03
F29	Min	**3.97729E+03**	4.88579E+03	1.44639E+04	4.70750E+03	2.04784E+04	5.53239E+03	5.70092E+03
	Std	4.58167E+02	**3.28396E+02**	1.02387E+05	6.10081E+02	3.17131E+05	5.20345E+02	2.42283E+03
	Avg	4.88462E+03	**5.41244E+03**	9.28786E+04	5.65474E+03	1.82497E+05	6.45067E+03	9.22342E+03
	Median	4.88097E+03	**5.31311E+03**	6.78781E+04	5.49729E+03	6.49525E+04	6.41979E+03	8.91032E+03
	Worse	**5.80506E+03**	6.17725E+03	5.55088E+05	7.35799E+03	1.41926E+06	7.66830E+03	1.88425E+04
F30	Min	**1.36232E+06**	1.79320E+07	3.21236E+09	1.06840E+08	1.33951E+09	4.37517E+07	1.00998E+08
	Std	**2.83402E+06**	4.47310E+08	3.46178E+09	5.48798E+08	2.57567E+09	5.44831E+07	1.20576E+08
	Avg	**4.94706E+06**	5.38679E+08	8.55793E+09	3.90432E+08	7.58475E+09	1.15410E+08	2.89244E+08
	Median	**4.05712E+06**	5.20179E+08	8.43092E+09	2.36187E+08	7.66057E+09	1.09887E+08	2.61210E+08
	Worse	**1.11712E+07**	1.75038E+09	1.66654E+10	3.02166E+09	1.24080E+10	2.93465E+08	6.41403E+08
Friedman	Value	1.10345E+00	3.34483E+00	6.44828E+00	3.41379E+00	6.55172E+00	2.86207E+00	4.27586E+00
	Rank	1	3	6	4	7	2	5

Significant values are in bold.

#### Convergence analysis

**Fig. 8:**
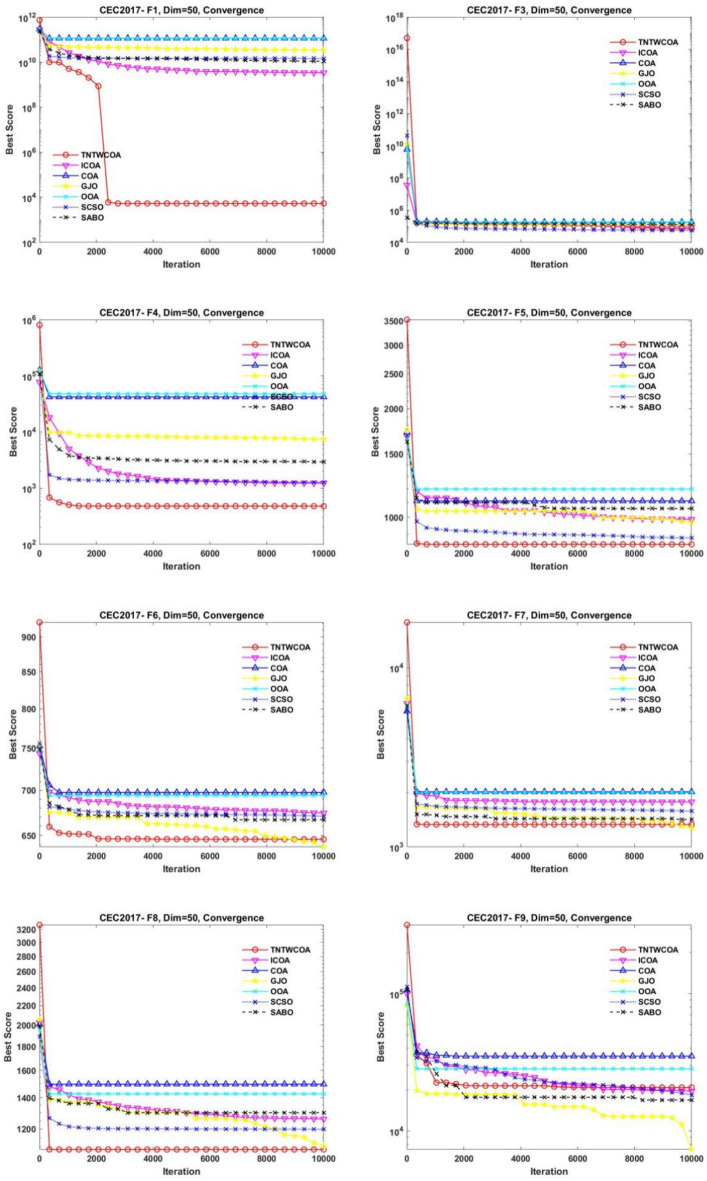

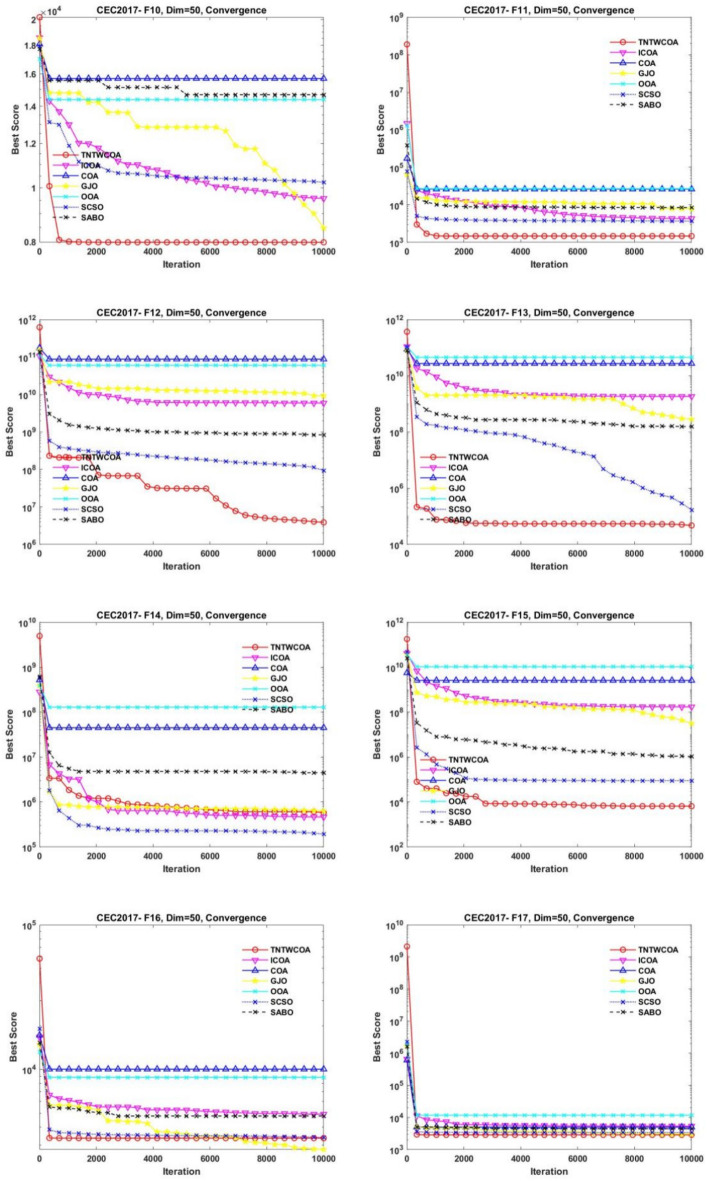

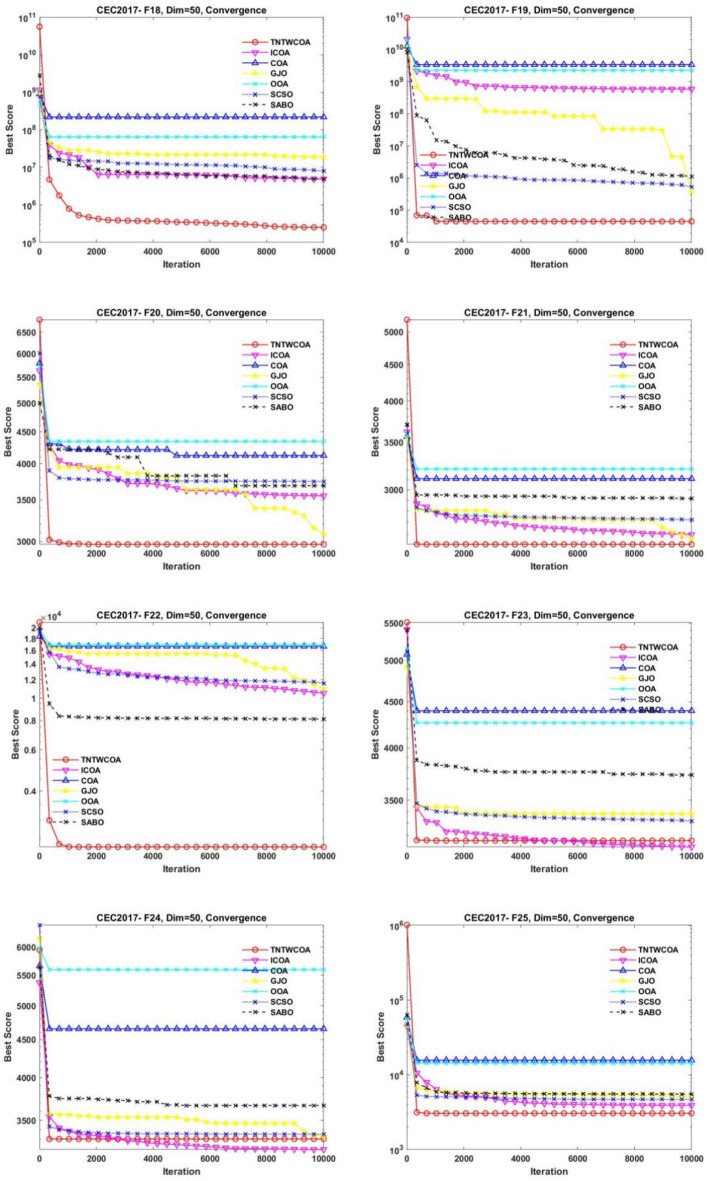

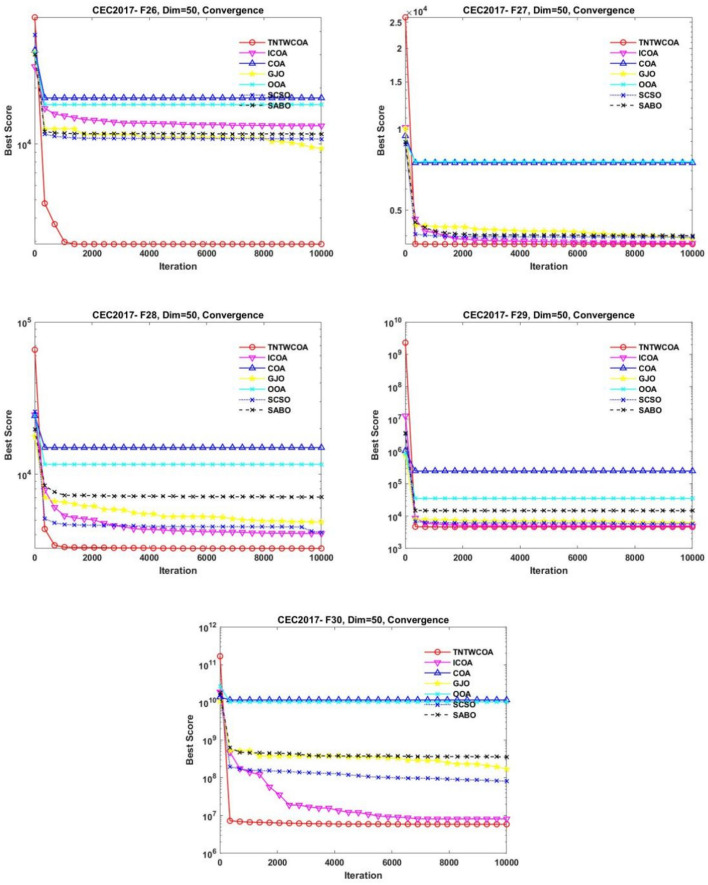
The convergence curves of the proposed technique and other five algorithms for IEEE CEC2017 benchmark functions (Dim = 50).

Figure 8 illustrates the convergence curves of GJO, SCSO, OOA, SABO, original COA, and ICOA on 29 benchmark functions of the IEEE CEC2017 throughout the iterations with 10000times.

As can be seen from Fig. 8, the convergence speed of the improved TNTWCOA algorithm is optimal; Among them, when the optimization functionF1, F3, F4, F8, F10, F11, F13, F15, F18, F19, F20, F21, F22, F25, FF26, F27, F28, F29, F30, it can quickly converge to the best value and remain stable. When the optimization function F12, it can quickly converge to the best value and will continue to optimize to make the best better. When optimizing functions F6, F7, F16, F23, F24, it can quickly converge to the best value and remain stable, but the best value will be surpassed by other algorithms in the later period, such as optimizing F6, F7, F16, it will be surpassed by GJO algorithm in the later period, and for F23, F24 it will be surpassed by ICOA algorithm in the later period. When optimizing functions F3, F9, F14, it cannot converge to the best value quickly, but it will gradually converge to the best and better and surpass other algorithms.

#### Analysis of box plot results

**Fig. 9:**
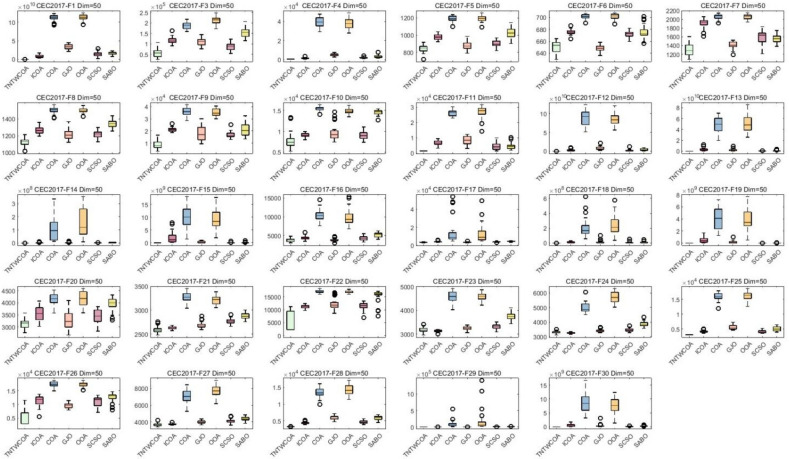
Boxplots for the proposed technique and other five algorithms for IEEE CEC2017 benchmark functions (Dim = 50).

The largest number (maximum value) and the smallest number (minimum value) in Fig. 7 constitute the variation range of the optimal values of 29 functions in CEC 2017 optimized by GJO, SCSO, OOA, SABO, COA, and ICOA algorithms after running 50 times. That is, the narrower the box graph, the smaller the fluctuation range of the optimal value of the function running 30 times, and the more stable the optimization; The lower the position of the box diagram, the smaller the function optimization value and the closer it is to the theoretical value. The "o" in the diagram indicates the existence of singularity. As can be seen from Fig. 9, all of them have the lower the position of the box diagram, when optimized F22, F27, the narrow of the box diagram is worst, when optimized F1, F4, F11, F12, F13, F14, F15, F17, F18, F19, F20, F25, F28, F29, F30, they have the smallest number (minimum value), the first quartile (25% point value) of the function box diagram; Middle digit (median value); The third quartile (75% locus value); The largest numbers (maximum) almost overlap.

#### Analysis of Wilcoxon rank sum test results

Table 5 shows the Wilcoxon rank sum test results for ICOA and 5 different algorithms over 50 runs. From the comparison of TNTWCOA algorithm with ICOA, GJO, COA, SCSO, OOA, SABO algorithm, there is a significant deviation between TNTWCOA algorithm and most algorithms. However, when optimizing functions F7, F16, F17, F20 and F23, the Wilcoxon rank sum test of TNTWCOA algorithm and COA algorithm is greater than 0.05, indicating that there is no significant difference between the obtained function values.

**Table 5:** Wilcoxon rank sum test results.

	TNTWCOA	ICOA	COA	GJO	OOA	SCSO
F1	3.01986E−11	3.01986E−11	3.01986E−11	3.01986E−11	3.01986E−11	3.01986E−11
F3	6.06576E−11	3.01986E−11	1.95678E−10	3.01986E−11	2.19589E−07	3.01986E−11
F4	3.01986E−11	3.01986E−11	3.01986E−11	3.01986E−11	3.01986E−11	3.01986E−11
F5	3.01986E−11	3.01986E−11	5.82817E−03	3.01986E−11	1.02773E−06	3.68973E−11
F6	3.33839E−11	3.01986E−11	**2.17017E−01**	3.01986E−11	8.99341E−11	3.47420E−10
F7	3.01986E−11	3.01986E−11	1.17376E−03	3.01986E−11	5.46175E−09	1.42942E−08
F8	6.69552E−11	3.01986E−11	1.15665E−07	3.01986E−11	5.46175E−09	3.01986E−11
F9	3.01986E−11	3.01986E−11	3.49711E−09	3.01986E−11	1.77691E−10	6.06576E−11
F10	4.11776E−06	3.01986E−11	2.67842E−06	3.01986E−11	1.52917E−05	3.68973E−11
F11	3.01986E−11	3.01986E−11	3.01986E−11	3.01986E−11	1.32885E−10	3.01986E−11
F12	3.01986E−11	3.01986E−11	3.01986E−11	3.01986E−11	8.15274E−11	3.01986E−11
F13	3.01986E−11	3.01986E−11	3.01986E−11	3.01986E−11	3.01986E−11	3.01986E−11
F14	5.53286E−08	3.01986E−11	4.61591E−10	3.01986E−11	8.35200E−08	3.33839E−11
F15	3.01986E−11	3.01986E−11	3.01986E−11	3.01986E−11	3.01986E−11	3.01986E−11
F16	6.76501E−05	3.01986E−11	**8.64994E−01**	3.01986E−11	4.63712E−03	9.75550E−10
F17	3.83494E−06	3.01986E−11	**3.47828E−01**	3.01986E−11	7.65879E−05	2.87158E−10
F18	3.01986E−11	3.01986E−11	1.46431E−10	3.01986E−11	5.46175E−09	3.01986E−11
F19	3.01986E−11	3.01986E−11	3.01986E−11	3.01986E−11	3.01986E−11	3.01986E−11
F20	8.19750E−07	3.01986E−11	**3.18304E−01**	3.01986E−11	1.52917E−05	7.38908E−11
F21	1.51780E−03	3.01986E−11	8.19750E−07	3.01986E−11	3.82016E−10	3.33839E−11
F22	1.31110E−08	3.01986E−11	2.92155E−09	3.01986E−11	1.69795E−08	4.19968E−10
F23	6.37725E−03	3.01986E−11	9.52074E−04	3.01986E−11	1.24932E−05	3.01986E−11
F24	7.61706E−03	3.01986E−11	3.00589E−04	3.01986E−11	3.09389E−06	3.01986E−11
F25	3.01986E−11	3.01986E−11	3.01986E−11	3.01986E−11	3.01986E−11	3.01986E−11
F26	5.96731E−09	3.01986E−11	3.83494E−06	3.01986E−11	7.77255E−09	2.37147E−10
F27	9.46827E−03	3.01986E−11	1.35943E−07	3.01986E−11	1.01045E−08	1.20567E−10
F28	3.01986E−11	3.01986E−11	3.01986E−11	3.01986E−11	3.01986E−11	3.01986E−11
F29	1.42984E−05	3.01986E−11	1.60621E−06	3.01986E−11	4.50432E−11	3.33839E−11
F30	3.01986E−11	3.01986E−11	3.01986E−11	3.01986E−11	3.01986E−11	3.01986E−11

Significant values are in bold.

### Experiments on IEEE CEC2017 (Dim = 100)

#### Statistics analysis

Table 6 shows the statistical results of IEEE CEC2017 benchmark functions in 100 dimensions optimized by TNTWCOA, ICOA, GJO, COA, SCSO, OOA and SABO algorithms. Also the Friedman values based on the average value of the IEEE CEC2017 benchmark functions optimized by the TNTWCOA, ICOA, GJO, COA, SCSO, OOA and SABO algorithms were statistically analyzed. As can be seen from Table 2. More or less evaluation indexes of TNTWCOA proposed in this paper is superior to that of the ICOA, GJO, COA, SCSO, OOA, and SABO algorithms; Among them, when F1, F4, F11, F12, F13, F14, F15, F16, F18, F19, F25, F28, F29 and F30 functions are optimized, all the evaluation indexes of TNTWCOA algorithm are optimal, showing excellent performance; When F3, F7, F8, F27 are optimized, std value only is inferior to ICOA algorithm,F5 are optimized, min and std value only is inferior to ICOA algorithm, and for F21,std and worse value only is inferior to ICOA algorithm; for 24, avg, median, worse value only is inferior to ICOA algorithm, F6, F22 are optimized, std value only is inferior to COA algorithm, min value only is inferior to GJO algorithm and std, worse value only is inferior to ICOA algorithm for F20, std value only is inferior to COA algorithm and worse value only is inferior to SCSO algorithm for F9, std value only is inferior to COA algorithm and worse value only is inferior to ICOA algorithm for F10, F26 MIN ICOA STD GJO, min value only is inferior to ICOA algorithm and std value only is inferior to GJO algorithm for F26. In general, the improved algorithm shows stronger optimization ability in the case of 50 dimensions.

**Table 6:** The statistical results of benchmark functions using the proposed technique and other five algorithms (Dim = 100).

Function	Item	TNTWCOA	ICOA	COA	GJO	OOA	SCSO	SABO
F1	Min	**2.05150E+02**	1.05680E+10	2.58244E+11	9.95712E+10	2.49541E+11	4.23964E+10	4.98358E+10
	Std	**5.89723E+07**	5.52733E+09	7.83387E+09	1.44989E+10	9.77254E+09	1.18765E+10	1.28540E+10
	Avg	**3.74083E+07**	2.22863E+10	2.73958E+11	1.29746E+11	2.70425E+11	6.18885E+10	7.77567E+10
	Median	**2.29591E+04**	2.17916E+10	2.74128E+11	1.30040E+11	2.70634E+11	6.28063E+10	7.77768E+10
	Worse	**1.84148E+08**	3.37478E+10	2.92416E+11	1.57094E+11	2.87141E+11	8.99748E+10	1.11549E+11
F3	Min	**1.99064E+05**	2.90787E+05	3.19343E+05	2.27351E+05	3.53109E+05	2.11375E+05	2.60337E+05
	Std	2.39746E+04	**1.17723E+04**	1.30916E+04	2.26259E+04	8.69949E+04	2.31731E+04	1.43433E+04
	Avg	**2.50904E+05**	3.12490E+05	3.43851E+05	2.67030E+05	4.37638E+05	2.51878E+05	2.87804E+05
	Median	**2.53869E+05**	3.12568E+05	3.46404E+05	2.68431E+05	4.09294E+05	2.54285E+05	2.88357E+05
	Worse	**2.92045E+05**	3.36277E+05	3.66282E+05	3.27713E+05	6.45460E+05	2.89379E+05	3.18618E+05
F4	Min	**5.73928E+02**	2.15737E+03	9.16254E+04	1.22475E+04	8.90961E+04	3.28104E+03	8.57233E+03
	Std	**4.15161E+01**	7.34284E+02	1.18586E+04	5.54715E+03	1.42660E+04	2.49995E+03	3.90309E+03
	Avg	**6.56881E+02**	3.16918E+03	1.16747E+05	1.90271E+04	1.12459E+05	7.14016E+03	1.42700E+04
	Median	**6.50713E+02**	3.12242E+03	1.19468E+05	1.83647E+04	1.12379E+05	6.52579E+03	1.29764E+04
	Worse	**7.60158E+02**	5.44125E+03	1.36466E+05	3.74381E+04	1.36938E+05	1.31848E+04	2.40801E+04
F5	Min	1.19547E+03	**1.59950E+03**	2.06286E+03	1.36742E+03	2.01208E+03	1.33642E+03	1.67806E+03
	Std	5.29697E+01	**4.10898E+01**	3.71057E+01	1.17853E+02	4.56658E+01	6.48618E+01	7.97271E+01
	Avg	**1.33625E+03**	1.69597E+03	2.13454E+03	1.54984E+03	2.13075E+03	1.49451E+03	1.80266E+03
	Median	**1.33774E+03**	1.70525E+03	2.13638E+03	1.54535E+03	2.14344E+03	1.50618E+03	1.78707E+03
	Worse	**1.43127E+03**	1.75681E+03	2.19778E+03	1.85384E+03	2.19884E+03	1.63004E+03	1.97337E+03
F6	Min	**6.46166E+02**	6.82517E+02	7.05847E+02	6.61226E+02	6.99532E+02	6.65652E+02	6.84232E+02
	Std	5.53981E+00	3.61961E+00	**3.46968E+00**	3.96295E+00	4.19544E+00	4.76282E+00	7.10459E+00
	Avg	**6.56347E+02**	6.88249E+02	7.12495E+02	6.70057E+02	7.11764E+02	6.75097E+02	6.96166E+02
	Median	**6.57085E+02**	6.87158E+02	7.13575E+02	6.69121E+02	7.11761E+02	6.74756E+02	6.94573E+02
	Worse	**6.67116E+02**	6.97825E+02	7.17340E+02	6.82912E+02	7.19904E+02	6.86354E+02	7.09337E+02
F7	Min	**1.95929E+03**	3.30133E+03	3.90825E+03	2.51093E+03	3.75490E+03	2.54748E+03	2.94962E+03
	Std	2.63228E+02	**1.35956E+02**	5.50359E+01	1.69734E+02	9.65610E+01	2.00939E+02	1.45926E+02
	Avg	**2.50525E+03**	3.57924E+03	4.03823E+03	2.83835E+03	4.04413E+03	3.23121E+03	3.20779E+03
	Median	**2.51682E+03**	3.56150E+03	4.03739E+03	2.82409E+03	4.05718E+03	3.25070E+03	3.19596E+03
	Worse	**3.16520E+03**	3.91595E+03	4.12196E+03	3.17417E+03	4.16528E+03	3.51044E+03	3.53011E+03
F8	Min	**1.50319E+03**	2.08153E+03	2.53456E+03	1.66442E+03	2.42719E+03	1.69044E+03	2.05622E+03
	Std	1.04072E+02	**3.72339E+01**	4.14956E+01	8.31421E+01	5.85462E+01	1.21805E+02	7.36495E+01
	Avg	**1.68947E+03**	2.17295E+03	2.61265E+03	1.85578E+03	2.59002E+03	1.93341E+03	2.17860E+03
	Median	**1.67696E+03**	2.16392E+03	2.60974E+03	1.86984E+03	2.59051E+03	1.92321E+03	2.18063E+03
	Worse	**1.91533E+03**	2.24886E+03	2.67898E+03	1.99370E+03	2.71045E+03	2.14498E+03	2.35134E+03
F9	Min	**1.90650E+04**	4.38820E+04	7.17385E+04	3.34797E+04	6.52335E+04	2.43703E+04	4.73996E+04
	Std	1.96838E+04	6.36315E+03	**3.09721E+03**	1.21965E+04	4.93456E+03	4.14097E+03	8.28837E+03
	Avg	**3.64681E+04**	5.39261E+04	7.87389E+04	4.75591E+04	7.71083E+04	3.38440E+04	6.24624E+04
	Median	**2.32216E+04**	5.31377E+04	7.87904E+04	4.06958E+04	7.71023E+04	3.45190E+04	6.35228E+04
	Worse	7.35539E+04	6.88441E+04	8.38531E+04	6.81181E+04	8.42973E+04	**4.18294E+04**	7.55688E+04
F10	Min	**1.24742E+04**	1.85096E+04	3.09144E+04	1.82475E+04	3.06900E+04	1.58002E+04	2.94528E+04
	Std	5.63907E+03	1.95370E+03	**7.62376E+02**	3.75887E+03	8.93645E+02	1.89731E+03	8.26212E+02
	Avg	**1.80430E+04**	2.25915E+04	3.25113E+04	2.26163E+04	3.23193E+04	1.96932E+04	3.14889E+04
	Median	**1.58784E+04**	2.23058E+04	3.25179E+04	2.13069E+04	3.22705E+04	1.94987E+04	3.16288E+04
	Worse	3.09398E+04	**2.65097E+04**	3.37836E+04	3.20874E+04	3.40265E+04	2.33549E+04	3.27143E+04
F11	Min	**2.73116E+03**	2.55618E+04	1.73012E+05	4.84203E+04	1.80978E+05	2.89741E+04	8.10430E+04
	Std	**2.02298E+03**	7.71413E+03	5.64751E+04	1.81490E+04	5.08978E+04	1.08693E+04	2.64230E+04
	Avg	**4.20428E+03**	3.82697E+04	2.52748E+05	8.04337E+04	2.69105E+05	4.90882E+04	1.41621E+05
	Median	**3.57779E+03**	3.92433E+04	2.43042E+05	8.10697E+04	2.63662E+05	4.78495E+04	1.40764E+05
	Worse	**1.39539E+04**	5.64724E+04	4.01205E+05	1.29886E+05	3.61741E+05	7.63755E+04	2.09356E+05
F12	Min	**5.38968E+07**	2.27587E+09	1.77432E+11	2.73748E+10	1.84131E+11	1.69987E+09	1.22261E+10
	Std	**2.89922E+08**	5.86520E+09	1.46895E+10	9.65650E+09	1.41835E+10	7.55758E+09	6.99340E+09
	Avg	**4.07630E+08**	7.50934E+09	2.06903E+11	4.14307E+10	2.12666E+11	1.26667E+10	2.48848E+10
	Median	**3.31509E+08**	5.31866E+09	2.09061E+11	3.91421E+10	2.12528E+11	1.14023E+10	2.34329E+10
	Worse	**1.33198E+09**	2.90167E+10	2.29230E+11	5.90864E+10	2.35126E+11	3.58573E+10	4.06785E+10
F13	Min	**3.37022E+04**	5.85981E+08	3.94741E+10	1.38475E+09	3.52583E+10	1.35871E+08	3.78789E+08
	Std	**9.33848E+04**	2.03739E+09	5.04996E+09	3.67416E+09	6.22599E+09	1.23940E+09	2.24060E+09
	Avg	**9.10710E+04**	2.12982E+09	5.00754E+10	7.67647E+09	4.94297E+10	1.75402E+09	3.27668E+09
	Median	**6.55325E+04**	1.43948E+09	4.98625E+10	6.61359E+09	4.98448E+10	1.57560E+09	2.69985E+09
	Worse	**5.48668E+05**	1.12213E+10	5.66395E+10	1.62436E+10	5.83235E+10	4.69267E+09	9.92132E+09
F14	Min	**7.53467E+04**	1.58728E+06	4.14447E+07	1.76457E+06	3.38183E+07	9.59568E+05	5.39774E+06
	Std	**1.73144E+05**	2.00358E+06	3.94781E+07	6.58256E+06	4.46247E+07	2.90336E+06	5.70446E+06
	Avg	**2.28384E+05**	6.18820E+06	9.70322E+07	1.18178E+07	1.09034E+08	4.82349E+06	1.22054E+07
	Median	**1.78136E+05**	6.15279E+06	8.55168E+07	1.13315E+07	9.70351E+07	3.85266E+06	9.93777E+06
	Worse	**7.35167E+05**	1.04283E+07	1.83238E+08	2.88967E+07	1.89643E+08	9.98635E+06	2.64084E+07
F15	Min	**4.53162E+03**	4.09149E+08	1.85570E+10	1.57972E+08	1.47537E+10	8.76422E+05	1.07911E+08
	Std	**2.70166E+07**	7.48595E+08	4.02413E+09	2.25990E+09	4.88921E+09	4.85018E+08	4.66505E+08
	Avg	**4.95263E+06**	1.28595E+09	2.69433E+10	2.75869E+09	2.57760E+10	2.83343E+08	6.04134E+08
	Median	**1.49991E+04**	1.05653E+09	2.73173E+10	2.10756E+09	2.55724E+10	7.21503E+07	4.78274E+08
	Worse	**1.47996E+08**	3.00185E+09	3.52328E+10	8.60864E+09	3.42015E+10	2.14120E+09	1.89477E+09
F16	Min	**4.83554E+03**	8.98950E+03	1.77796E+04	7.38482E+03	1.86830E+04	5.77310E+03	8.78309E+03
	Std	**7.62392E+02**	1.94769E+03	3.37821E+03	1.09913E+03	3.97414E+03	1.12709E+03	1.22603E+03
	Avg	**6.25402E+03**	1.20502E+04	2.52585E+04	8.90643E+03	2.56128E+04	8.70587E+03	1.22535E+04
	Median	**6.09468E+03**	1.20996E+04	2.59335E+04	8.95981E+03	2.58203E+04	8.67147E+03	1.23418E+04
	Worse	**8.14081E+03**	1.70348E+04	3.10816E+04	1.16712E+04	3.51437E+04	1.06772E+04	1.48669E+04
F17	Min	**5.02150E+03**	6.48201E+03	1.21232E+06	6.38935E+03	4.84387E+05	5.60775E+03	7.19407E+03
	Std	**9.11970E+02**	3.89664E+03	1.32655E+07	1.66801E+04	1.48727E+07	8.54417E+03	4.46579E+04
	Avg	**6.67579E+03**	1.09552E+04	1.38934E+07	1.54292E+04	1.25155E+07	1.00136E+04	2.28839E+04
	Median	**6.55344E+03**	1.00427E+04	1.14083E+07	9.17541E+03	5.78528E+06	7.66763E+03	1.09633E+04
	Worse	**8.31308E+03**	2.05082E+04	6.42608E+07	7.08919E+04	4.76222E+07	4.83856E+04	2.53610E+05
F18	Min	**2.95187E+05**	2.27874E+06	1.13172E+08	1.92283E+06	3.68657E+07	1.32051E+06	5.32196E+06
	Std	**6.56451E+05**	2.65105E+06	1.27672E+08	9.82319E+06	1.65865E+08	2.60336E+06	6.63726E+06
	Avg	**8.77199E+05**	5.96164E+06	2.93377E+08	1.18982E+07	3.09307E+08	4.88726E+06	1.21565E+07
	Median	**6.63994E+05**	5.61937E+06	2.72969E+08	8.39722E+06	2.73351E+08	3.82754E+06	9.56705E+06
	Worse	**3.65853E+06**	1.35660E+07	5.92394E+08	4.44221E+07	6.59895E+08	1.33158E+07	2.80583E+07
F19	Min	**5.96235E+03**	9.24070E+07	1.87904E+10	9.91731E+07	1.50303E+10	9.34440E+06	7.36892E+07
	Std	**4.50432E+05**	1.16301E+09	4.00460E+09	1.44030E+09	5.01956E+09	7.19159E+08	5.57260E+08
	Avg	**3.02691E+05**	1.39653E+09	2.57454E+10	1.74685E+09	2.67066E+10	4.50620E+08	6.38277E+08
	Median	**7.04729E+04**	9.37464E+08	2.58878E+10	1.42133E+09	2.79132E+10	9.93487E+07	3.90427E+08
	Worse	**1.39291E+06**	4.47428E+09	3.42272E+10	5.26606E+09	3.34265E+10	3.06525E+09	2.43696E+09
F20	Min	4.34746E+03	5.14104E+03	6.17971E+03	**4.10575E+03**	6.76248E+03	4.68434E+03	6.82080E+03
	Std	8.02556E+02	**2.27572E+02**	4.61081E+02	8.16809E+02	3.57433E+02	5.43470E+02	3.13115E+02
	Avg	**5.42990E+03**	5.59108E+03	7.94111E+03	5.50757E+03	7.64490E+03	5.82785E+03	7.42170E+03
	Median	**5.24475E+03**	5.55104E+03	8.00899E+03	5.27906E+03	7.64609E+03	5.83958E+03	7.45975E+03
	Worse	6.82221E+03	**5.98035E+03**	8.56906E+03	7.23991E+03	8.19227E+03	6.92984E+03	8.07473E+03
F21	Min	**2.99497E+03**	3.13359E+03	4.55024E+03	3.16813E+03	4.48666E+03	3.22290E+03	3.92606E+03
	Std	1.16427E+02	**6.92636E+01**	2.13956E+02	1.37877E+02	2.01218E+02	1.86852E+02	1.78005E+02
	Avg	**3.23498E+03**	3.26201E+03	5.04169E+03	3.45303E+03	4.89481E+03	3.56783E+03	4.31836E+03
	Median	**3.23202E+03**	3.24886E+03	5.06964E+03	3.46071E+03	4.88247E+03	3.59508E+03	4.33308E+03
	Worse	3.50803E+03	**3.40622E+03**	5.46194E+03	3.73679E+03	5.31708E+03	3.86648E+03	4.63954E+03
F22	Min	**1.64820E+04**	2.34621E+04	3.37766E+04	1.97652E+04	3.31742E+04	1.80188E+04	2.50144E+04
	Std	1.56226E+03	9.99439E+02	**6.55742E+02**	3.18478E+03	7.54621E+02	1.95217E+03	2.11478E+03
	Avg	**1.96677E+04**	2.56059E+04	3.50748E+04	2.42439E+04	3.49791E+04	2.31311E+04	3.29419E+04
	Median	**2.00113E+04**	2.55145E+04	3.50823E+04	2.40222E+04	3.50298E+04	2.37538E+04	3.36646E+04
	Worse	**2.41861E+04**	2.76461E+04	3.62728E+04	3.38675E+04	3.62645E+04	2.68493E+04	3.49254E+04
F23	Min	3.75815E+03	**3.64527E+03**	6.06491E+03	4.02175E+03	6.44350E+03	4.03568E+03	4.95518E+03
	Std	1.71235E+02	**7.52393E+01**	3.51872E+02	1.71274E+02	5.17863E+02	1.66488E+02	1.55258E+02
	Avg	4.01332E+03	**3.85963E+03**	6.75814E+03	4.32005E+03	7.73700E+03	4.32065E+03	5.31372E+03
	Median	4.00036E+03	**3.86513E+03**	6.83526E+03	4.29219E+03	7.88828E+03	4.32317E+03	5.36873E+03
	Worse	4.35947E+03	**3.98919E+03**	7.34649E+03	4.71587E+03	8.39997E+03	4.69815E+03	5.53322E+03
F24	Min	4.30912E+03	**4.26097E+03**	8.78563E+03	4.99951E+03	1.08648E+04	4.76779E+03	5.79593E+03
	Std	**5.27490E+02**	9.10418E+01	1.02108E+03	3.01046E+02	9.30127E+02	2.62822E+02	3.65375E+02
	Avg	5.18117E+03	**4.42375E+03**	1.03380E+04	5.78472E+03	1.28902E+04	5.29495E+03	6.75888E+03
	Median	5.14068E+03	**4.41106E+03**	1.02728E+04	5.82522E+03	1.29292E+04	5.26682E+03	6.80308E+03
	Worse	6.46276E+03	**4.64150E+03**	1.29779E+04	6.38044E+03	1.40970E+04	5.78317E+03	7.48302E+03
F25	Min	**3.18808E+03**	4.92200E+03	2.55924E+04	8.48115E+03	2.69286E+04	5.43706E+03	7.14862E+03
	Std	**5.43737E+01**	6.77323E+02	1.82252E+03	1.49565E+03	1.96598E+03	1.18787E+03	1.56714E+03
	Avg	**3.30895E+03**	6.02390E+03	2.97377E+04	1.14331E+04	3.02220E+04	7.48772E+03	9.91014E+03
	Median	**3.30627E+03**	5.91212E+03	2.96329E+04	1.12090E+04	3.06036E+04	7.30310E+03	9.95206E+03
	Worse	**3.40002E+03**	7.79740E+03	3.34490E+04	1.49457E+04	3.32637E+04	1.06144E+04	1.27985E+04
F26	Min	1.41986E+04	**1.40033E+04**	4.85760E+04	2.15033E+04	4.83545E+04	1.72241E+04	2.86704E+04
	Std	3.10099E+03	2.99213E+03	2.63876E+03	**1.99253E+03**	2.52469E+03	3.85124E+03	3.68736E+03
	Avg	**2.05848E+04**	2.49651E+04	5.40678E+04	2.56821E+04	5.38489E+04	2.83879E+04	3.44589E+04
	Median	**2.02473E+04**	2.58684E+04	5.46272E+04	2.55970E+04	5.43527E+04	2.96505E+04	3.52693E+04
	Worse	**2.62633E+04**	2.87881E+04	5.81811E+04	2.97521E+04	5.75033E+04	3.39865E+04	4.34091E+04
F27	Min	**3.57075E+03**	3.96221E+03	1.11418E+04	4.49189E+03	1.26547E+04	4.22571E+03	4.37232E+03
	Std	1.67305E+02	**1.44623E+02**	1.95051E+03	3.52146E+02	1.57613E+03	4.84011E+02	7.85366E+02
	Avg	**3.83615E+03**	4.17468E+03	1.42763E+04	5.46391E+03	1.54758E+04	5.00542E+03	5.46874E+03
	Median	**3.79845E+03**	4.15762E+03	1.40409E+04	5.44479E+03	1.56545E+04	4.96060E+03	5.28731E+03
	Worse	**4.18402E+03**	4.45646E+03	1.80233E+04	6.03234E+03	1.86490E+04	6.69349E+03	7.68370E+03
F28	Min	**3.31149E+03**	6.04841E+03	2.45295E+04	1.07837E+04	2.55767E+04	5.63667E+03	8.00971E+03
	Std	**4.04602E+01**	6.67291E+02	1.63175E+03	1.96157E+03	1.85083E+03	1.62458E+03	1.89166E+03
	Avg	**3.39945E+03**	7.15306E+03	3.05321E+04	1.55787E+04	3.02897E+04	9.46397E+03	1.22824E+04
	Median	**3.39970E+03**	7.06491E+03	3.10465E+04	1.54258E+04	3.06092E+04	9.56246E+03	1.21650E+04
	Worse	**3.48254E+03**	8.76759E+03	3.24903E+04	2.01825E+04	3.30738E+04	1.30552E+04	1.72896E+04
F29	Min	**6.58371E+03**	1.02863E+04	2.59689E+05	9.08939E+03	8.03833E+04	9.50069E+03	1.36857E+04
	Std	**6.40252E+02**	2.85518E+03	4.94060E+05	5.40864E+03	5.57382E+05	1.72523E+03	2.80588E+03
	Avg	**7.74144E+03**	1.32332E+04	8.36672E+05	1.42736E+04	8.25401E+05	1.24938E+04	1.72928E+04
	Median	**7.55464E+03**	1.24372E+04	7.11695E+05	1.32898E+04	7.97681E+05	1.24605E+04	1.67808E+04
	Worse	**8.88382E+03**	2.22457E+04	2.01910E+06	4.06404E+04	2.22609E+06	1.55279E+04	2.38589E+04
F30	Min	**2.77206E+05**	1.99819E+08	2.99125E+10	2.11789E+09	2.02008E+10	1.68900E+08	6.67972E+08
	Std	**2.72941E+07**	2.61068E+08	5.66182E+09	2.35451E+09	8.93925E+09	1.75603E+09	1.06345E+09
	Avg	**1.46562E+07**	5.39235E+08	4.16818E+10	6.23646E+09	4.26576E+10	2.03929E+09	2.54632E+09
	Median	**3.08058E+06**	4.71885E+08	4.16521E+10	6.37761E+09	4.24835E+10	1.44950E+09	2.35684E+09
	Worse	**1.35238E+08**	1.29488E+09	5.11261E+10	1.05314E+10	5.54581E+10	6.35518E+09	4.85901E+09
Friedman	Value	1.10345E+00	2.93103E+00	6.55172E+00	3.75862E+00	6.44828E+00	2.68966E+00	4.51724E+00
	Rank	1	3	7	4	6	2	5

Significant values are in bold.

Friedman's overall order is TNTWCOA > SCSO > ICOA > GJO > SABO > OOA > COA. Therefore, from the statistical results of evaluation index, when Dim = 50, the algorithm proposed in this paper shows excellent performance compared with other five algorithms. Compared with the original COA and ICOA algorithm, the statistical results of evaluation index of the improved algorithm have been significantly improved.

#### Convergence analysis

**Fig. 10:**
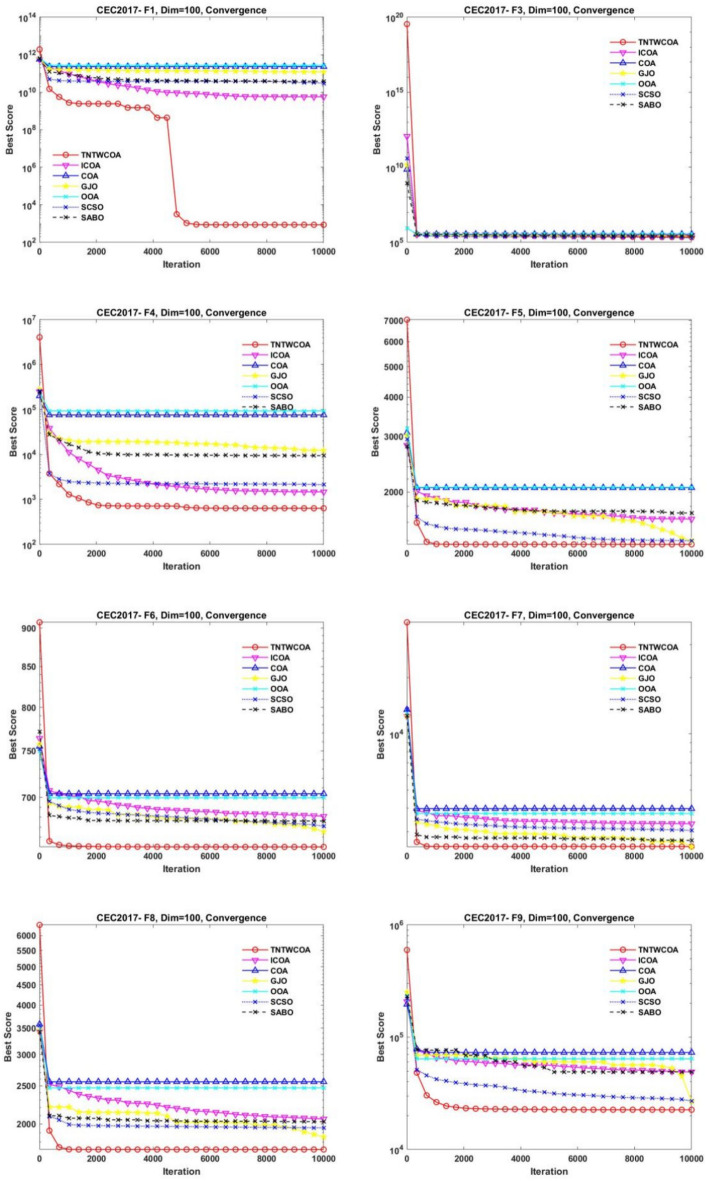

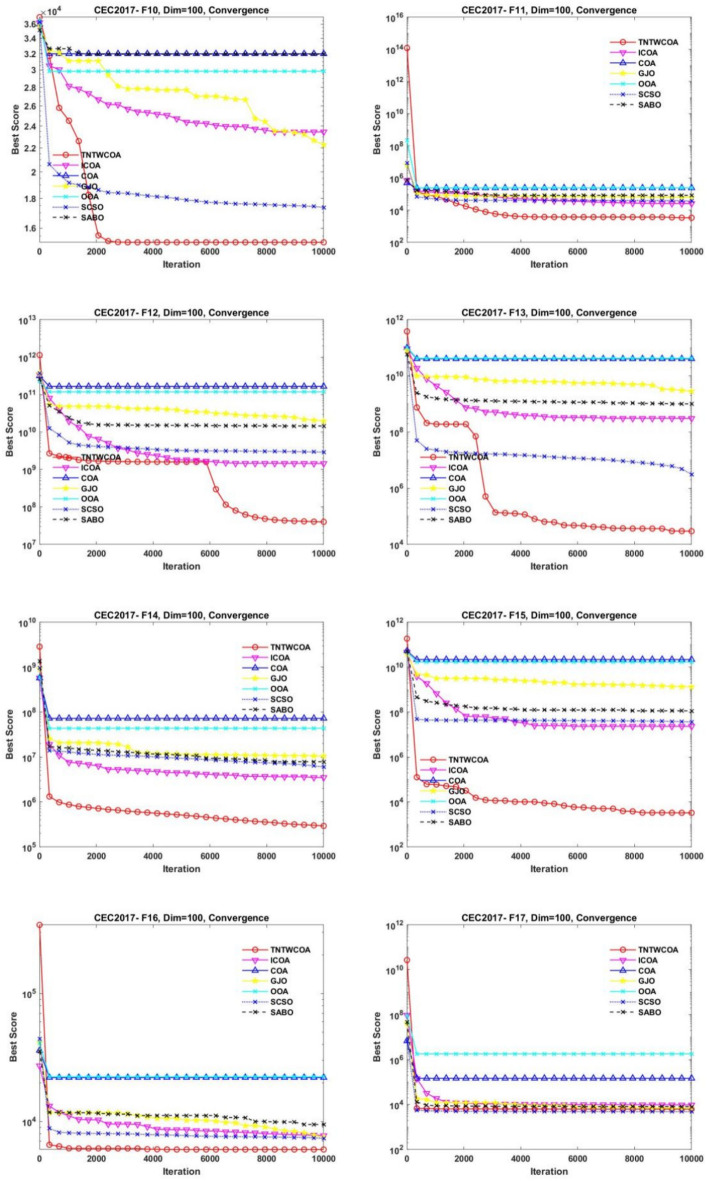

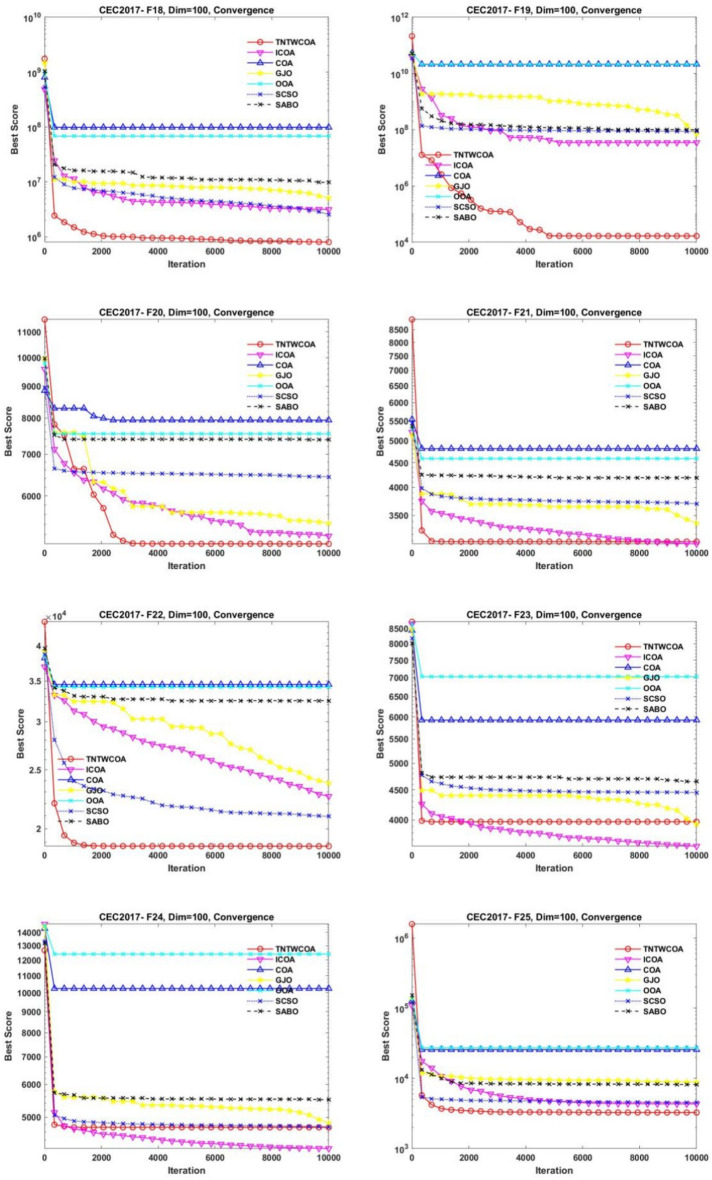

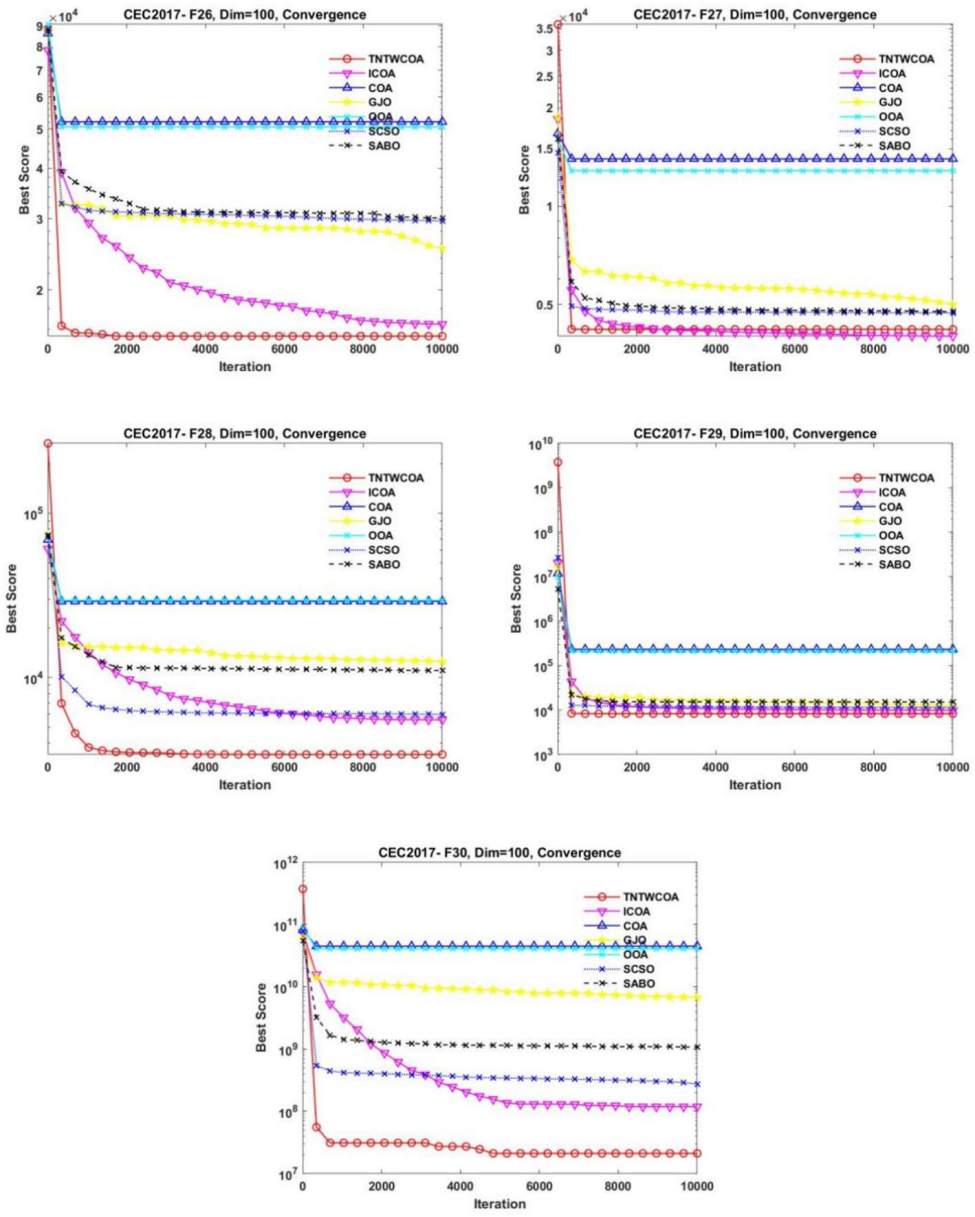
The convergence curves of the proposed technique and other five algorithms for IEEE CEC2017 benchmark functions (Dim = 100).

Figure 10 illustrates the convergence curves of GJO, SCSO, OOA, SABO, original COA, ICOA and TNTWCOA on 29 benchmark functions of the IEEE CEC2017 throughout the iterations with 10000times.

As can be seen from Fig. 10, except F3, F17 and F24, the convergence speed of the improved TNTWCOA algorithm is optimal. Among them, when the optimization function F4, F5, F6, F7, F8, F9, F10, F11, F14, F15, F16, F18, F21, F22, F25, F26, F28, F29, F30, it can quickly converge to the best value and maintain the stability. When optimizing the functions F1, F12, F19, F20, it can quickly converge to the best value and will continue to optimize to make the best better. When optimizing functions F23, they can quickly converge to the best value and remain stable, but the best value will be surpassed by ICOA and GJO in the later period, and F27 will be surpassed by ICOA. When optimizing functions F13, it can not quickly converge to the best value, but it will gradually converge to the best and better.

#### Analysis of box plot results

**Fig. 11:**
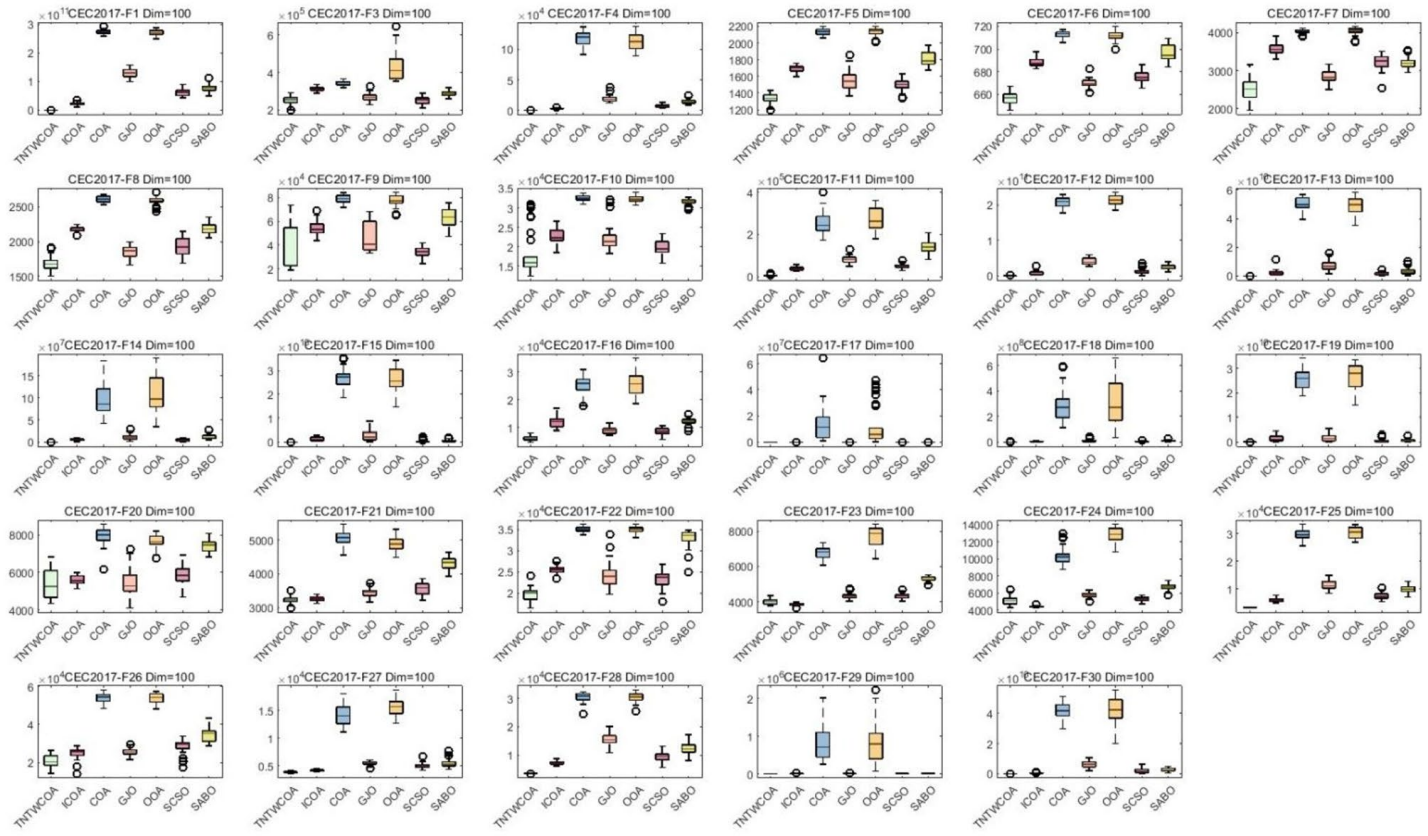
Boxplots for the proposed technique and other five algorithms for IEEE CEC2017 benchmark functions (Dim = 100).

The largest number (maximum value) and the smallest number (minimum value) in Fig. 9 constitute the variation range of the optimal values of 29 functions optimized by GJO, SCSO, OOA, SABO, COA, and ICOA algorithms in CEC 2017 after running 30 times. That is, the narrower the box graph, the smaller the fluctuation range of the optimal value of the function running 30 times, and the more stable the optimization; The lower the position of the box diagram, the smaller the function optimization value and the closer it is to the theoretical value. The "o" in the diagram indicates the existence of singularity. As can be seen from Fig. 11, except for F23 and F 24, the other have the lower the position of the box diagram, when optimized F9, F20, the narrow of the box diagram is worst, when optimized F1, F3, F11, F12, F13, F14, F15, F16, F17, F19, F25, F27, F28, F29, F30, they have the smallest number (minimum value), the first quartile (25% point value) of the function box diagram; Middle digit (median value); The third quartile (75% locus value); The largest numbers (maximum) almost overlap.

#### Analysis of Wilcoxon rank sum test results

Table 7 shows the Wilcoxon rank sum test results (Dim = 100). As can be seen from Table 7. There is a significant deviation between TNTWCOA and the function optimization results of most algorithms. However, when optimizing functions F20, the Wilcoxon rank sum test of TNTWCOA algorithm and GJO and **ICOA** algorithm is greater than 0.05, indicating that the obtained function optimization values are not significantly different. At the same time, when optimizing function F21, the Wilcoxon rank sum test of TNTWCOA algorithm and **ICOA** algorithm is greater than 0.05, indicating that there is no significant difference between the obtained function optimization values. F24, the Wilcoxon rank sum test of TNTWCOA algorithm and **SCSO** algorithm is greater than 0.05, indicating that there is no significant difference between the obtained function optimization values.

**Table 7:** Wilcoxon rank sum test results (Dim = 100).

	ICOA	COA	GJO	OOA	SCSO	SABO
F1	3.01986E−11	3.01986E−11	3.01986E−11	3.01986E−11	3.01986E−11	3.01986E−11
F3	3.33839E−11	3.01986E−11	1.56381E−02	3.01986E−11	9.94102E−01	2.38974E−08
F4	3.01986E−11	3.01986E−11	3.01986E−11	3.01986E−11	3.01986E−11	3.01986E−11
F5	3.01986E−11	3.01986E−11	2.37147E−10	3.01986E−11	8.10136E−10	3.01986E−11
F6	3.01986E−11	3.01986E−11	7.38908E−11	3.01986E−11	3.68973E−11	3.01986E−11
F7	3.01986E−11	3.01986E−11	1.02773E−06	3.01986E−11	2.37147E−10	1.09367E−10
F8	3.01986E−11	3.01986E−11	4.11271E−07	3.01986E−11	1.42942E−08	3.01986E−11
F9	2.15664E−03	4.50432E−11	2.37996E−03	1.77691E−10	4.67558E−02	1.09069E−05
F10	6.35604E−05	3.33839E−11	4.63897E−05	3.68973E−11	7.29511E−04	8.99341E−11
F11	3.01986E−11	3.01986E−11	3.01986E−11	3.01986E−11	3.01986E−11	3.01986E−11
F12	3.01986E−11	3.01986E−11	3.01986E−11	3.01986E−11	3.01986E−11	3.01986E−11
F13	3.01986E−11	3.01986E−11	3.01986E−11	3.01986E−11	3.01986E−11	3.01986E−11
F14	3.01986E−11	3.01986E−11	3.01986E−11	3.01986E−11	3.01986E−11	3.01986E−11
F15	3.01986E−11	3.01986E−11	3.01986E−11	3.01986E−11	1.95678E−10	4.07716E−11
F16	3.01986E−11	3.01986E−11	8.15274E−11	3.01986E−11	8.10136E−10	3.01986E−11
F17	2.19474E−08	3.01986E−11	7.04298E−07	3.01986E−11	4.85602E−03	6.12104E−10
F18	5.49405E−11	3.01986E−11	5.49405E−11	3.01986E−11	1.95678E−10	3.01986E−11
F19	3.01986E−11	3.01986E−11	3.01986E−11	3.01986E−11	3.01986E−11	3.01986E−11
F20	**2.39850E−01**	6.06576E−11	**5.39510E−01**	3.33839E−11	4.05950E−02	3.33839E−11
F21	**1.90730E−01**	3.01986E−11	3.01026E−07	3.01986E−11	2.01522E−08	3.01986E−11
F22	3.68973E−11	3.01986E−11	5.09220E−08	3.01986E−11	5.09220E−08	3.01986E−11
F23	2.83887E−04	3.01986E−11	2.78287E−07	3.01986E−11	1.35943E−07	3.01986E−11
F24	5.46175E−09	3.01986E−11	8.88288E−06	3.01986E−11	**1.37323E−01**	8.99341E−11
F25	3.01986E−11	3.01986E−11	3.01986E−11	3.01986E−11	3.01986E−11	3.01986E−11
F26	1.49180E−06	3.01986E−11	3.08105E−08	3.01986E−11	1.69795E−08	3.01986E−11
F27	2.38974E−08	3.01986E−11	3.01986E−11	3.01986E−11	3.01986E−11	3.01986E−11
F28	3.01986E−11	3.01986E−11	3.01986E−11	3.01986E−11	3.01986E−11	3.01986E−11
F29	3.01986E−11	3.01986E−11	3.01986E−11	3.01986E−11	3.01986E−11	3.01986E−11
F30	3.01986E−11	3.01986E−11	3.01986E−11	3.01986E−11	3.01986E−11	3.01986E−11

Significant values are in bold.

## TNTWCOA for engineering optimization problems

To verify the actual optimization effect of TNTWCOA in solving engineering problems, the optimization performance of TNTWCOA is tested by using the selected four classic engineering problems, and the specific performance of the TNTWCOA on each engineering problem is as follows:

### Three‑bar truss design problem6

**Fig. 12:**
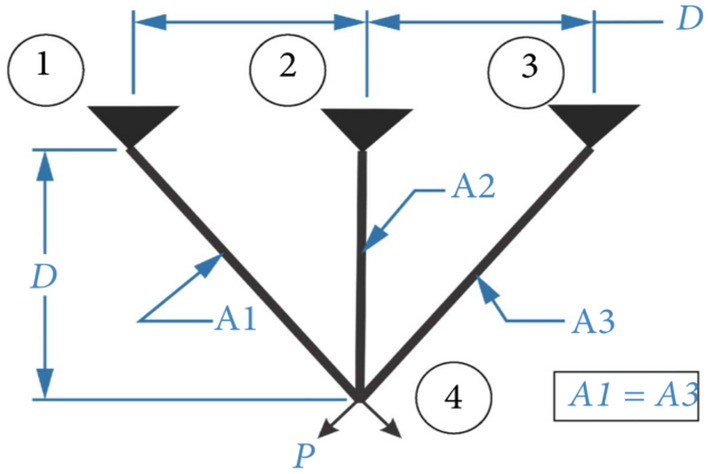
Schematic of three-bar truss problem.

The main purpose of studying the design of a three-bar truss is to reduce the structure’s weight under the action of the total sup- porting load p. The geometry of this problem is given in Fig. 12. In the benchmark suite, the total number of decision variables $D = 2$, the number of inequality constraints $g = 3$, the number of equality constraints $h = 0$, and the best known feasible objective function value $f(x*) = 2.6389584338E + 02$^24,25^.

The mathematical formula of the three-bar truss problem is as follows:

- Consider: $$\vec{x} = \left[ {\begin{array}{*{20}l} {x_{1} } \hfill & {x_{2} } \hfill \\ \end{array} } \right] = \left[ {\begin{array}{*{20}l} {A_{1} } \hfill & {A_{2} } \hfill \\ \end{array} } \right]$$
- Object : $$Minimize\;f\left( {\vec{x}} \right) = \left( {2\sqrt 2 x_{1} + x_{2} } \right)*l$$
- Subject to: $$g_{1} \left( {\vec{x}} \right) = \frac{{\sqrt 2 x_{1} + x_{2} }}{{\sqrt 2 x_{1}^{2} + 2x_{1} x_{2} }}P - \sigma \le 0$$, $$g_{2} \left( {\vec{x}} \right) = \frac{{x_{2} }}{{\sqrt 2 x_{1}^{2} + 2x_{1} x_{2} }}P - \sigma \le 0$$, $$g_{3} \left( {\vec{x}} \right) = \frac{{x_{1} }}{{\sqrt 2 x_{2} + x_{1} }}P - \sigma \le 0$$
- Variables range: $$0 \le x_{1} ,x_{2} \le 1$$
- Where: $$l = 100,\;P = 2\frac{{{\text{kN}}}}{{{\text{cm}}^{2} }},\;\sigma = 2\frac{{{\text{kN}}^{2} }}{{{\text{cm}}^{2} }}$$

**Fig. 13:**
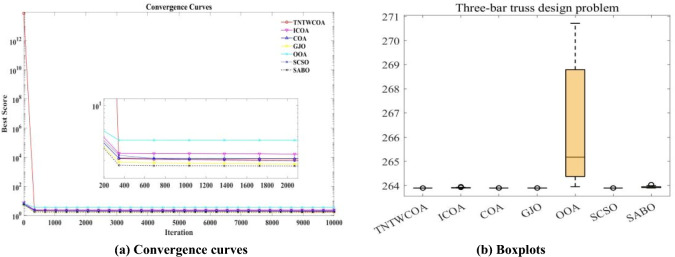
The convergence curves and boxplots of the proposed and others five algorithms for three-bar truss problem.

The iterative process of finding the optimal solution of the six algorithms is shown in Fig. 13a, and its box-plots is shown in Fig. 13b. What is clear from the simulation results is that TNTWCOA has fast provided the optimal solution to the three-bar truss problem and the objective function value equal to **2.63896E+02**. The statistical results obtained from TNTWCOA and competitor algorithms implementation are released in Table 8. These results show that TNTWCOA has superior performance over competitor algorithms due to better values of statistical indicators.

**Table 8:** Statistical results for the three-bar truss problem. Bold values have the best performance.

Item	TNTWCOA	ICOA	COA	GJO	OOA	SCSO	SABO
Min	**2.63896E+02**	2.63897E+02	**2.63896E+02**	**2.63896E+02**	2.63952E+02	**2.63896E+02**	2.63899E+02
Std	**8.61860E−06**	1.35902E−02	9.11074E−04	5.71830E−04	2.47965E+00	3.38133E−04	3.03910E−02
Avg	**2.63896E+02**	2.63909E+02	**2.63896E+02**	2.63897E+02	2.66483E+02	**2.63896E+02**	2.63937E+02
Median	**2.63896E+02**	2.63903E+02	**2.63896E+02**	**2.63896E+02**	2.65179E+02	**2.63896E+02**	2.63930E+02
Worse	**2.63896E+02**	2.63948E+02	2.63900E+02	2.63898E+02	2.70714E+02	2.63898E+02	2.64036E+02

Significant values are in bold.

### Welded beam design5

**Fig. 14:**
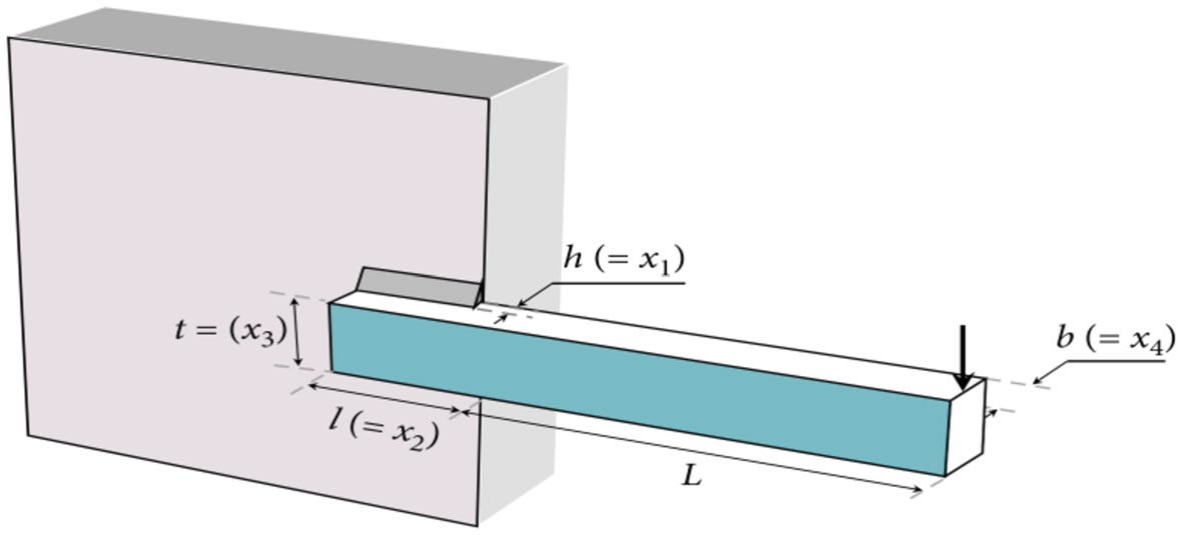
Schematic of welded beam design problem.

Welding beam design is a common and challenging problem in structural engineering. The goal is to achieve the best structural performance and minimize the weight of the beam by optimizing parameters such as the shape, size, and layout of the weld under given constraints. In the benchmark suite, the total number of decision variables $D = 4$, the number of inequality constraints $g = 5$, the number of equality constraints $h = 0$, and the best known feasible objective function value $f(x*) = 1.6702177263$^25,26^. The specific structure of the welded beam design is shown in Fig. 14.

The mathematical formula of the Welded beam design is as follows:

- Consider: $$\vec{x} = \left[ {\begin{array}{*{20}c} {x_{1} } & {x_{2} } & {x_{3} } & {x_{4} } \\ \end{array} } \right] = \left[ {\begin{array}{*{20}c} h & l & t & b \\ \end{array} } \right]$$
- Object: $$Minimize\;f\left( {\vec{x}} \right) = 1.10471x_{1}^{2} x_{2} + 0.04811x_{3} x_{4} \left( {14.0 + x_{2} } \right)$$
- Subject to: $$g_{1} \left( {\vec{x}} \right) = x_{1} - x_{4} \le 0$$, $$g_{2} \left( {\vec{x}} \right) = \sigma \left( {\vec{x}} \right) - \sigma_{{{\text{max}}}} \le 0$$, $$g_{3} \left( {\vec{x}} \right) = \delta \left( {\vec{x}} \right) - \delta_{{{\text{max}}}} \le 0$$, $$g_{4} \left( {\vec{x}} \right) = \tau \left( {\vec{x}} \right) - \tau_{{{\text{max}}}} \le 0$$,$$g_{7} \left( {\vec{x}} \right) = 1.10471x_{1}^{2} + 0.04811x_{3} x_{4} \left( {14.0 + x_{2} } \right) - 5.0 \le 0$$
- Variables range: $$0.1 \le x_{1}$$,$$x_{4} \le 2$$,$$0.1 \le x_{2}$$,$$x_{3} \le 10$$
- Where: $$  \tau \left( {\vec{x}} \right) = \sqrt {\left( {r^{\prime}} \right)^{2}  + 2\tau ^{\prime}r^{\prime\prime}\frac{{x_{2} }}{{2R}} + \left( {r^{\prime\prime}} \right)^{2} }   $$,$$\tau^{\prime} = \frac{p}{{\sqrt 2 x_{1} x_{2} }}$$,$$\tau^{*} = \frac{MR}{J}$$,$$M = p\left( {L + \frac{{x_{2} }}{2}} \right)$$,$$R = \sqrt {\frac{{x_{2}^{2} }}{4} + \left( {\frac{{x_{1} + x_{3} }}{2}} \right)^{2} }$$,$$J = 2\left\{ {\sqrt 2 x_{1} x_{2} \left[ {\frac{{x_{2}^{2} }}{4} + \left( {\frac{{x_{1} + x_{3} }}{2}} \right)^{2} } \right]} \right\}$$,$$\sigma \left( {\vec{x}} \right) = \frac{6PL}{{x_{4} x_{3}^{2} }}$$,$$\delta \left( {\vec{x}} \right) = \frac{{6PL^{3} }}{{Ex_{3}^{2} x_{4} }}$$,$$P_{c} \left( {\vec{x}} \right) = \frac{{4.013E\sqrt {x_{3}^{2} x_{4}^{6} /36} }}{{L^{2} }}\left( {1 - \frac{{x_{3} }}{2L}\sqrt{\frac{E}{4G}}  } \right)$$,$$P = 6000{\text{lb}}$$,$$L = 14{\text{in}}$$,$$\delta_{{{\text{max}}}} = 0.25{\text{in}}$$,$$\tau_{\max } = 13,600\;\;{\text{psi}}$$,$$\sigma_{\max } = 30,000\;\;{\text{psi}}$$

**Fig. 15:**
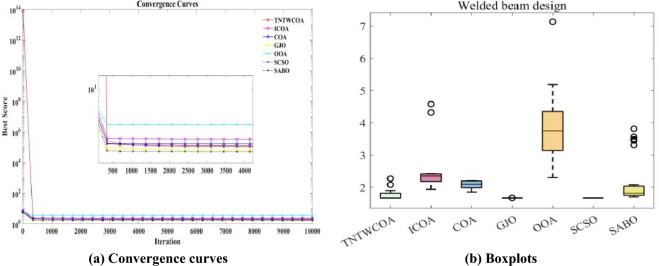
The convergence curves and boxplots of the proposed and others five algorithms for he Welded beam design problem.

The iterative process of finding the optimal solution of the six algorithms is shown in Fig. 15a, and its box-plots is shown in Fig. 15b, What is clear from the simulation results is that TNTWCOA has fast provided the optimal solution to the welded beam design problem and the objective function value equal to **1.67022E+00**. The statistical results obtained from TNTWCOA and competitor algorithms implementation are released in Table 9. These results show that TNTWCOA has superior performance over competitor algorithms due to better values of statistical indicators, except SCSO.

**Table 9:** Statistical results for the Welded beam design problem. Bold values have the best performance.

Item	TNTWCOA	ICOA	COA	GJO	OOA	SCSO	SABO
Min	**1.67022E+00**	1.93833E+00	1.85483E+00	1.67045E+00	2.30321E+00	1.67024E+00	1.69937E+00
Std	**1.37882E−01**	5.72194E−01	1.19553E−01	1.14312E−03	9.52159E−01	3.29042E−04	6.97906E−01
Avg	1.76066E+00	2.42431E+00	2.07140E+00	1.67121E+00	3.84793E+00	**1.67060E+00**	2.16555E+00
Median	**1.67030E+00**	2.35377E+00	2.09392E+00	1.67084E+00	3.75110E+00	1.67053E+00	1.81896E+00
Worse	2.27164E+00	4.57771E+00	2.20372E+00	1.67640E+00	7.13175E+00	**1.67142E+00**	3.81304E+00

Significant values are in bold.

### The gear train design problem

**Fig. 16:**
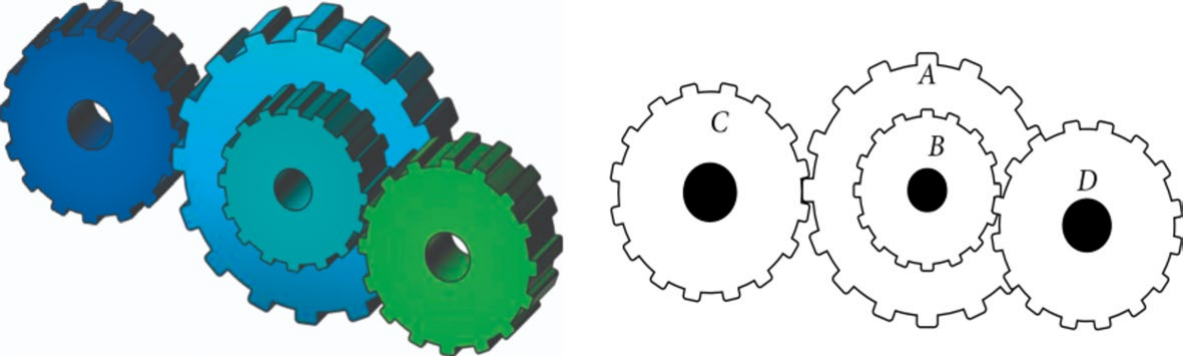
Schematic of gear train design problem.

The gear train design problem aims to minimize the transmission ratio. In the benchmark suite, the total number of decision variables $D = 4$, the number of inequality constraints ${\text{g}} = 1$, the number of equality constraints h = 1, and the best known feasible objective function value $f(x*) = 0$^25,27^. The gear train design problem structural diagram is shown in Fig. 16.

The mathematical formula of the gear train design problem is as follows:

- Consider:$$\vec{x} = \left[ {\begin{array}{*{20}c} {x_{1} } & {x_{2} } & {x_{3} } & {x_{4} } \\ \end{array} } \right] = \left[ {\begin{array}{*{20}c} {n_{A} } & {n_{B} } & {n_{C} } & {n_{D} } \\ \end{array} } \right]$$
- Object: $$Minimize\;f(\vec{x}) = \left( {\frac{1}{6.931} - \frac{{x_{1} x_{2} }}{{x_{3} x_{4} }}} \right)^{2} ,$$
- Variables range: $$x_{1} ,x_{2} ,x_{3} ,x_{4} \in \{ 12,13,14, \ldots ,60\} .$$

**Fig. 17:**
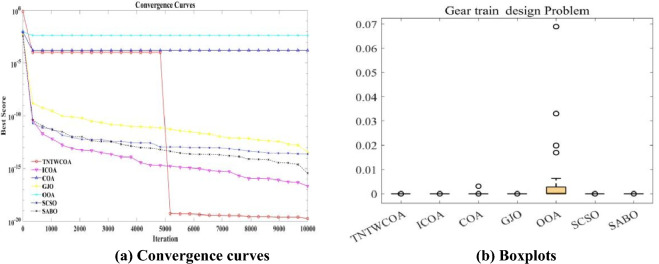
The convergence curves and box-plots of the proposed and others five algorithms for gear train design problem.

The iterative process of finding the optimal solution of the six algorithms is shown in Fig. 17a, and its box-plots is shown in Fig. 17b, What is clear from the simulation results is that TNTWCOA has fast provided the optimal solution to the gear train design problem and the objective function value equal to 0. The statistical results obtained from TNTWCOA and competitor algorithms implementation are released in Table 10. These results show that TNTWCOA has superior performance over ICOA, GJO, OOA, SCSO, SABO algorithms due to better values of statistical indicators.

**Table 10:** Statistical results for the gear train design problem. Bold values have the best performance.

Item	TNTWCOA	ICOA	COA	GJO	OOA	SCSO	SABO
Min	**0.00000E+00**	4.54686E−21	**0.00000E+00**	1.94012E−18	**0.00000E+00**	7.88883E−22	2.40950E−17
Std	**6.32615E−20**	4.29994E−17	5.63890E−04	5.77091E−14	1.40194E−02	2.82917E−16	3.53036E−14
Avg	**2.01331E−20**	2.31732E−17	1.02952E−04	3.46982E−14	5.55791E−03	1.33116E−16	2.57055E−14
Median	3.37950E−27	5.86525E−18	**0.00000E+00**	7.83143E−15	3.13632E−04	4.64701E−17	1.22154E−14
Worse	**2.91088E−19**	2.20899E−16	3.08855E−03	2.64577E−13	6.88869E−02	1.50203E−15	1.30405E−13

Significant values are in bold.

### Speed reducer design problem1

**Fig. 18:**
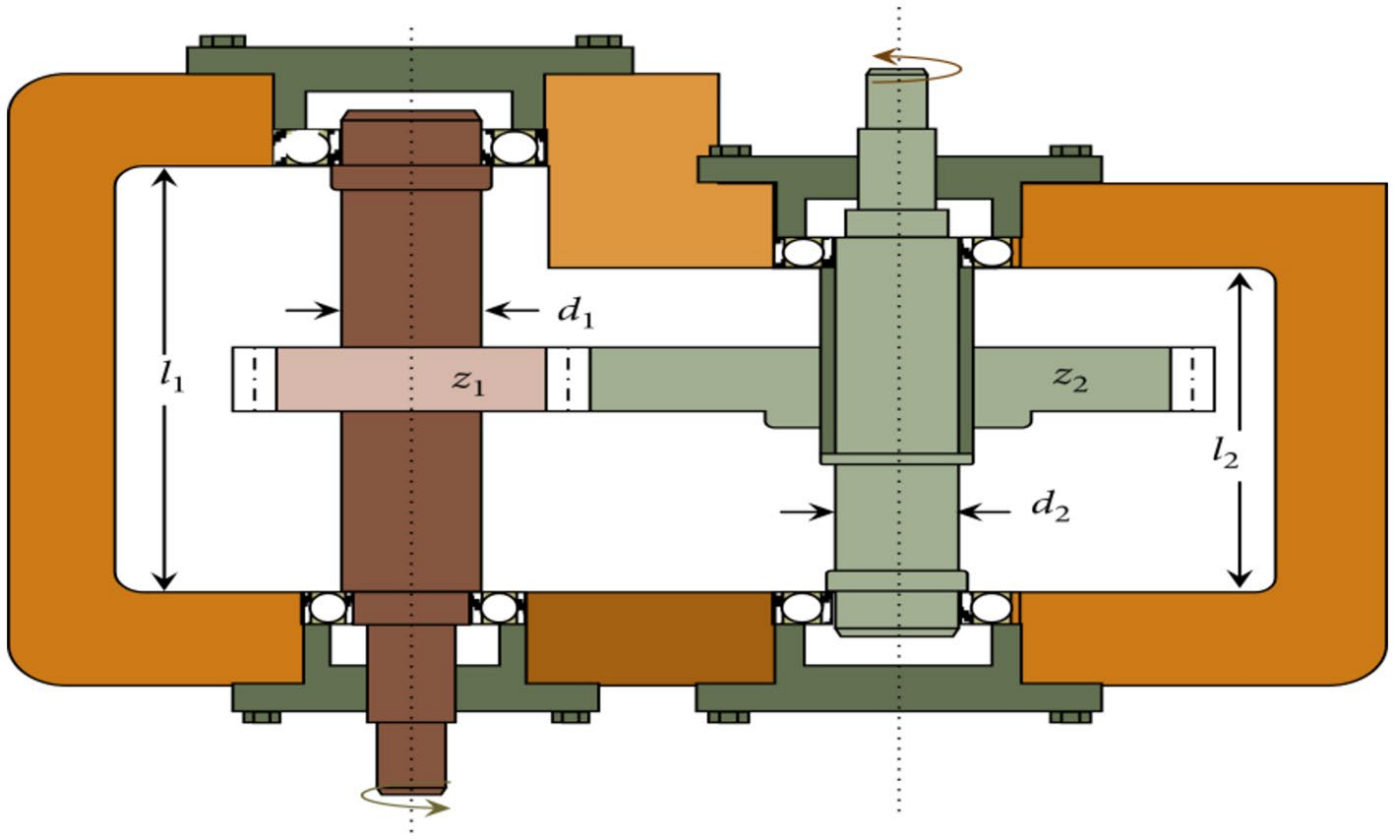
Schematic of speed reducer design problem.

Speed reducer design is an optimization challenge in engineering sciences. The goal is to minimize the weight of the speed reducer. The total number of decision variables $D = 7$, the number of inequality constraints g = 11, the number of equality constraints $h = 0$, and the best known feasible objective function value $f(x*) = 2.9944E + 03$^25,28^. The specific structure of the speed reducer design is shown in Fig. 18.

The mathematical formulation of the speed reducer design optimization problem is as follows:

- Consider:$$\vec{x} = \left[ {\begin{array}{*{20}c} {x_{1} } & {x_{2} } & {x_{3} } & {\begin{array}{*{20}c} {x_{4} } & {x_{5} } & {\begin{array}{*{20}c} {x_{6} } & {x_{7} } \\ \end{array} } \\ \end{array} } \\ \end{array} } \right] = \left[ {\begin{array}{*{20}c} b & m & {z_{2} } & {\begin{array}{*{20}c} {l_{1} } & {l_{2} } & {\begin{array}{*{20}c} {d_{1} } & {d_{2} } \\ \end{array} } \\ \end{array} } \\ \end{array} } \right]$$
- Object: $$\begin{gathered} Minimize\;f\left( X \right) = 0.7854x_{1} x_{2}^{2} \left( {3.3333x_{3}^{2} + 14.9334x_{3} - 43.0934} \right) \hfill \\ \;\;\;\;\;\;\;\;\;\;\;\;\;\;\;\;\;\;\;\;\;\;\;\; - 1.508x_{1} \left( {x_{6}^{2} + x_{7}^{2} } \right) + 7.4777\left( {x_{6}^{3} + x_{7}^{3} } \right) + 0.7854\left( {x_{4} x_{6}^{2} + x_{5} x_{7}^{2} } \right) \hfill \\ \end{gathered}$$
- Subject to: $$g_{1} \left( {\vec{x}} \right) = 27 - x_{1} x_{2}^{2} x_{3} \le 0$$,$$g_{2} \left( {\vec{x}} \right) = 397.5 - x_{1} x_{2}^{2} x_{3}^{2} \le 0$$, $$g_{3} \left( {\vec{x}} \right) = 1.93x_{4}^{3} - x_{2} x_{6}^{4} x_{3} \le 0$$,$$g_{4} \left( {\vec{x}} \right) = 1.93x_{5}^{3} - x_{2} x_{7}^{4} x_{3} \le 0$$, $$g_{5} \left( {\vec{x}} \right) = 10x_{6}^{ - 3} \sqrt {\left( {745x_{4} /x_{2} x_{3} } \right)^{2} + 16.9 \times 10^{6} } - 1100 \le 0$$, $$g_{6} \left( {\vec{x}} \right) = 10x_{7}^{ - 3} \sqrt {\left( {745x_{5} /x_{2} x_{3} } \right)^{2} + 157.5 \times 10^{6} } - 850 \le 0$$, $$g_{7} \left( {\vec{x}} \right) = x_{2} x_{3} - 40 \le 0$$,$$g_{8} \left( {\vec{x}} \right) = 5x_{2} - x_{1} \le 0$$,$$g_{9} \left( {\vec{x}} \right) = x_{1} - 12x_{2} \le 0$$, $$g_{10} \left( {\vec{x}} \right) = 1.5x_{6} + 1.9 - x_{4} \le 0$$,$$g_{11} \left( {\vec{x}} \right) = 1.1x_{7} + 1.9 - x_{5} \le 0$$
- Variables range:$$2.6 \le x_{1} \le 3.6$$,$$0.7 \le x_{2} \le 0.8$$,$$x_{3} \in \left\{ {17,18,19, \ldots ,28} \right\}$$,$$7.3 \le x_{4}$$,$$x_{5} \le 8.3$$,$$2.9 \le x_{6} \le 3.9$$,$$5 \le x_{7} \le 5.5$$

**Fig. 19:**
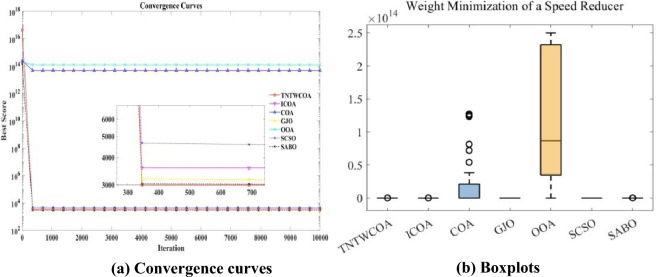
The convergence curves and boxplots of the proposed and others five algorithms for speed reducer design problem.

The iterative process of finding the optimal solution of the six algorithms is shown in Fig. 19a, and its box-plots is shown in Fig. 19b, what is clear from the simulation results is that TNTWCOA has fast provided the optimal solution to the speed reducer design problem and the objective function value equal to 2.99442E+03. The statistical results obtained from TNTWCOA and competitor algorithms implementation are released in Table 11. These results show that TNTWCOA has superior performance over competitor algorithms due to better values of statistical indicators.

**Table 11:** Statistical results for the speed reducer design problem. Bold values have the best performance.

Item	TNTWCOA	ICOA	COA	GJO	OOA	SCSO	SABO
Min	**2.99442E+03**	3.04113E+03	3.02284E+03	2.99657E+03	4.32890E+03	2.99490E+03	3.06911E+03
Std	**7.92159E−13**	6.62098E+02	4.14855E+13	4.22775E+00	9.35066E+13	5.06267E+00	5.61350E+02
Avg	**2.99442E+03**	3.40279E+03	2.14189E+13	3.00375E+03	1.15588E+14	3.00031E+03	4.48157E+03
Median	**2.99442E+03**	3.19760E+03	5.30711E+03	3.00305E+03	8.65666E+13	2.99848E+03	4.45055E+03
Worse	**2.99442E+03**	5.85658E+03	1.27124E+14	3.01376E+03	2.50000E+14	3.01314E+03	5.42131E+03

Significant values are in bold.

### Wilcoxon rank sum test results on engineering optimization problems

Table 12 shows the Wilcoxon statistics for TNTWCOA and 6 different algorithms over 30 runs. From the comparison of **TNTWCOA** algorithm with ICOA, GJO, COA, SCSO, OOA, SABO algorithm, there is a significant deviation between **TNTWCOA** algorithm and most of the optimization results. However, when Welded beam design Problem are optimized, the Wilcoxon rank sum test of **TNTWCOA** algorithm and **SCSO, GJO** algorithm is greater than 0.05, indicating that there is no significant difference between the obtained results.

**Table 12:** Statistical results of Wilcoxon rank-sum test.

TNTWCOA vs	ICOA	COA	GJO	OOA	SCSO	SABO
Speed Reducer	3.01986E−11	3.52006E−07	3.01986E−11	3.01986E−11	9.75550E−10	3.01986E−11
Welded beam design	1.30106E−10	2.62132E−09	**6.62576E−01**	2.95240E−11	**7.61713E−01**	5.52560E−04
Three-bar truss design problem	1.23416E−10	3.34392E−03	2.26226E−11	8.41227E−08	5.06612E−11	2.26226E−11
Gear train design Problem	1.98272E−11	1.99565E−11	1.99565E−11	1.99565E−11	1.99565E−11	1.99565E−11

Significant values are in bold.

## Conclusion

In order to improve the optimization speed and performance of Coati algorithm, a multi-strategy improved Coati algorithm is proposed, which combines chaotic sequence, nonlinear inertia weight, adaptive T-distribution variation strategy, alert update and other strategies to improve the optimization performance of the algorithm. The algorithm introduces chaotic sequence mechanism to initialize the position. The position distribution of the initial solution is more uniform, the high quality initial solution is generated, the population richness is increased, and the problem of poor quality and uneven initial solution of Coati Optimization Algorithm is solved. In the exploration phase, the nonlinear inertial weight factor is introduced to coordinate the local optimization ability and global search ability of Coati algorithm. In the exploitation phase, adaptive T-distribution variation is introduced to increase the diversity of individual population under low fitness value and improve the ability of the algorithm to jump out of the local optimal value. At the same time, the Coati alert update mechanism is proposed to improve the alert capability of the Coati algorithm, so that it can search within the optional range. When Coati is aware of the danger, the Coati on the edge of the population will quickly move to the safe area to obtain a better position, while the Coati in the middle of the population will move randomly. To be close to other Coati. In the experimental part, IEEE CEC2017 benchmark experiment is selected to test the optimization performance of TNTWCOA. In the experiment, TNTWCOA algorithm is compared with ICOA, GJO, COA, SCSO, OOA, SABO and other 6 algorithms, and the results show that TNTWCOA algorithm is better than other algorithms in terms of optimization performance. In addition, the TNTWCOA algorithm is applied to Three bar truss design, The Gear Train Design, Speed reducer and other four practical constraint projects to verify the actual optimization effect of TNTWCOA on engineering problems.

In future work, we will continue to improve TNTWCOA 's exploration capabilities and convergence rate, and apply it to the optimization design of mechanical structure components, and at the same time, optimize the accuracy of surrogate models such as KRIGING and SVR, and establish prediction models of performance and defects of mechanism structural components as well as reliability analysis models of mechanism structural components. On the basis of the prediction model or reliability analysis model, COA algorithm is used to optimize the parameters and reliability analysis of the structural components.

## Data Availability

All data generated or analyzed during this study are included in this manuscript.
